# Exosome encapsulated albumin nanoparticles target delivery of DBET6 as a treatment for triple-negative breast cancer

**DOI:** 10.1371/journal.pone.0335890

**Published:** 2026-01-12

**Authors:** Han Yu, Jingyuan Zhao, Dan Wu, Hong Yuan

**Affiliations:** 1 The Second Affiliated Hospital of Dalian Medical University, Dalian, China; 2 Central Hospital of Dalian University of Technology, Dalian, China; Brandeis University, UNITED STATES OF AMERICA

## Abstract

Triple-negative breast cancer (TNBC) is a highly aggressive disease with significant mortality, and there is an urgent need for therapies that can effectively target the disease and enhance patient survival rates. The BET family protein BRD4 plays a key role in the development and progression of TNBC. Its degrader, dBET6—a proteolysis-targeting chimera (PROTAC) molecule—shows promising anti-tumor potential but suffers from low bioavailability and poor tissue selectivity. To improve its targeted delivery efficiency, this study developed a novel nanodrug delivery system, Exo-BSA@dBET6, which encapsulates dBET6 within bovine serum albumin (BSA) nanoparticles and further coats them with milk-derived exosomes, leveraging both the natural targeting ability of exosomes and the high drug-loading capacity of BSA. The results demonstrated that Exo-BSA@dBET6 has a uniform particle size of approximately 85.89 nm, good stability, high encapsulation efficiency, and excellent biocompatibility. In vitro experiments showed that this nanosystem significantly enhanced the cellular uptake of the drug in MDA-MB-231 cells, primarily through clathrin-mediated endocytosis, and exhibited efficient lysosomal escape. Compared to free dBET6 and BSA@dBET6, Exo-BSA@dBET6 displayed stronger cytotoxicity, significantly induced apoptosis, increased reactive oxygen species (ROS) levels, reduced mitochondrial membrane potential, and up-regulated caspase-3 protein expression. Western blot analysis further confirmed that Exo-BSA@dBET6 effectively degraded BRD4 protein, down-regulated c-Myc, and up-regulated Bax expression. Transcriptome sequencing analysis indicated that the nanosystem exerts anti-tumor effects by modulating key signaling pathways such as PI3K-Akt and Rap1. This study successfully constructed an exosome-modified albumin-based nanodrug delivery system that significantly enhances the targeting and anti-TNBC efficacy of dBET6, providing a new strategy for the targeted therapy of TNBC.

## Introduction

Breast cancer is the most common malignancy among women worldwide, accounting for 32% of cancers diagnosed in women and the leading cause of cancer death among women aged 20–49 years [1]. Among these, triple-negative breast cancer (TNBC) exhibits a particularly poor prognosis. More than 50% of patients relapse within the first 3–5 years after diagnosis [2]. Due to the absence of relevant receptor markers, TNBC patients do not benefit from established endocrine therapies or HER2-targeted agents. Thus, the predominant treatment for nonsurgical TNBC remains non-specific chemotherapy. However, it is associated with significant limitations, including drug resistance, severe adverse effects, and heterogeneous treatment responses. Consequently, there is an urgent need to develop targeted therapies for the effective management of TNBC. Bromodomain and extra-terminal (BET) family proteins play a crucial role as epigenetic modifiers. As the most extensively expressed protein within the BET family, BRD4 plays a central role in regulating numerous molecular and cellular processes [3]. In recent years, several studies have demonstrated that BRD4 plays a key role in cell proliferation, survival and metastasis of breast cancer, and can be used as an emerging therapeutic target for down-regulating TNBC [4–6]. Small-molecule BET inhibitors have shown preclinical efficacy, however, their clinical application has been limited by the development of drug resistance and the occurrence of off-target effects.

To overcome these limitations, proteolysis-targeting chimera (PROTAC) technology has gained traction as a novel approach to degrade target proteins selectively. PROTAC technology is a kind of targeted protein degradation technology using ubiquititation-proteasome system (UPS), leading to the proteasomal degradation of the ubiquitinated target protein. In recent years, this technology has garnered significant attention due to its capability to target undruggable proteins [7–8]. DBET6, a PROTAC-based BRD4 degrader, effectively and continuously degrades BRD4 protein, thereby exerting potent antitumor effects, such as breast cancer, lung cancer, etc [9–10]. However, the limited tissue selectivity and low bioavailability of dBET6 significantly restrict its clinical application. Therefore, it is a critical issue to optimize the dBET6 drug delivery system.

Exosomes are cell-derived nanovesicles that play a crucial role in the intercellular transport of substances and are currently being investigated as promising endogenous nanocarriers [11–12]. Therapeutic drugs, such as small molecule or nucleic acid drugs, can be integrated into exosomes and then delivered to specific types of cells or tissues due to homing effects for targeted drug delivery [13–14]. In addition, exosomes can also be used to deliver PROTAC molecules [15–16]. Notably, A. Nathani utilized camel milk-derived exosomes as a delivery platform for ARV-825, thereby enhancing its efficacy in cancer therapy [17]. In the present study, we selected bovine milk-derived exosomes as the coating material for our nanoplatform. Milk is secreted by mammary gland cells, and consequently, milk-derived exosomes naturally carry surface proteins that resemble those of mammary tissue cells. This biological characteristic suggests their potential intrinsic targeting affinity for breast cancer cells, including TNBC. Additionally, milk exosomes offer significant advantages of biocompatibility, widespread availability, ease of acquisition, and scalability compared to cell culture-derived exosomes.

However, the poor drug loading properties of exosomes constrain their efficacy in delivering PROTAC molecules, Bovine serum albumin (BSA) have been widely explored as multifunctional nanocarriers for superior drug-carrying capacity, excellent biocompatibility and convenience of surface modiﬁcations [18–20]. However, unmodified BSA nanoparticles exhibit issues including suboptimal stability and inadequate targeting efficacy. It is worth noting that there is a complementary relationship between exosomes and albumin nanoparticles in drug delivery properties.

In this study, we combined dBET6 with albumin nanoparticles and then encapsulated these nanoparticles with readily available and easily prepared milk-derived exosomes to obtain Exo-BSA@dBET6. This approach not only enhances the in vivo targeting specificity of the albumin-based nanomaterials but also improves the drug-loading efficiency of the exosome carriers. We have confirmed that Exo-BSA@dBET6 exhibits superior cell uptake capacity and anti-tumor efficacy in vitro in breast cancer cells. Subsequently, we investigated its underlying anti-tumor mechanisms. Overall, our results indicated that the proposed Exo-BSA@dBET6 could be adopted as an efficient treatment for TNBC.

## Methods

### Preparation of milk exosome

The exosomes used in this study were derived from bovine milk, which were selected due to their mammary gland origin, potential targeting affinity for breast cancer cells, and practical advantages including biocompatibility and scalability. Centrifuge bovine milk samples at 3000 g for 30 minutes at 4 °C to remove fat globules, casein aggregates, and other debris. Carefully collect the supernatant and subject it to centrifugation at 10,000 g for 60 minutes at 4 °C to eliminate larger cellular debris and vesicles. Following this, collect the supernatant and filter it through a 0.22 μm membrane filter. Subsequently, perform ultracentrifugation at 100,000 g for 120 minutes at 4 °C to pellet the exosomes. Discard the supernatant, and promptly wash the exosome pellet with phosphate-buffered saline (PBS) to remove residual debris and microvesicles. Finally, collect the purified exosome pellet, resuspend it in a small volume of PBS, and store it at −80 °C for subsequent analysis.

### Preparation of BSA@dBET6

A defined quantity of BSA was dissolved in 2 mL of water, and the pH was adjusted to 8.0–9.0 using a 3 mol/L sodium hydroxide solution, dBET6 was dissolved in anhydrous ethanol and then added to the BSA solution at a controlled rate of 0.2 mL/min (drug-to-BSA mass ratio is 1:15). Subsequently, anhydrous ethanol was added dropwise such that the final volume ratio of water to anhydrous ethanol reached 1:4, while the solution was continuously stirred at 600–800 rpm. A solution of BAC in anhydrous ethanol was then introduced, with a BSA-to-BAC mass ratio of 10:1. The resulting mixture was stirred continuously at 50 °C for 24 hours. Afterward, the ethanol was removed via evaporation, and the product was purified by centrifugation to yield BSA@dBET6.

### Preparation of Exo-BSA@dBET6

Dissolve 10 mg of BSA@dBET6 in 2 mL of PBS solution at pH 7.4, and disperse the mixture uniformly via ultrasonication. Subsequently, add 1 mL of milk-derived exosomes and thoroughly mix the solution. Place the resulting mixture in an ice bath and subject it to ultrasonic cell disruption under controlled conditions (2 kHz, 10% amplitude, 3 cycles of 3 minutes each with 2-minute intervals between cycles). Following ultrasonication, incubate the sample at 37 °C for 1 hour. Centrifuge the sample at 10,000 rpm for 5 minutes at 4 °C, discard the supernatant, and wash the pellet with PBS buffer three times. The final product, designated as Exo-BSA@dBET6, is obtained and subsequently freeze-dried for long-term storage.

### Characterization of nanoparticle

The morphologie of Exo-BSA@dBET6 were observed through transmission electron microscopy (TEM): Exo-BSA@dBET6 sample (10 microliters) was negatively stained with 2% phosphotungstic acid for 1 minute, air-dried, and then applied to an EM grid. Observations were carried out using a transmission electron microscope operated at a voltage of 100 kV.

Use Dynamic Light Scattering (DLS) to assess particle size, PDI, and Zeta potential: The prepared Exo-BSA@dBET6 solution was diluted fivefold, and the particle size distribution, PDI, and Zeta potential of Exo-BSA@dBET6 were determined by DLS, using a Zetasizer Nano ZS equipment (Malvern Instruments, UK).

The encapsulation efficiency (EE) and drug loading (DL) of Exo-BSA@dBET6 were determined using an ultrafiltration-centrifugation method combined with UV-Vis spectrophotometry. The EE and DL were calculated using the following equations:


$$EE(\%)=(W_{total}-W_{free})/\;W_{total}\times \text{1}\text{0}\text{0}\%$$ (1)

Where EE is the encapsulation efficiency, Wₜₒₜₐₗ is the total amount of drug added during the preparation process, and Wfᵣₑₑ is the amount of unencapsulated free drug measured in the supernatant*.*


$$DL(\%)=(W_{total}-W_{free})/\;W_{nanoparticles}\times \text{1}\text{0}\text{0}\%$$ (2)

Where DL is the drug loading capacity, Wₜₒₜₐₗ is the total amount of drug added, Wfᵣₑₑ is the amount of unencapsulated free drug, and W_nanoparticles_ is the total weight of the nanoparticles obtained.

The nanoparticles underwent SDS-PAGE electrophoresis and were stained with Coomassie Brilliant Blue to identify their composition from albumin. The western blotting (WB) assay was used to verify the levels of two specific exosome markers (CD9 and CD63) in exosomes within the nanoparticles, in order to confirm the successful encapsulation of exosomes in albumin nanoparticles.

### Cell culture

MDA-MB-231 cell lines were cultured in DMEM medium containing 10% fetal bovine serum (FBS) and 1% antibiotic mixture (streptomycin and penicillin). All cells were incubated at 37 ° C in a humidified atmosphere of 5% carbon dioxide.

### Hemolysis test

Take a certain amount of defibrinated sheep blood (Solabio, Cat: TX0030), centrifuge at 400 g for 10 minutes, and then separate and retain the red blood cells. Then resuspend the red blood cells with an excess of normal saline, centrifuge at 400 g for 15 minutes, and repeat this step until the upper layer of normal saline after centrifugation is clear and transparent. Subsequently, resuspend with an appropriate amount of normal saline to obtain a 4% red blood cell suspension. Add 200 μL of red blood cell suspension and 200 μL of the corresponding concentration of the drug (normal saline as the negative control and 0.1% Triton as the positive control) to 1.5 mL EP tubes, mix well, and incubate at 60 rpm for 30 minutes at 37 ° C. Centrifuge at 400 g for 10 minutes and take photos for record. Then, take 100 μL of the supernatant into a 96-well plate and measure the OD value at a wavelength of 480 nm.

### Cellular uptake and pathway of Exo-BSA@dBET6

First, prepare FITC-labeled nanoparticles. Accurately weigh a specific amount of BSA@dBET6 and Exo-BSA@dBET6, dissolve it in phosphate-buffered saline (pH = 7.4). Add an appropriate amount of FITC. The mixture was stirred away from light for 12 hours. Subsequently, free FITC was removed using an ultrafiltration tube, yielding FITC-labeled BSA@dBET6 and Exo-BSA@dBET6 complexes.

### Flow cytometry

The intracellular uptake of Exo-BSA@dBET6 was quantified using flow cytometry. MDA-MB-231 cells (10^5^ cells/well) were seeded in a 12-well plate and co-incubated with FITC-stained BSA@dBET6 and Exo-BSA@dBET6 for 24 hours. Subsequently, the cells were harvested and resuspended in 300 μL of pre-chilled PBS for flow cytometric analysis.

### Fluorescence microscope

MDA-MB-231 cells were seeded in a 6-well plate at a density of 2 × 10^5^ cells per well and incubated overnight at 37°C. Subsequently, the cells were treated with FITC-stained BSA@dBET6 or Exo-BSA@dBET6 for 24 hours. After treatment, wash the cells twice with 1 × PBS, fix them with 4% paraformaldehyde for 30 minutes, and then stain the cell nuclei with DAPI for 5 minutes. Finally, observe the cellular uptake under a fluorescence microscope.

To investigate the cellular uptake pathway of Exo-BSA@dBET6, MDA-MB-231 cells were preincubated with three endocytic inhibitors (Amiloride and M-β-C) for 30 min. Then FITC-stained Exo-BSA@dBET6 was added and incubated for an additional 24h. Finally, observe the cellular uptake under a fluorescence microscope.

### Cytotoxicity assay

Cytotoxicity was assessed using the CCK-8 assay. MDA-MB-231 cells were seeded in 96-well plates at a density of 1 × 10^4^ cells per well and incubated at 37°C for 24 hours. Subsequently, the cells were treated with free dBET6, BSA@dBET6, and Exo-BSA@dBET6 for an additional 24 hours. Following treatment, the medium was removed, and 100 μL of CCK-8 solution (10%) was added to each well, followed by an additional incubation period of 30 minutes. Finally, absorbance (OD) values were measured at a wavelength of 450 nm using a microplate reader. All experiments were performed with at least three independent replicates (n ≥ 3).

### Western blotting assay

RIPA buffer containing PMSF was used to extract total protein from the treated cells. SDS-PAGE was performed to separate protein bands, which were then transferred to a polyvinylidene difluoride (PVDF) membrane. Following 1 h blocking step with 5% bovine serum albumin (BSA) in phosphate buffered saline with Tween (PBST), the PVDF membrane was incubated overnight at 4°C with CD9 antibody (Proteintech, 20597–1-AP), CD63 antibody (Proteintech, 25682–1-AP), C-Myc antibody (Proteintech, 10828–1-AP), Bax antibody (Proteintech, MA5–14003), GAPDH antibody (Proteintech, MA5–15738). After three washes, the membrane was incubated with horseradish peroxidase-conjugated secondary antibodies at room temperature for 1 h. Finally, enhanced chemiluminescence was employed as directed to observe the immunoblot.

### Living and dead staining

To evaluate the viability of cells, living and dead cells were stained with Calcin-AM and PI (Beyotime, C2015M). Following drugs treatment, cells were washed three times with PBS. The staining solution was then added to the cell culture dish and incubated for 30 minutes. Subsequently, cell fluorescence was examined using a fluorescence microscope (Leica) and images were captured for further analysis.

### ROS staining

DCFH-DA is prepared by diluting it at a ratio of 1:1000 in serum-free medium. Following drug treatment, the cell culture medium is carefully aspirated and replaced with the diluted DCFH-DA solution at a concentration of 1:1000 in each well. The cells are then incubated at 37°C for 20 minutes. Subsequently, the cells are washed three times with PBS to ensure complete removal of unbound DCFH-DA. Finally, the presence of reactive oxygen species (ROS) is detected using fluorescence microscopy.

### JC-1 staining

Mitochondrial membrane potential (MMP) was assessed using the JC-1 assay. MDA-MB-231 cells treated with free dBET6 or Exo-BSA@dBET6 were incubated with JC-1 (diluted 1:1000) at for 20 minutes. Following PBS washes, the cells were examined under a fluorescence microscope to capture red fluorescence and green fluorescence.

### Transcriptome sequencing and differentially expressed genes analysis

Total RNA was extracted from MDA-MB-231 cells, whole transcriptome sequencing and bioinformatics data were analyzed at GENEWIZ(China). The differentially expressed genes (DEGs) from the RNA sequencing results were analyzed after transcriptome sequencing. Enrichment analysis was performed by GO and KEGG to track the biological ability of overlapping target genes. The results include cellular component (CC), biological process (BP), andmolecular function (MF) discovered by GO analysis and key signaling pathways generated by KEGG analysis.

### Statistical analysis

Analysis was conducted using a one-way analysis of variance or the unpaired Student’s t-test. All statistical analysis were performed using GraphPad Prism 8 software (GraphPad Software, San Diego, CA, USA), and p < 0.05 suggested that the difference was statistically significant. Data are presented as mean ± standard deviation (SD) from at least three independent experiments.

## Results

### Characterization and Biocompatibility of Exo-BSA@dBET6

**Fig 1:**
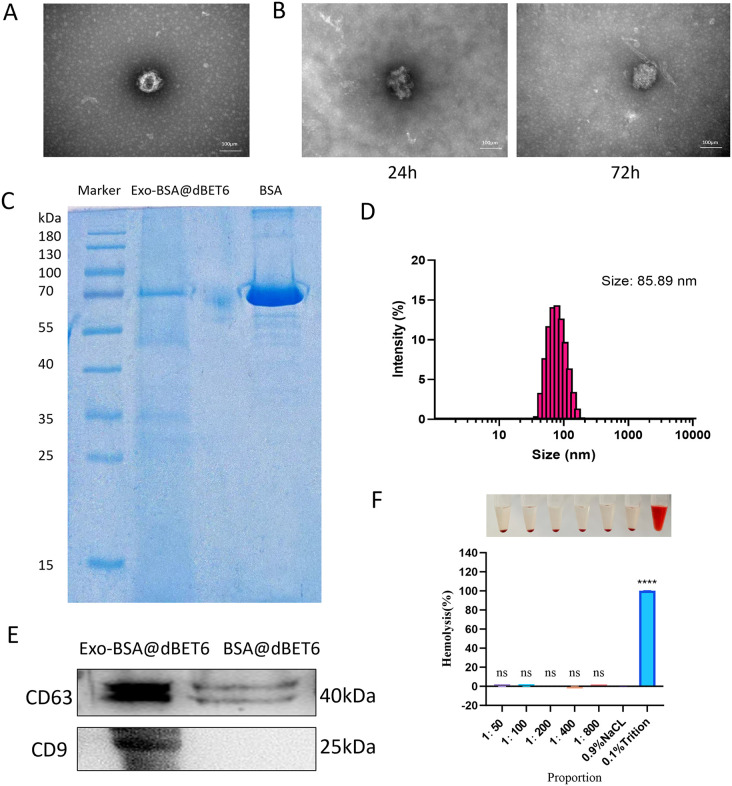
Characterization and biocompatibility of Exo-BSA@dBET6. **(A)**Morphology of Exo-BSA@dBET6 under TEM. (scale bar: 100 nm) **(B)** Stability evaluation of Exo-BSA@dBET6 in PBS. TEM images of Exo-BSA@dBET6 nanoparticles after incubation in pH 7.4 PBS at 37 °C for 24 hours and 72 hours. **(C)** The nanoparticles were subjected to SDS-PAGE electrophoresis and Coomassie Brilliant Blue staining. **(D)** DLS size distribution of Exo-BSA@dBET6. **(E)** WB analysis showing the expression of two specific exosomal markers (CD9, CD63) in exosomes. **(F)** Hemolysis test of Exo-BSA@dBET6 at various concentrations. Saline and 0.1% Triton X-100 were used as negative and positive controls, respectively. No significant hemolysis was observed in the nanoparticle groups.

As shown in Fig 1a, in the transmission electron microscope (TEM) image, Exo-BSA@dBET6 presents a spherical vesicle structure. As shown in Fig 1d, the average diameter of Exo-BSA@dBET6 is 85.89nm. TEM provides precise measurements of individual particle dimensions, thereby revealing detailed morphological characteristics. In contrast, DLS yields an intensity-weighted hydrodynamic diameter that represents the average size of the entire particle population in solution. The observed discrepancy is further supported by the sample’s polydispersity index (PDI = 0.275). The zeta potential is −30 mV, which suggests that the particle can remain stably dispersed in an aqueous solution over an extended period, and the polydispersity index (PDI) is 0.275, suggesting a relatively homogeneous size distribution. The encapsulation efficiency (EE) of Exo-BSA@dBET6 is 56%, and the drug loading (DL) capacity is 2.7%.

To evaluate the stability of Exo-BSA@dBET6, we selected phosphate buffered saline (PBS) with a pH of 7.4 as the incubation medium to simulate physiological conditions. The reason for not choosing plasma was to avoid interference from abundant plasma proteins, which might interfere with the quality of TEM imaging and affect the recognition of nanoparticles. Under the physiologically relevant conditions, Exo-BSA@dBET6 was incubated at 37°C for 24 hours and 72 hours, and then examined by TEM. As shown in Fig 1b, at both time points, the nanoparticles maintained their structural integrity without obvious morphological changes, indicating their excellent stability. These results suggest that Exo-BSA@dBET6 has strong stability.

The nanoparticles were analyzed by SDS-PAGE electrophoresis and Coomassie Blue staining (Fig 1c), indicating that the main component of the nanoparticles has a molecular weight similar to that of BSA. Additionally, the exosome-coated nanoparticles exhibited more bands corresponding to exosome-associated proteins compared to BSA. WB analysis also detected these exosomal markers (CD63 and CD9) (Fig 1e), indicating that we successfully constructed BSA nanoparticles coated with exosomes. To evaluate the hematocompatibility of Exo-BSA@dBET6, a hemolysis assay was performed using sheep red blood cells. The nanoparticles were incubated with erythrocytes at various concentrations, and the hemolytic activity was quantitatively and qualitatively assessed. As shown in Fig 1f, no significant hemolysis was observed at any tested concentration, confirming the excellent blood compatibility of Exo-BSA@dBET6.

### Cellular uptake and pathway of Exo-BSA@dBET6

**Fig 2:**
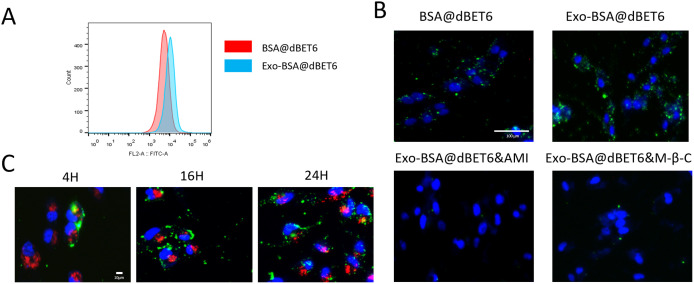
Cellular uptake and pathway of Exo-BSA@dBET6. **(A)** FC analysis of cellular uptake, red, blue histograms are denoted the dBET6@BSA and dBET6-BSA@exosome nanovehicles, respectively. **(B)** Fluorescence microscope images of MDA-MB-231 cells co-cultured with BSA@dBET6, Exo-BSA@dBET6 nanovehicles and pathway inhibitors (amiloride and M-β-C) for 24h. **(C)** The lysosomal escape effect of MDA-MB-231 cells detected after incubation with Exo-BSA@dBET6 for 4h, 16h, and 24h. Red fluorescence indicates the presence of lysosomes, while green fluorescence corresponds to Exo-BSA@dBET6.

To investigate the tumor-targeting capability of Exo-BSA@dBET6, we evaluated their intracellular uptake behavior using fluorescence microscopy and flow cytometry. As shown in Fig 2b, fluorescence microscopy analysis reveals that the fluorescence intensity in MDA-MB-231 cells treated with Exo-BSA@dBET6 nanocarriers is significantly higher compared to those treated with BSA@dBET-6 at the same time point. In addition, as shown in Fig 2a, the green fluorescence signal was further investigated by FC analysis, showing a trend consistent with that observed under fluorescence microscopy. Taken together, the intracellular uptake of Exo-BSA@dBET6 was increased compared with BSA@dBET6.

We used amiloride (AMI) and M-β-C as pathway inhibitors to investigate the associated clathrin-dependent and caveolin-dependent uptake of Exo-BSA@dBET6. As shown in Fig 2b, AMI inhibited the uptake of Exo-BSA@dBET6 by breast cancer cells better than M-β-C. This suggests that clathrin-mediated endocytosis may be the main pathways of Exo-BSA@dBET6 uptake by breast cancer cells.

Currently, one of the principal challenges in nanomedical drug delivery systems is preventing the lysosomal degradation of both the carrier and the encapsulated drug. Consequently, we conducted a detailed evaluation of the lysosomal escape efficiency of Exo-BSA@dBET6. As illustrated in Fig 2c, the lysosomal escape effect was assessed at 4 hours, 16 hours, and 24 hours post-treatment with Exo-BSA@dBET6. The results demonstrate that the green fluorescence of Exo-BSA@dBET6 is distinctly visible outside the lysosomes, indicating successful lysosomal escape.

### Antitumor activities in vitro

**Fig 3:**
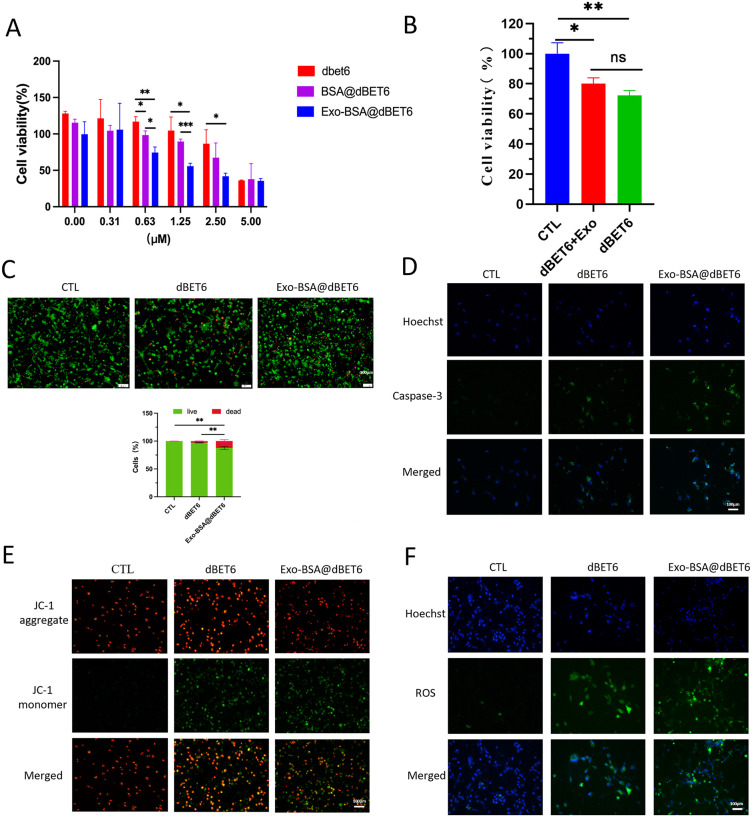
Antitumor activities of Exo-BSA@dBET6. **(A)** CCK8 assay results showed that Exo-BSA@dBET6 has the most significant inhibitory effect on the growth and proliferation of TNBC cells. Data are presented as mean ± SD (n = 3). *p < 0.05, **p < 0.01, ***p < 0.001 vs. control group. **(B)** CCK8 assay showed that there was no statistically significant difference in cytotoxicity between the free dBET6 group and the free exosomes + free dBET6 physical mixture group (p > 0.05). Data are presented as mean ± SD (n = 3). NS: not significant. **(C)** Calcein-AM/PI staining demonstrated that Exo-BSA@dBET6 was significantly more effective in promoting tumor cell death compared to dBET6. **(D, E)** Caspase-3 staining and JC-1 staining results showed that Exo-BSA@dBET6 has the capability to significantly enhance apoptosis of MDA-MB-231 cells. **(F)** The ROS staining results showed that the ROS concentration in Exo-BSA@dBET6 group was significantly increased compared with dBET6 group.

The above experiments have confirmed that exosomes encapsulation can enhance the cellular uptake of albumin nanoparticles. We then performed a series of activity experiments to verify the anti-tumor ability in vitro. As shown in Fig 3a, compared with the control group, the cell viability of dBET6, BSA@dBET6 and Exo-BSA@dBET6 groups was decreased in a dose-dependent manner. At the same concentration, Exo-BSA@dBET6 group had the best effect on cell viability, followed by BSA@dBET6 group, and dBET6 had the worst effect. To exclude the potential influence of simple physical mixing, a control group of free exosomes + free dBET6 (physical mixture) was included in the cytotoxicity assessment. As shown in Fig 3b, no statistically significant difference in cell viability was observed between the free dBET6 group and the free exosomes + free dBET6 physical mixture group (p > 0.05), indicating that simple physical mixing does not enhance the antitumor efficacy of dBET6, and conversely confirms that the superiority of Exo-BSA@dBET6 nanoparticles stems from their unique structural design rather than mere mixing of the components. Furthermore, the enhanced ability of Exo-BSA@dBET6 to promote tumor cell death compared with free dBET6 was confirmed by live and dead cell staining and the quantitative analysis of red and green fluorescence (p < 0.01) (Fig 3c).

To further investigate the anti-tumor efficacy of Exo-BSA@dBET6, we conducted the following experiments. First, the pro-apoptotic protein caspase-3 was labeled by specific fluorescent, as shown in Fig 3d, the level of caspase-3 in MDA-MB-231 cells treated with Exo-BSA@dBET6 was significantly elevated compared to the dBET6 group. When apoptosis occurs in cells, the mitochondrial membrane potential decreases. In normal cells, under the effect of the high mitochondrial membrane potential, JC-1 aggregates to form polymers and emits red fluorescence. In apoptotic cells, the mitochondrial membrane potential is reduced, and JC-1 exists in the form of monomers, emitting green fluorescence. The apoptotic status of cells can be judged by the change in fluorescence color and the ratio of green JC-1 monomers to red JC-1 aggregates intensity. As illustrated in Fig 3e, the green-to-red fluorescence intensity ratio of the Exo-BSA@dBET6 group is significantly higher compared to that of the free dBET6 group. These findings suggest that Exo-BSA@dBET6 exhibits a more pronounced capability in promoting apoptosis. Intracellular reactive oxygen species (ROS) in MDA-MB-231 cells treated with different formulations were observed with fluorescence microscope, using a ROS probe (DCFH-DA). As shown in Fig 3f, the ROS levels in MDA-MB-231 cells treated with Exo-BSA@dBET6 were significantly higher compared to those treated with free dBET6. These findings indicate that Exo-BSA@dBET6 can enhance the expression of the critical apoptosis protein caspase-3, decrease mitochondrial membrane potential, and promote reactive oxygen species (ROS) production by inhibiting BRD4 protein expression, ultimately exerting a pro-apoptotic effect. Furthermore, compared to free dBET6, Exo-BSA@dBET6 demonstrates a more pronounced pro-apoptotic efficacy. Collectively, Exo-BSA@dBET6 can exert anti-tumor effects by inhibiting BRD4 protein expression, and modification of dBET6 with exosomes and albumin nanoparticles markedly enhanced its anti-tumor efficacy in vitro.

### Antitumor mechanism of Exo-BSA@dBET6

**Fig 4:**
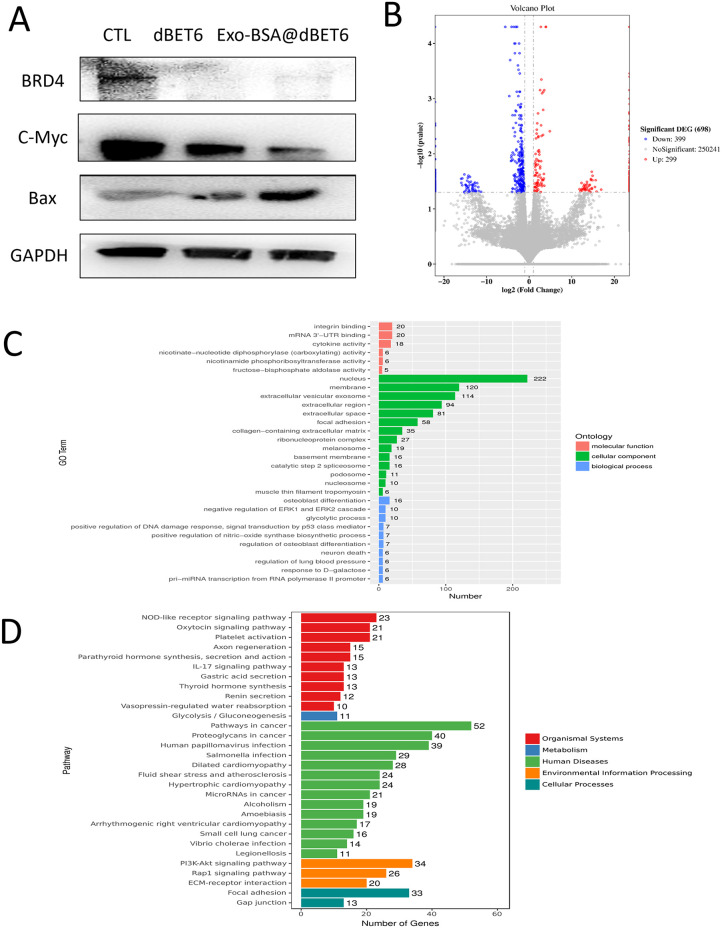
Antitumor mechanism of Exo-BSA@dBET6. **(A)** The expression of apoptotic proteins under dBET6 and Exo-BSA@dBET6 was detected by WB. **(B)** Volcano plot of the different expressed genes. **(C)** GO classification of the target genes of Exo-BSA@dBET6. **(D)** KEGG enrichment analsysis of the target genes of Exo-BSA@dBET6.

Next, we investigated the antitumor mechanism of Exo-BSA@dBET6 on TNBC in MDA-MB-231 cells. Given that BRD4, an epigenetic regulatory protein, has been confirmed in previous studies to be involved in multiple signaling pathways [21–23], we employed Western blot analysis to examine the expression levels of apoptosis-related proteins. As shown in Fig 4a, under the treatment with dBET6 and Exo-BSA@dBET6, the expression levels of anti-apoptotic proteins BRD4 and c-Myc were significantly down-regulated, while pro-apoptotic proteins Bax was markedly up-regulated. Notably, the effect of Exo-BSA@dBET6 was more pronounced compared to that of free dBET6.

To further investigate the potential biological mechanisms of Exo-BSA@dBET6 in anti-tumor activity, RNA sequencing was conducted on breast cancer cells in both experimental and control groups. As shown in the volcano plot in Fig 4b, the treatment process involved 299 up-regulated genes and 399 down-regulated genes. Gene Ontology (GO) enrichment analysis was conducted on the selected differentially expressed genes to examine changes in gene expression patterns in MDA-MB-231 cells treated with Exo-BSA@dBET6. In terms of molecular function, the targets were associated with integrin binding and mRNA 3′-UTR binding. Within the cellular component category, these targets were mainly associated with the nucleus and membrane. Regarding biological processes, the predicted targets were predominantly associated with the osteoblast differentiation process and negative regulation of ERK1 and ERK2 cascade (Fig 4c). KEGG enrichment analysis was used to further investigate the potential pathways of Exo-BSA@dBET6. As illustrated in Fig 4d, KEGG pathway analysis revealed that critical gene regulatory pathways associated with breast cancer occurrence, progression, metastasis, and prognosis, including the PI3K-Akt signaling pathway and the Rap1 signaling pathway, were significantly affected during Exo-BSA@dBET6 treatment. In summary, Exo-BSA@dBET6 regulate a series of apoptosis-related proteins and breast cancer-related signaling pathways by down-regulating BRD4 protein, and finally play an anti-TNBC tumor role.

## Discussion

Given the high expression of BRD4 in TNBC and its correlation with tumor malignancy and prognosis, BRD4 degraders have been extensively investigated for the treatment of TNBC [24–26]. As a PROTAC molecule capable of targeting the BRD4 protein in TNBC, dBET6 exhibits anti-tumor properties and holds promise for overcoming resistance to traditional chemotherapy drugs in TNBC. However, it encounters challenges such as low bioavailability and inadequate tissue selectivity during clinical translation. To address these issues, we propose utilizing a nanomedical drug delivery system.

The ideal nanomedicine delivery system should exhibit robust targeting, excellent stability, and minimal side effects. Exosomes possess low immunogenicity, high physicochemical stability, strong tissue penetration, and inherent transport capabilities, making them promising candidates for a new generation of drug delivery vehicles. However, exosomes have limitations in terms of drug loading efficiency. Consequently, hybrid exosomes have garnered significant attention in recent years. By integrating exosomes with other biological or synthetic materials through fusion, modification, or functionalization, the resultant carriers can combine the advantages of exosomes and complementary components to enhance drug delivery efficacy. Notably, exosome-liposome hybrid nanoparticles have been utilized to deliver CRISPR-Cas9 systems for targeted gene editing [27]; hybrid nanovesicles combining CAR-T cell-derived exosomes and lung-targeted liposomes have been employed to deliver paclitaxel for lung cancer treatment [28]. Most of the previous studies have used liposomes to hybridize exosomes, but the stability of liposomes is limited, and their preparation process is both complex and expensive. Albumin nanoparticles exhibit excellent biocompatibility, high binding affinity, remarkable stability, abundant sources, and relatively low preparation costs, rendering them ideal candidates for drug delivery systems [29]. Paclitaxel-loaded albumin (nab™ -paclitaxel Abraxane®) has been approved by the FDA for the treatment of metastatic breast cancer after failure of combination chemotherapy [30].

In this study, bovine milk-derived exosomes were utilized for the first time to encapsulate albumin nanoparticles, with dBET6 loaded within. By leveraging the synergistic effects of both components, we harnessed the homing properties of exosomes to enhance the targeting and tissue selectivity of the hybrid nanoparticles, while exploiting the high drug-binding capacity of albumin nanoparticles to improve their drug-loading efficiency. This approach maximizes the advantages in targeted drug delivery, ultimately achieving the targeted delivery of dBET6.

In our study, we developed a hybrid nanoparticle system designated as Exo-BSA@dBET6 and demonstrated that exosome modifications significantly enhance the uptake of drugs by tumor cells. Exo-BSA@dBET6 can elevate cellular ROS levels, reduce mitochondrial membrane potential, and enhance the expression of the apoptotic protein caspase-3 by specifically targeting the BRD4 protein, ultimately inducing tumor cell apoptosis. Additionally, its anti-tumor efficacy surpasses that of the free dBET6 molecule, demonstrating the effectiveness of our constructed nanomedicine delivery system in enhancing drug potency. Notably, the physical mixture of free exosomes and free dBET6 failed to enhance the cytotoxicity compared to free dBET6 alone, further confirming that the superior antitumor efficacy of Exo-BSA@dBET6 is attributed to the effective encapsulation of dBET6 within the BSA core and the subsequent exosome coating, rather than a simple co-delivery effect. The anti-TNBC mechanism of Exo-BSA@dBET6 is to target the BRD4 protein, subsequently, it downregulates anti-apoptotic protein c-Myc, upregulates pro-apoptotic protein Bax, and blocks the PI3K-Akt signaling pathway and the Rap1 signaling pathway.

The major limitation of the present study is the lack of animal experiments, future research should be undertaken to explore the anti-tumor effect of Exo-BSA@dBET6 in vivo. Additionally, while our transcriptomic analysis revealed significant alterations in key signaling pathways such as PI3K-Akt and Rap1, the lack of validation through qPCR or functional studies represents a limitation of this study. Future work will include experimental validation of these findings through pathway inhibition assays and gene expression analysis to provide deeper mechanistic insights.

Our findings demonstrate that the encapsulation of dBET6 within albumin nanoparticles coated with exosomes can significantly enhance the anti-tumor efficacy of dBET6, potentially addressing the therapeutic challenges associated with TNBC.

## Supporting information

S1 File The original experimental data of Fig 1 and Fig 2. (ZIP)

S2 File The original experimental data of Fig 3a, Fig 3b and Fig 3c. (ZIP)

S3 File The original experimental data of Fig 3d, Fig 3e and Fig 3f. (ZIP)

S4 File The original experimental data of Fig 4. (ZIP)

S1 Raw images Raw blot and gel images. (PDF)
