# Supplementary figures and images for: Exosome encapsulated albumin nanoparticles target delivery of DBET6 as a treatment for triple-negative breast cancer (part 1 of 2)
Source: PLoS One. 2026 Jan 12;21(1):e0335890. doi: 10.1371/journal.pone.0335890 (PMC12795375; doi:10.1371/journal.pone.0335890)

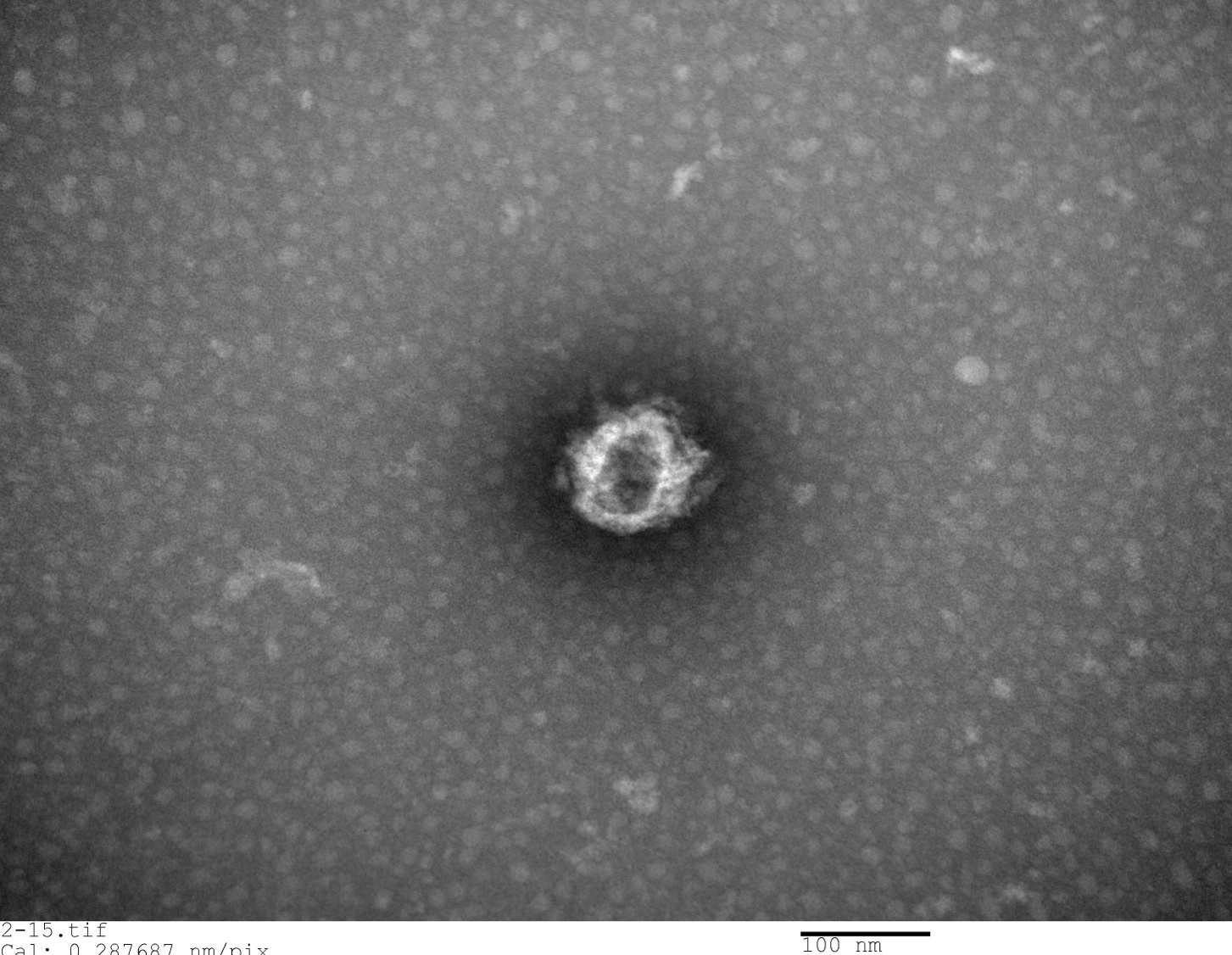

Supplement: S1 File — (ZIP) [file pone.0335890.s001.zip › Supporting Information1/Fig1/Fig1a/fig1a.jpg]

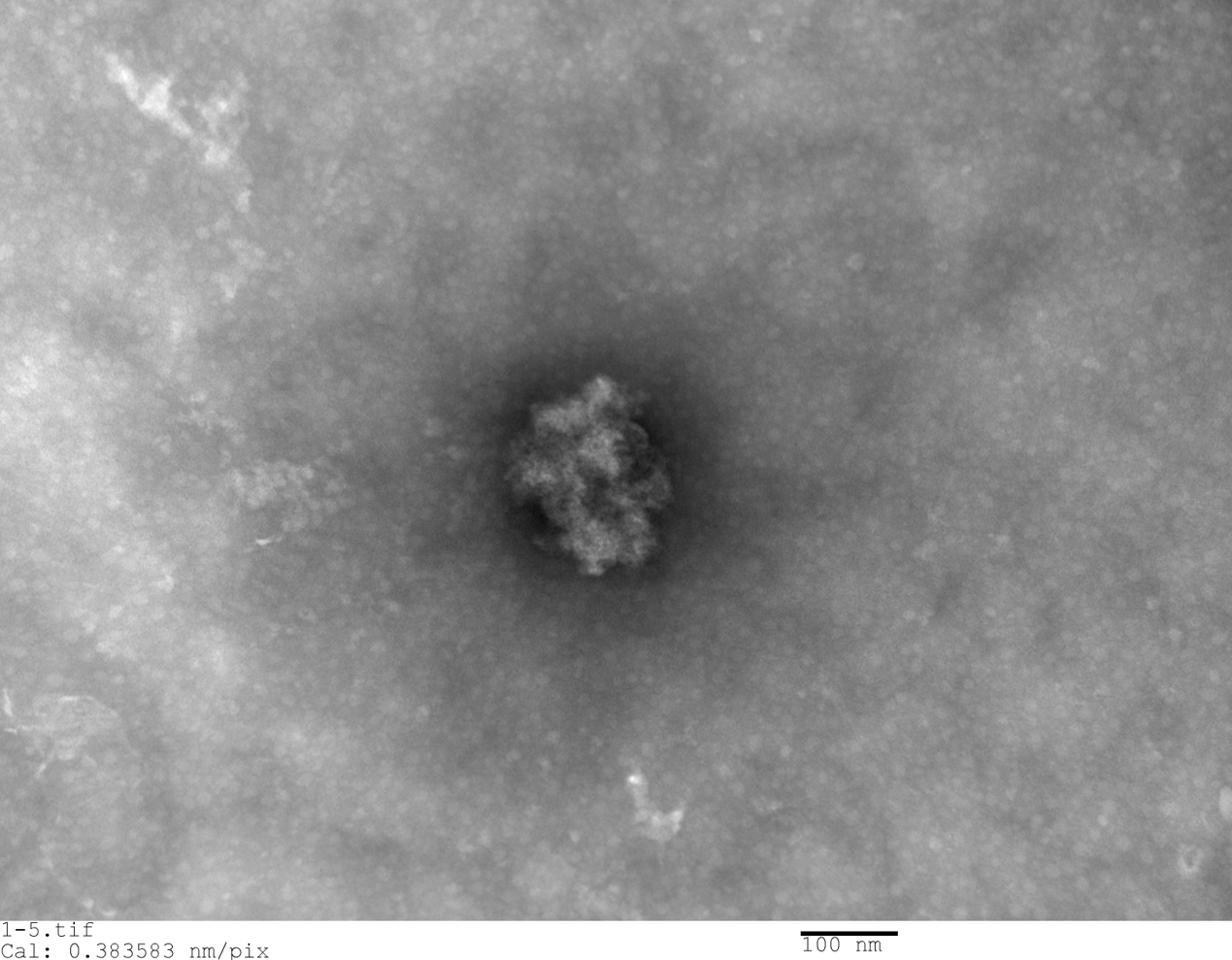

Supplement: S1 File — (ZIP) [file pone.0335890.s001.zip › Supporting Information1/Fig1/Fig1b/fig1b-24h.png]

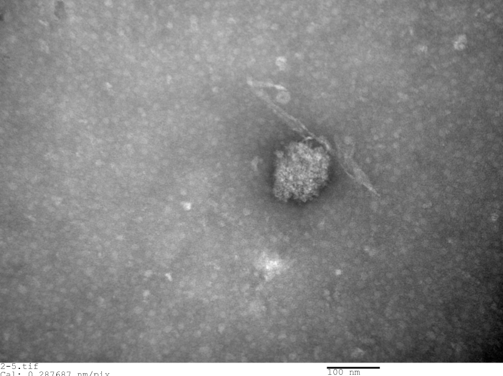

Supplement: S1 File — (ZIP) [file pone.0335890.s001.zip › Supporting Information1/Fig1/Fig1b/fig1b-72h.png]

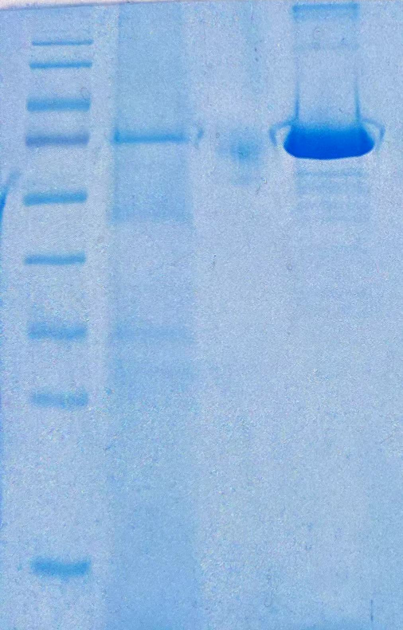

Supplement: S1 File — (ZIP) [file pone.0335890.s001.zip › Supporting Information1/Fig1/Fig1c/fig1c.png]

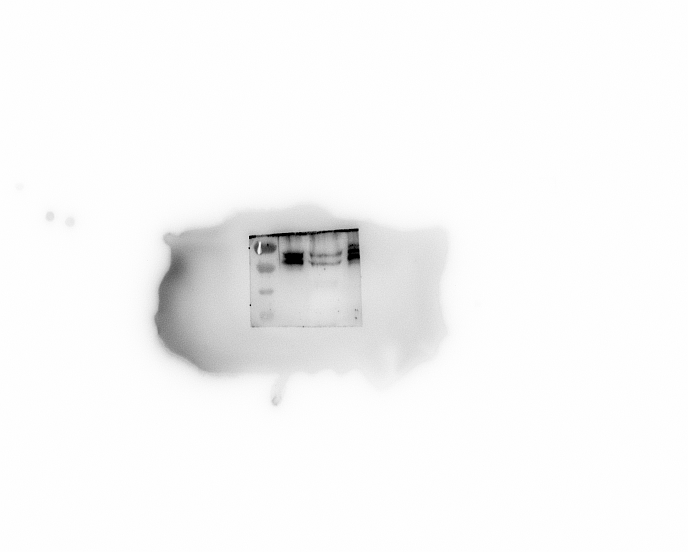

Supplement: S1 File — (ZIP) [file pone.0335890.s001.zip › Supporting Information1/Fig1/Fig1e/CD63 ..tif]

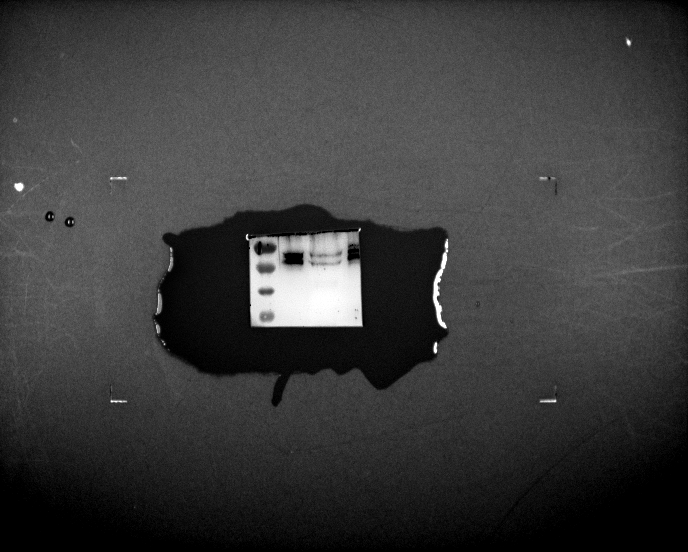

Supplement: S1 File — (ZIP) [file pone.0335890.s001.zip › Supporting Information1/Fig1/Fig1e/CD63.tif]

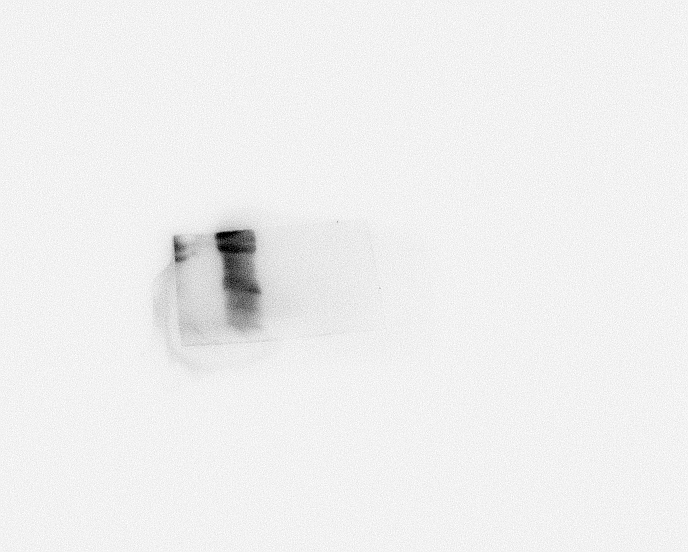

Supplement: S1 File — (ZIP) [file pone.0335890.s001.zip › Supporting Information1/Fig1/Fig1e/CD9 ..tif]

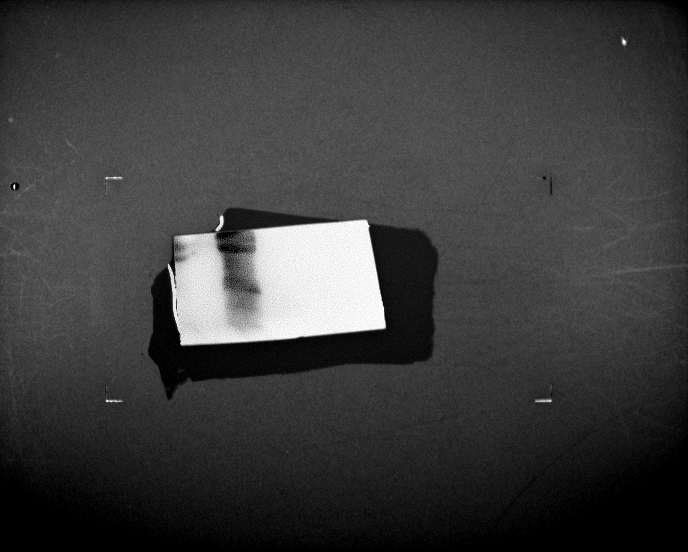

Supplement: S1 File — (ZIP) [file pone.0335890.s001.zip › Supporting Information1/Fig1/Fig1e/CD9.tif]

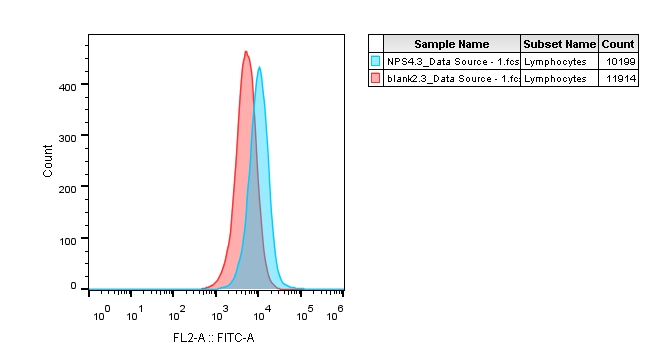

Supplement: S1 File — (ZIP) [file pone.0335890.s001.zip › Supporting Information1/Fig2/Fig2a/20241009-Layout.jpg]

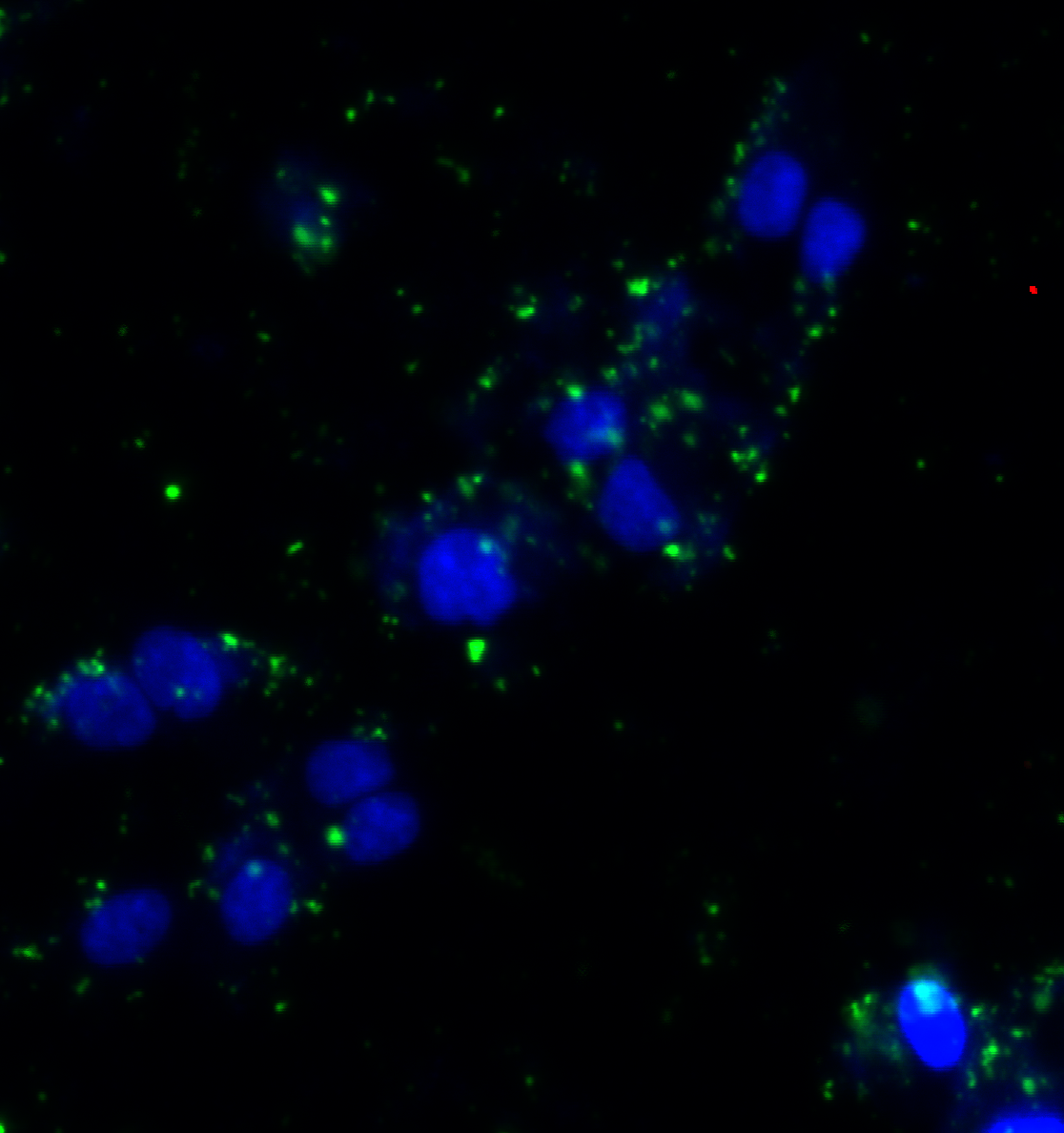

Supplement: S1 File — (ZIP) [file pone.0335890.s001.zip › Supporting Information1/Fig2/Fig2b/BSA@dBET6.tif]

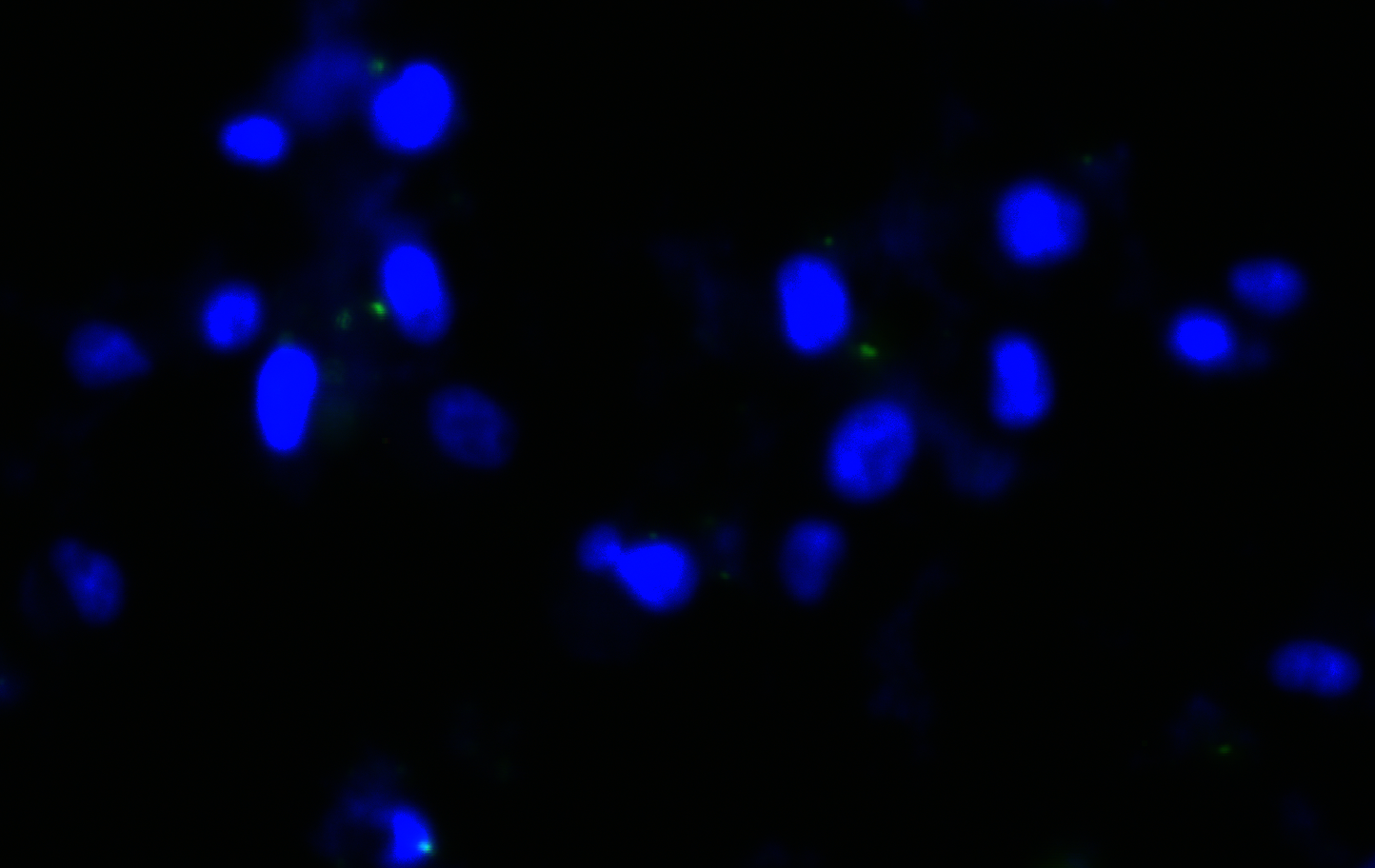

Supplement: S1 File — (ZIP) [file pone.0335890.s001.zip › Supporting Information1/Fig2/Fig2b/Exo-BSA@dBET6&AMI.tif]

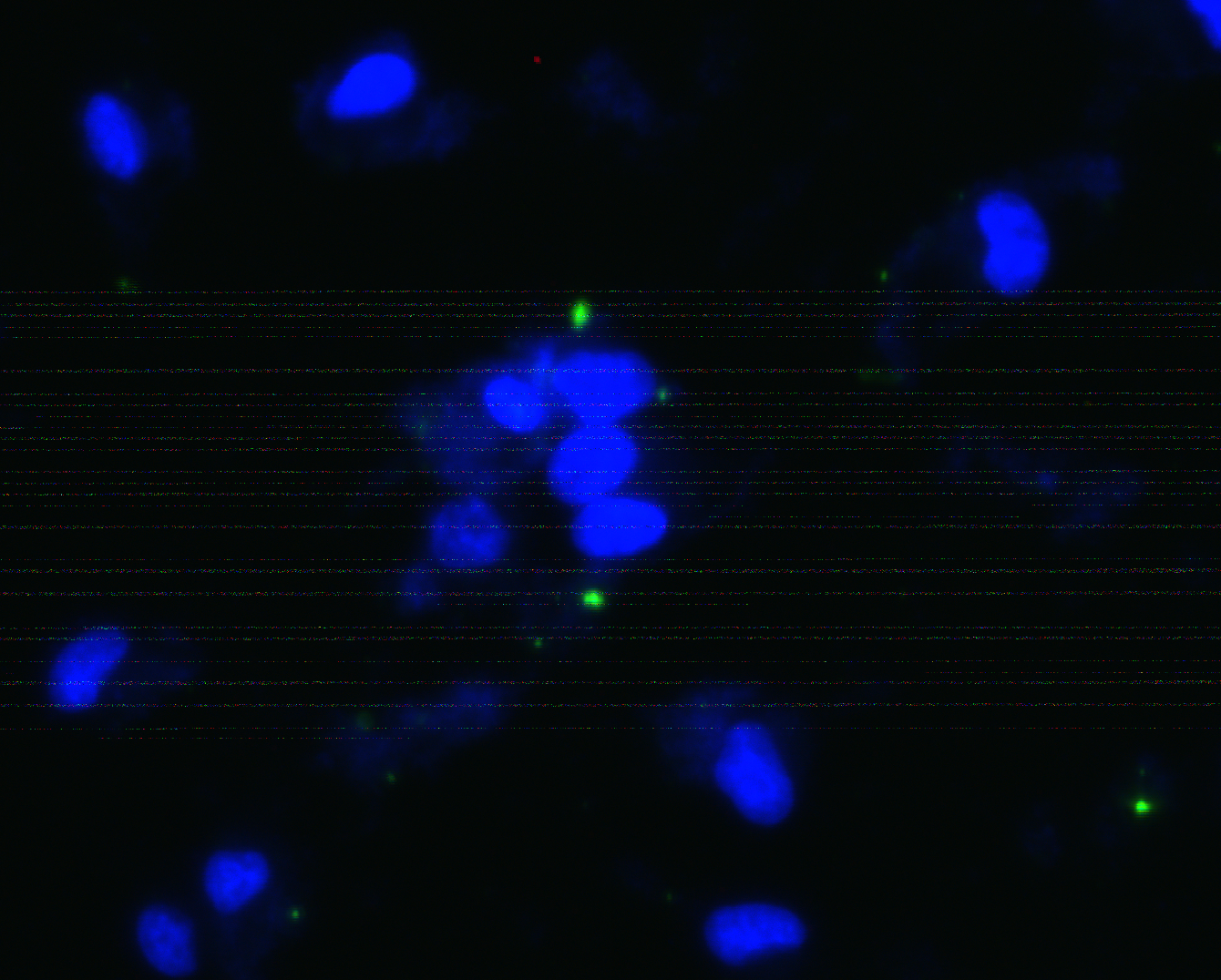

Supplement: S1 File — (ZIP) [file pone.0335890.s001.zip › Supporting Information1/Fig2/Fig2b/Exo-BSA@dBET6&M-β-C.tif]

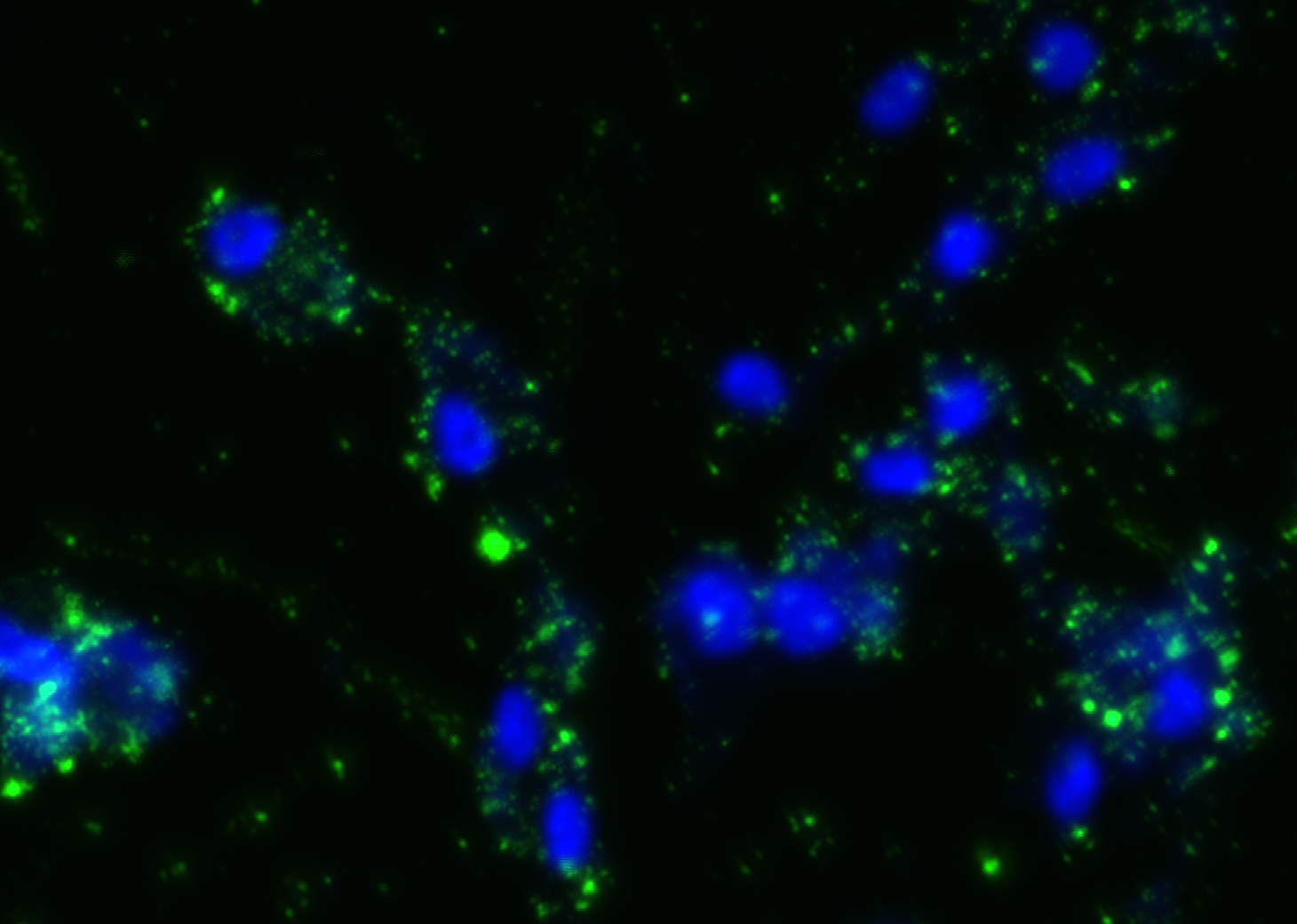

Supplement: S1 File — (ZIP) [file pone.0335890.s001.zip › Supporting Information1/Fig2/Fig2b/Exo-BSA@dBET6.tif]

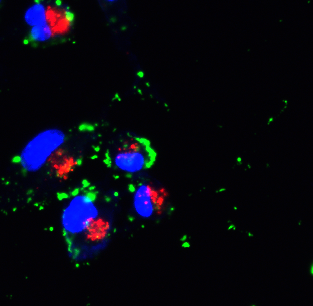

Supplement: S1 File — (ZIP) [file pone.0335890.s001.zip › Supporting Information1/Fig2/Fig2c/16h.tif]

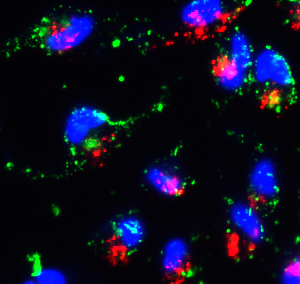

Supplement: S1 File — (ZIP) [file pone.0335890.s001.zip › Supporting Information1/Fig2/Fig2c/24h.tif]

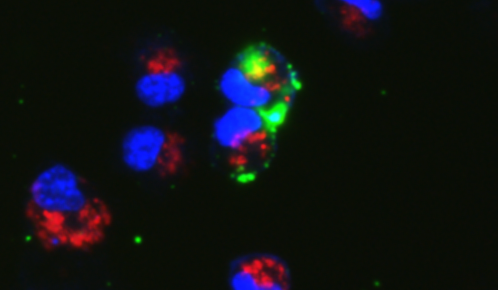

Supplement: S1 File — (ZIP) [file pone.0335890.s001.zip › Supporting Information1/Fig2/Fig2c/4h.tif]

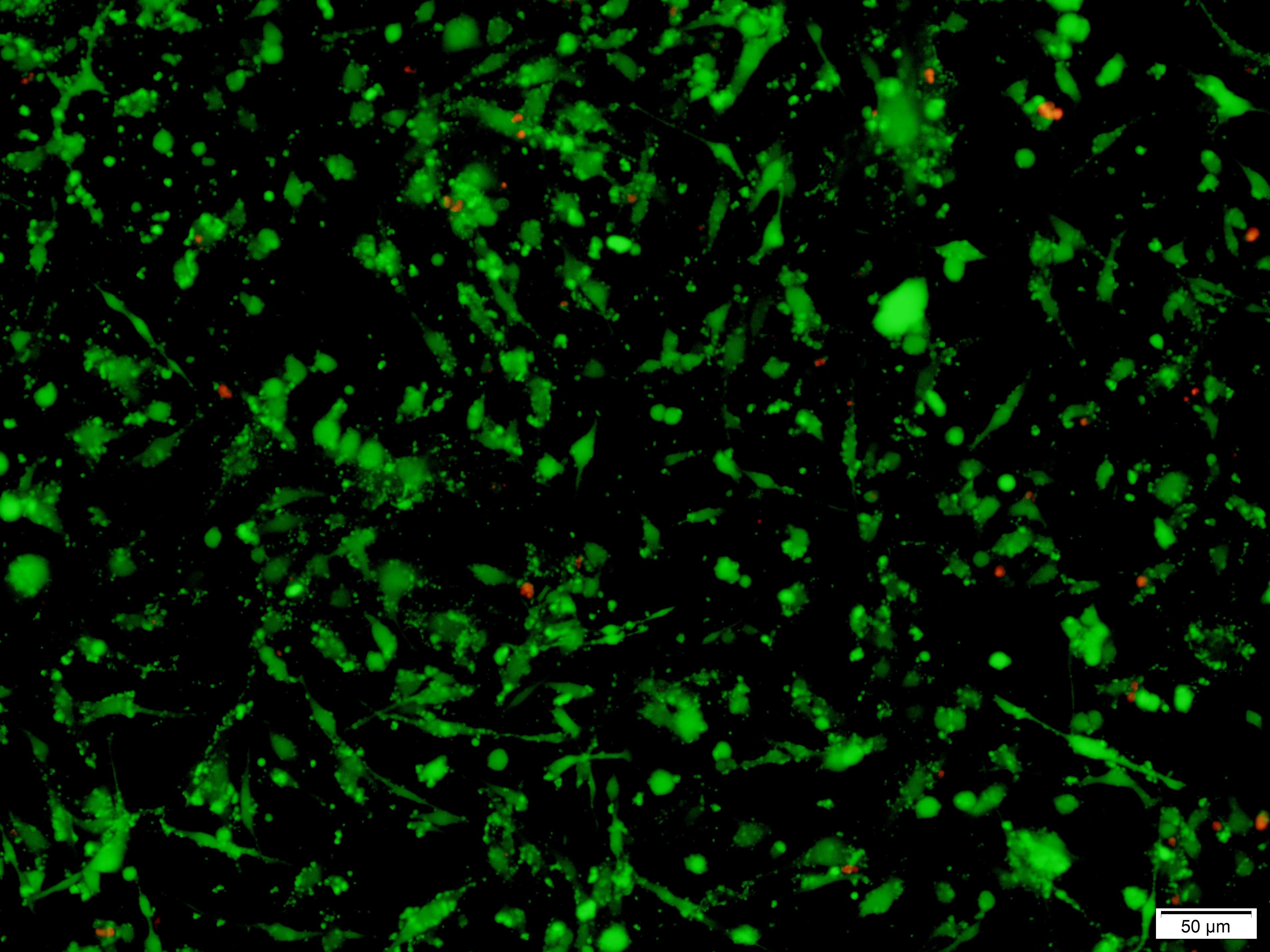

Supplement: S2 File — (ZIP) [file pone.0335890.s002.zip › Supporting Information2/Fig3/Fig3c/CTL/20904.png]

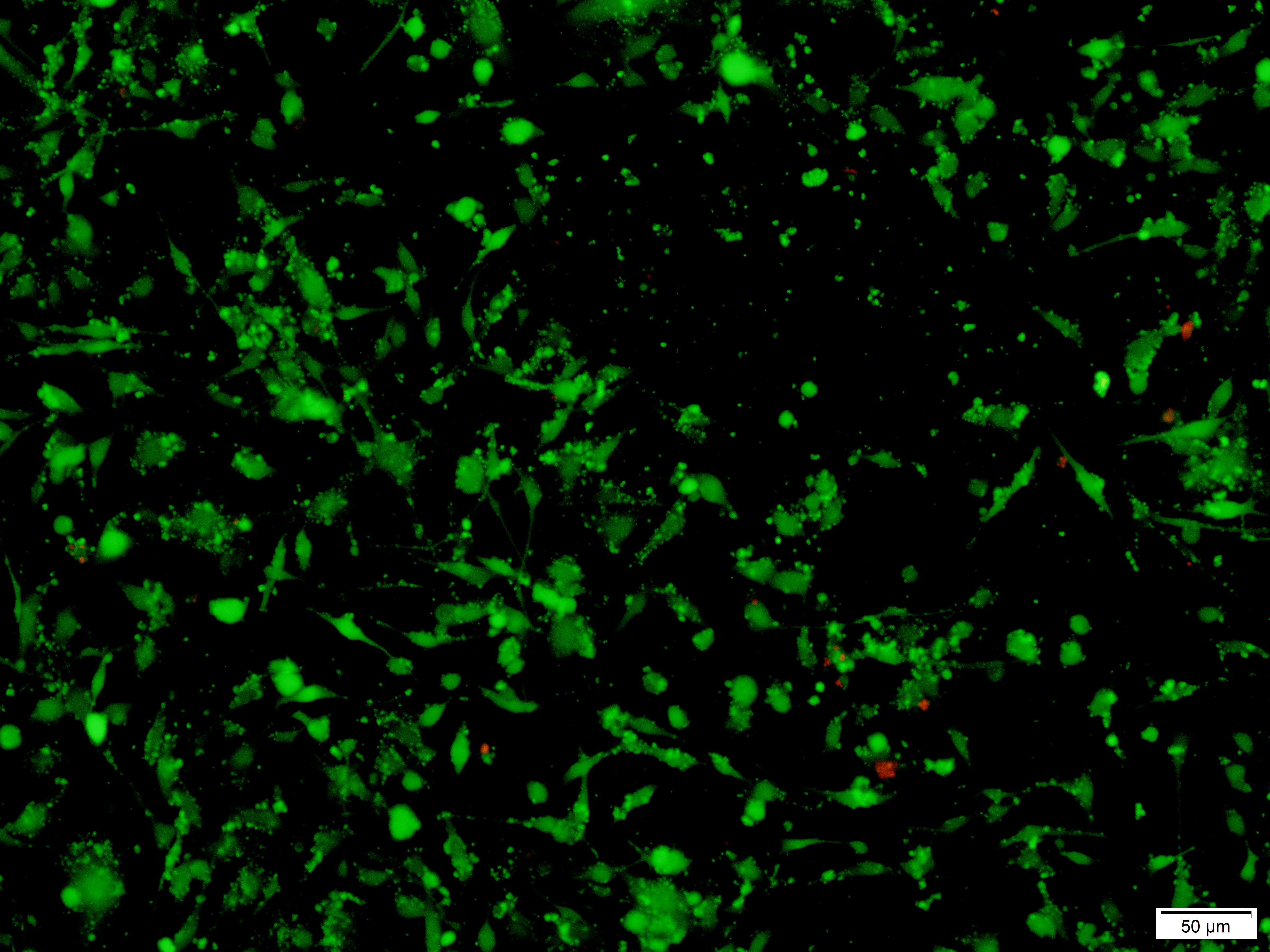

Supplement: S2 File — (ZIP) [file pone.0335890.s002.zip › Supporting Information2/Fig3/Fig3c/CTL/_20902.png]

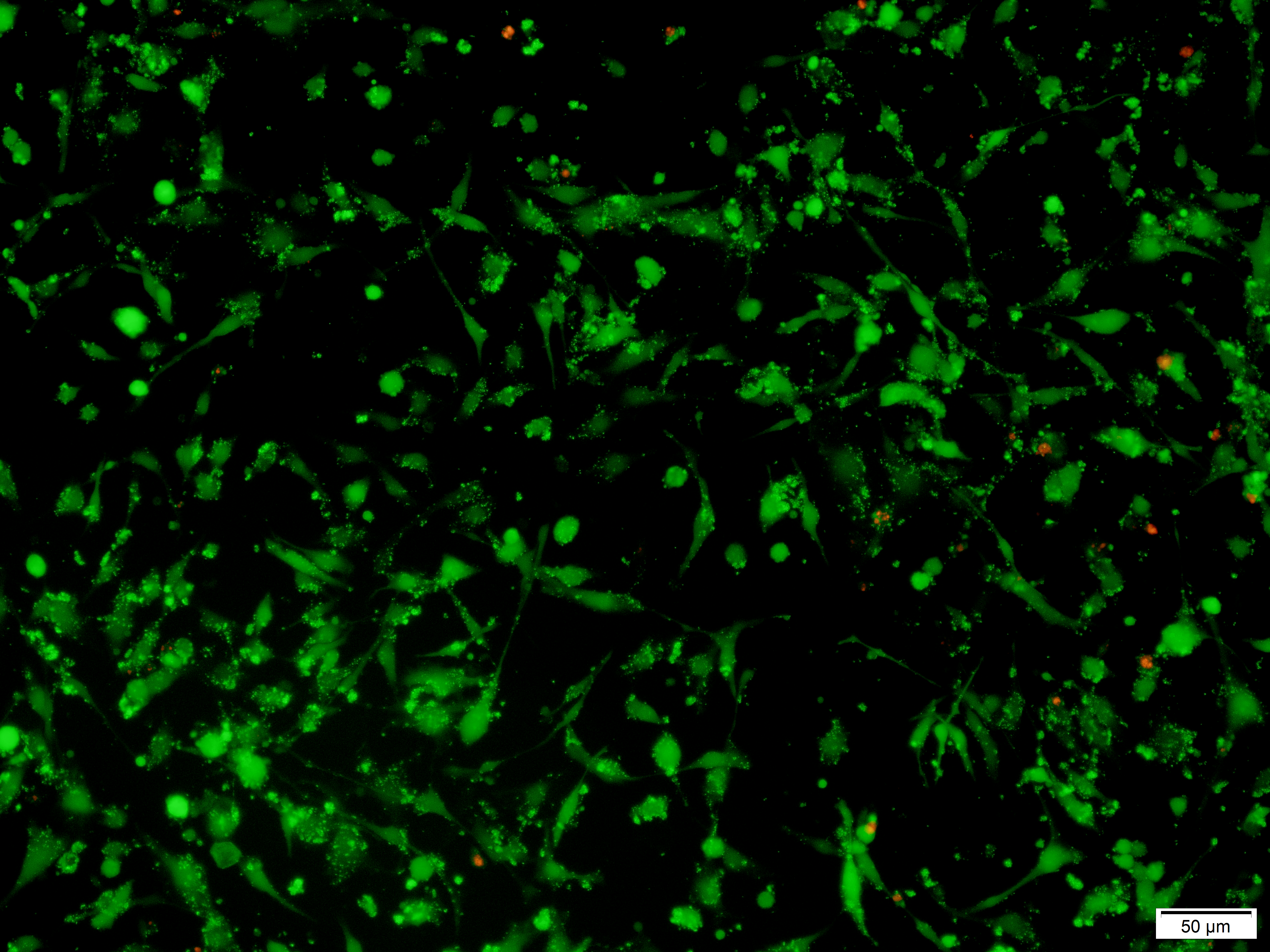

Supplement: S2 File — (ZIP) [file pone.0335890.s002.zip › Supporting Information2/Fig3/Fig3c/CTL/_20914.png]

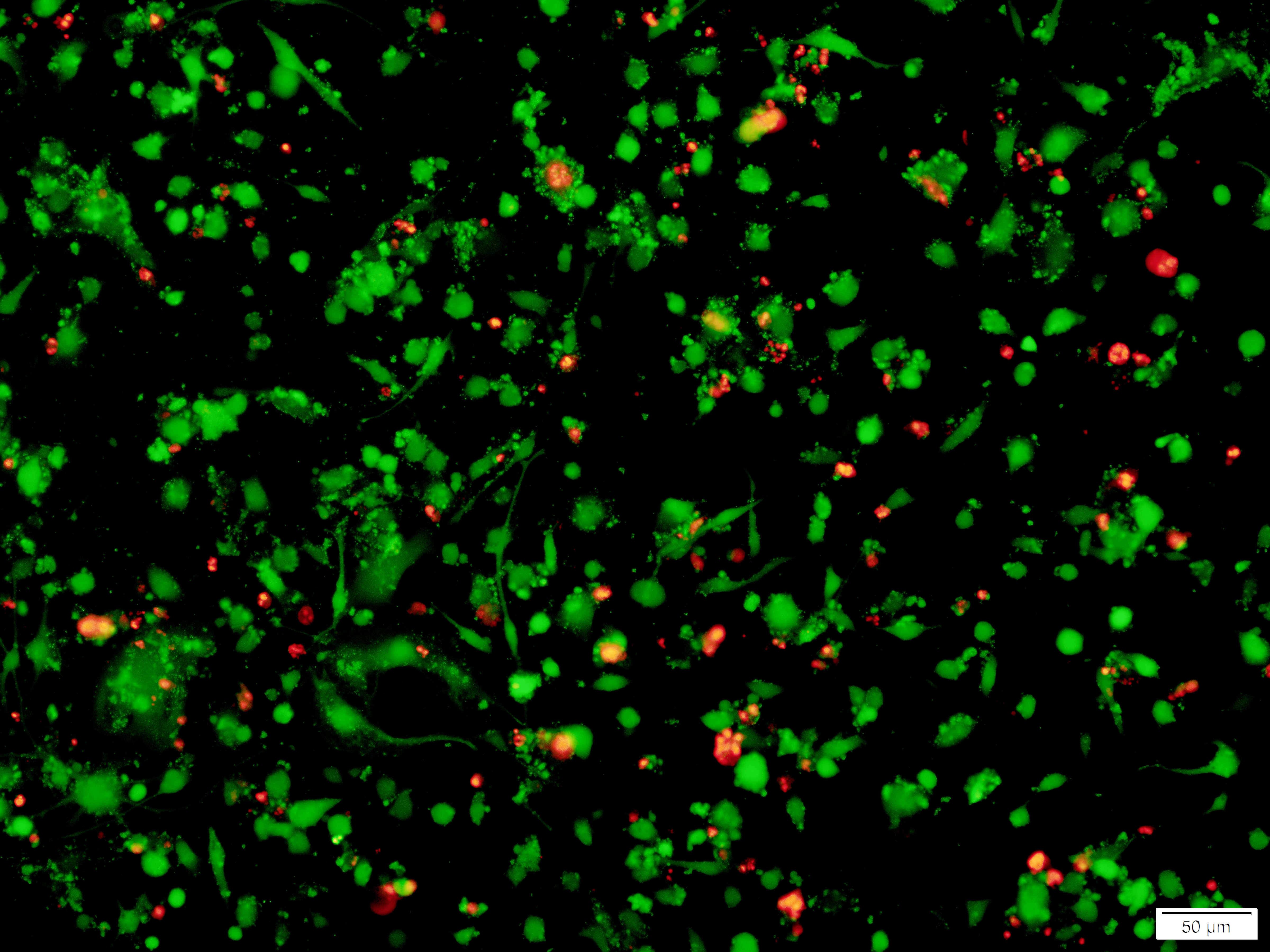

Supplement: S2 File — (ZIP) [file pone.0335890.s002.zip › Supporting Information2/Fig3/Fig3c/Exo-BSA@dBET6/20922-1.tif]

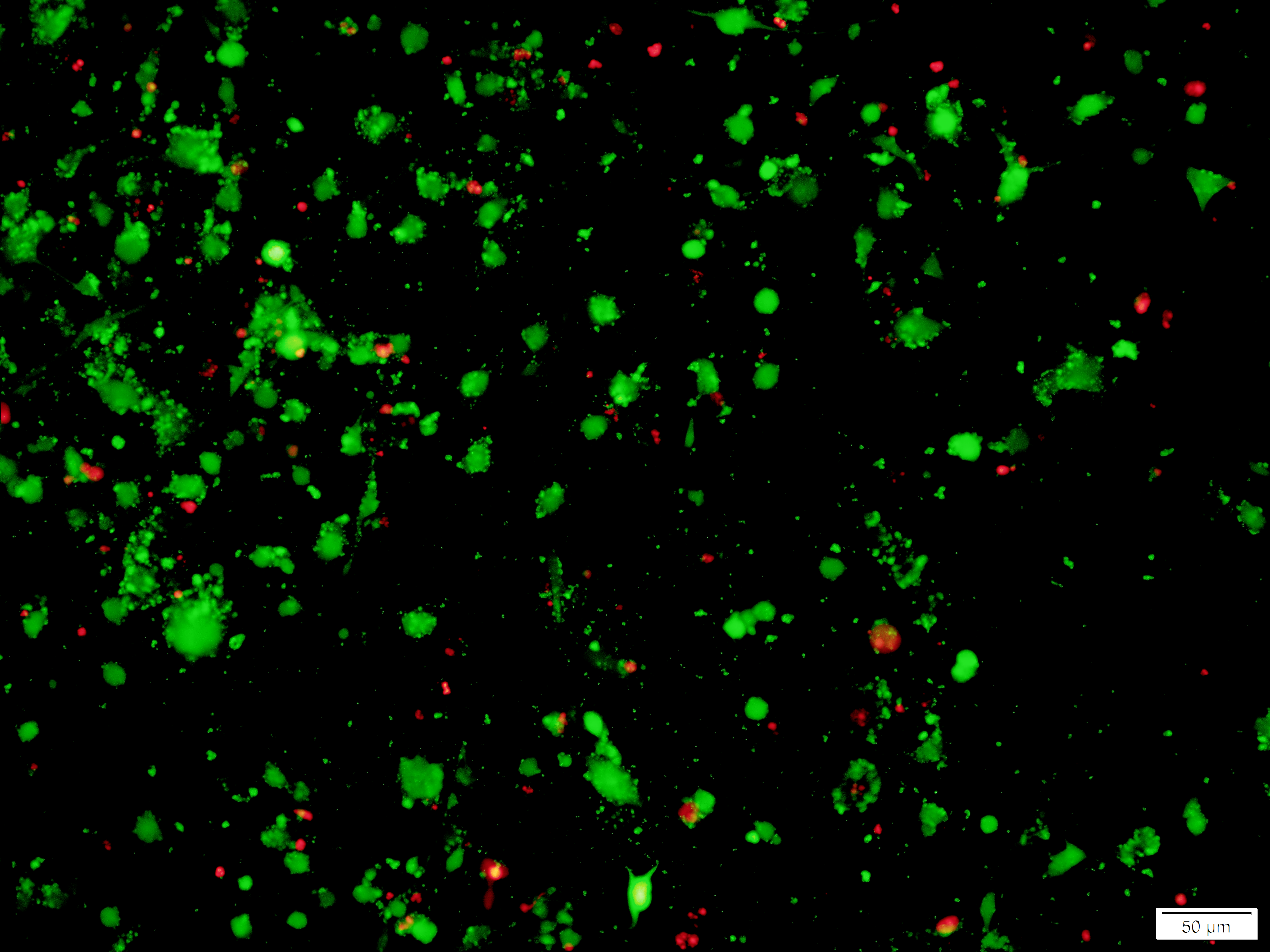

Supplement: S2 File — (ZIP) [file pone.0335890.s002.zip › Supporting Information2/Fig3/Fig3c/Exo-BSA@dBET6/20956-2.tif]

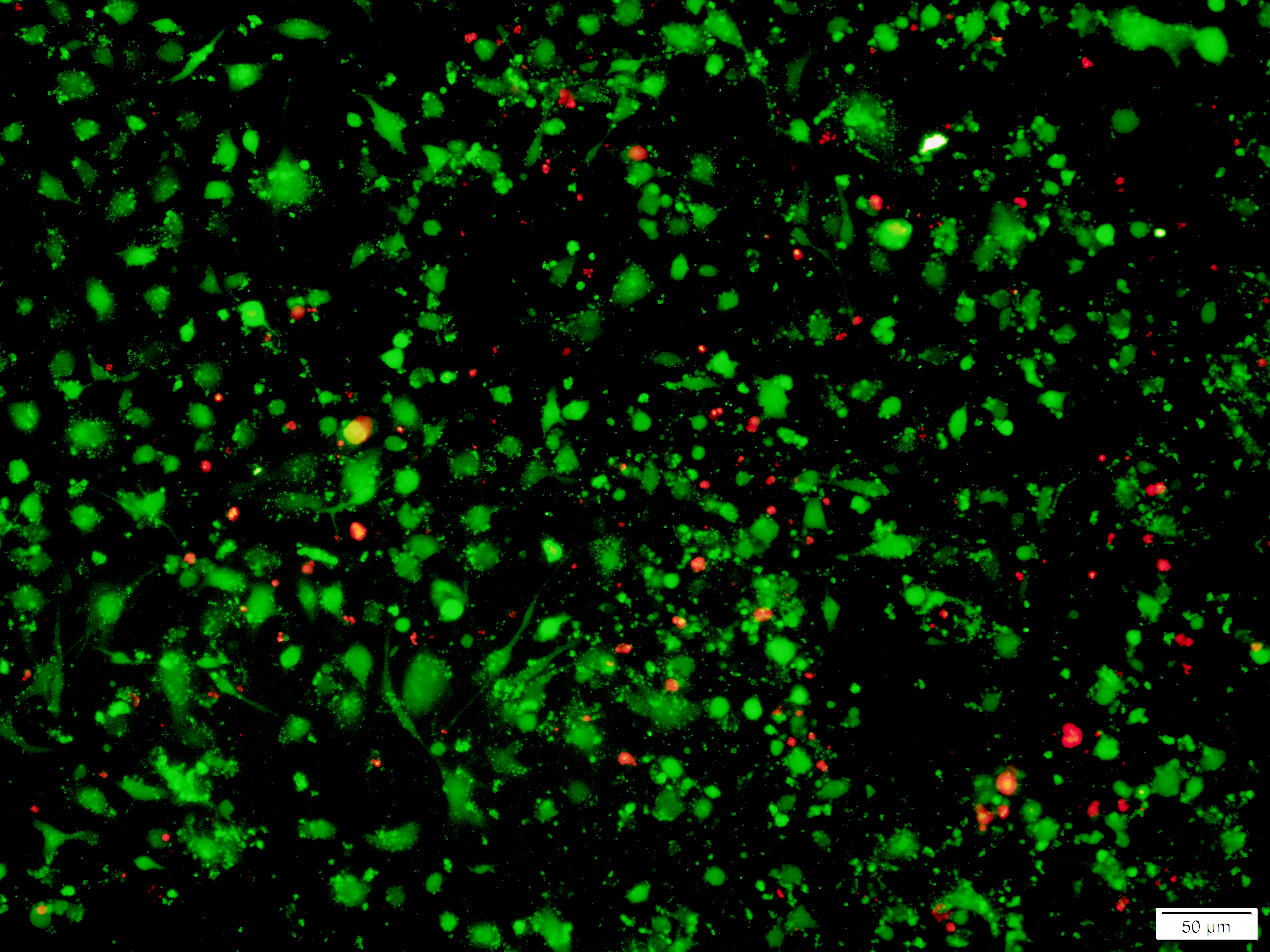

Supplement: S2 File — (ZIP) [file pone.0335890.s002.zip › Supporting Information2/Fig3/Fig3c/Exo-BSA@dBET6/20958-3.tif]

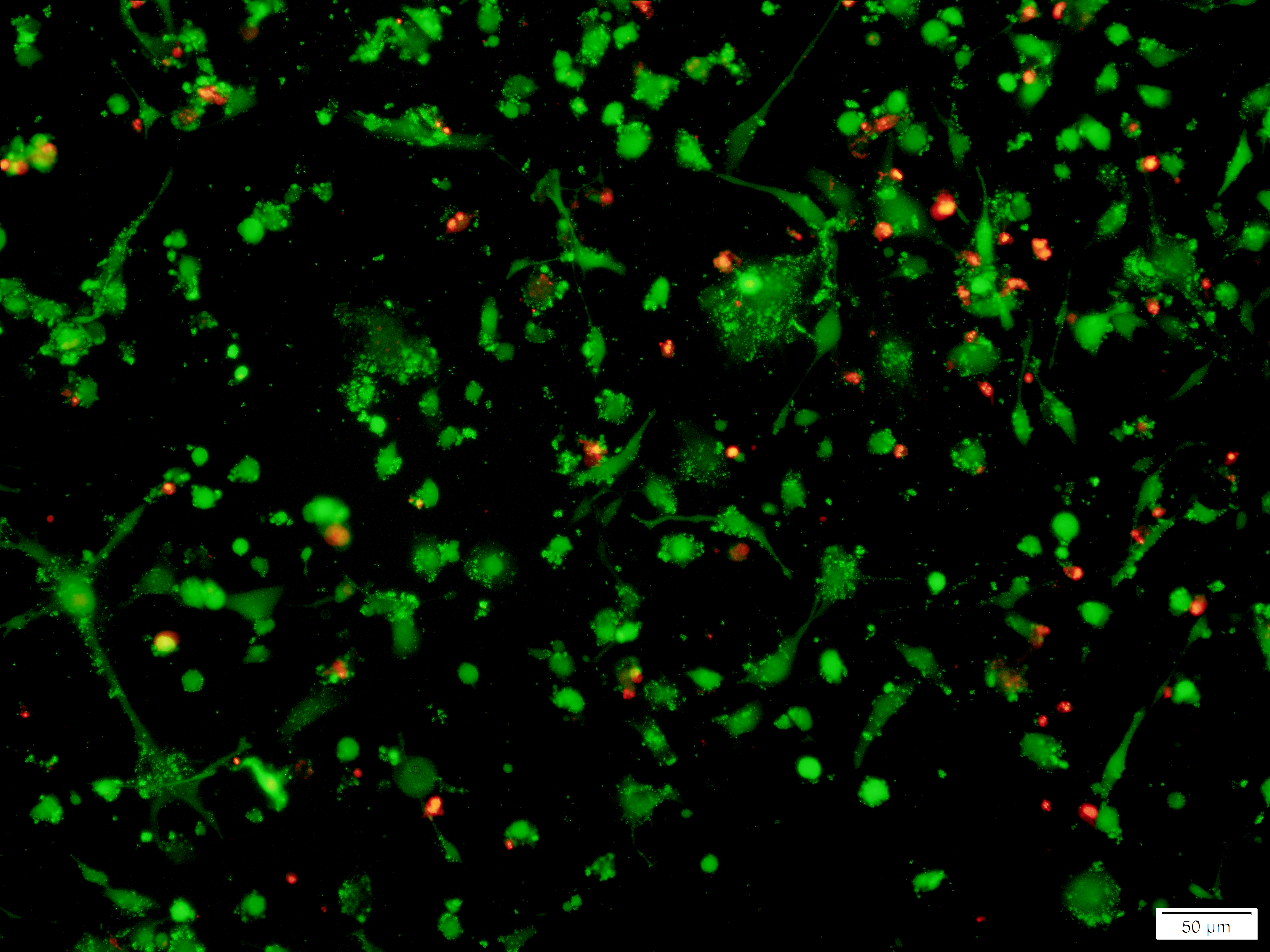

Supplement: S2 File — (ZIP) [file pone.0335890.s002.zip › Supporting Information2/Fig3/Fig3c/dBET6/20918-1.png]

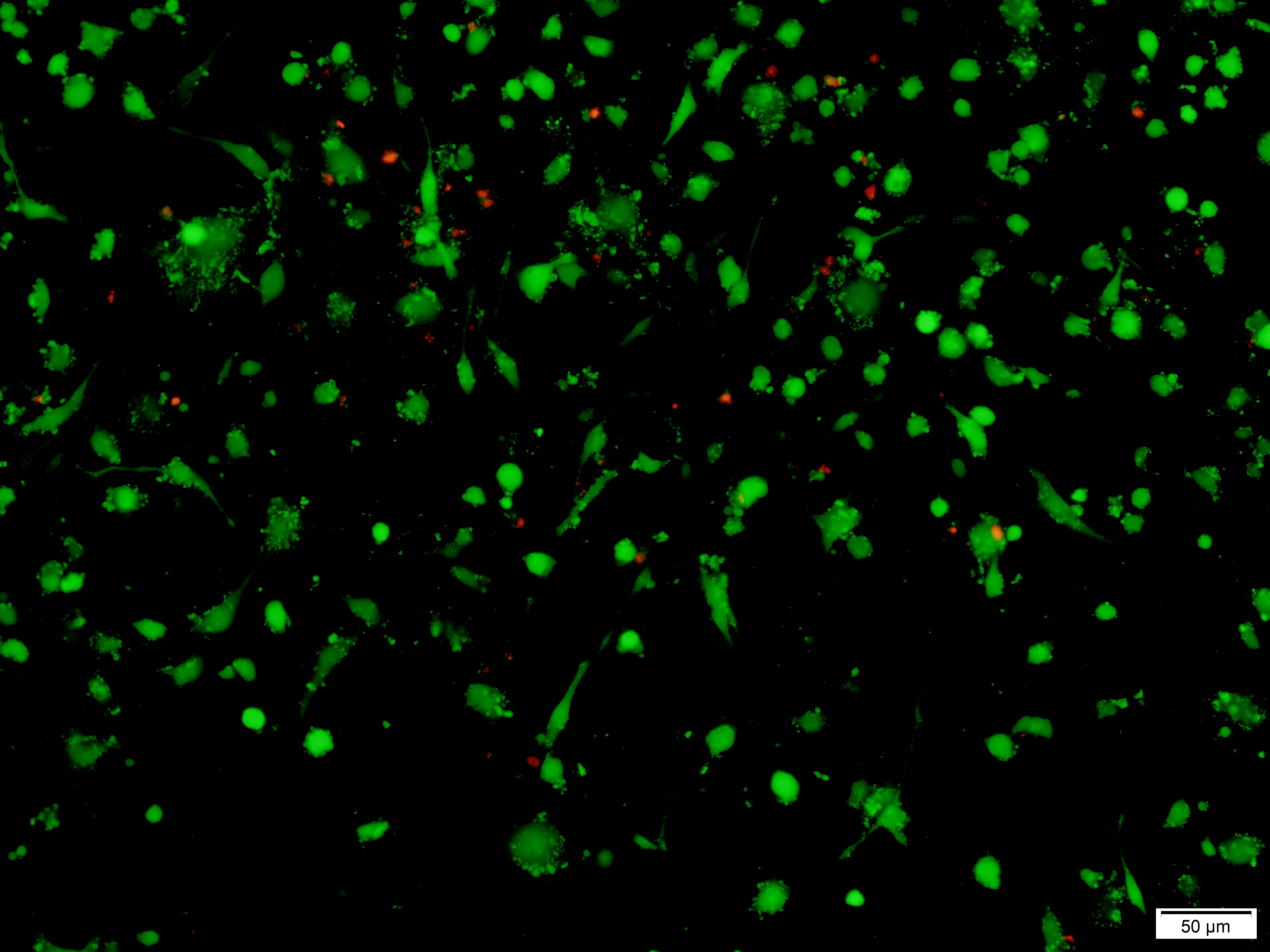

Supplement: S2 File — (ZIP) [file pone.0335890.s002.zip › Supporting Information2/Fig3/Fig3c/dBET6/20924-2.png]

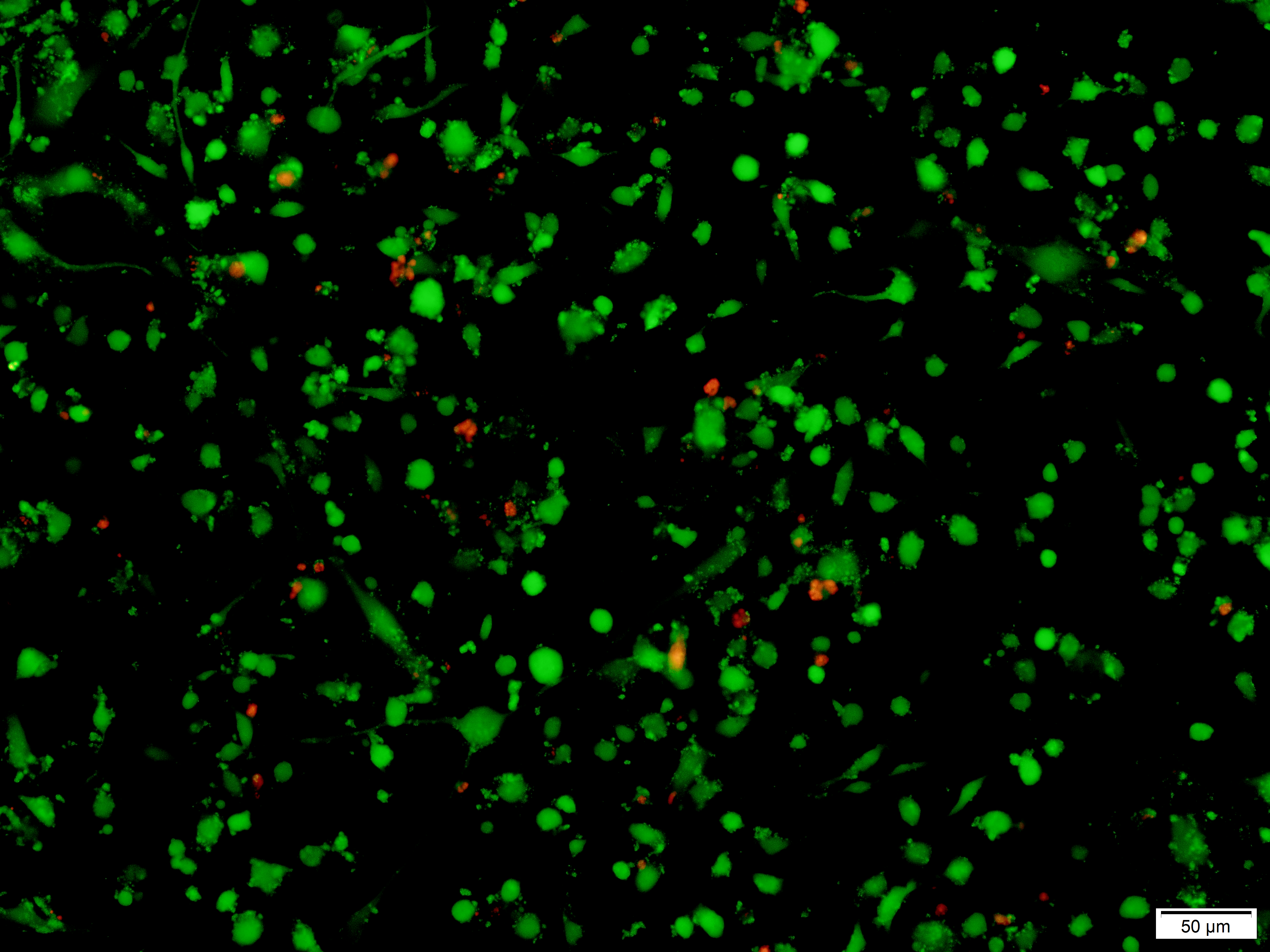

Supplement: S2 File — (ZIP) [file pone.0335890.s002.zip › Supporting Information2/Fig3/Fig3c/dBET6/20926-3.png]

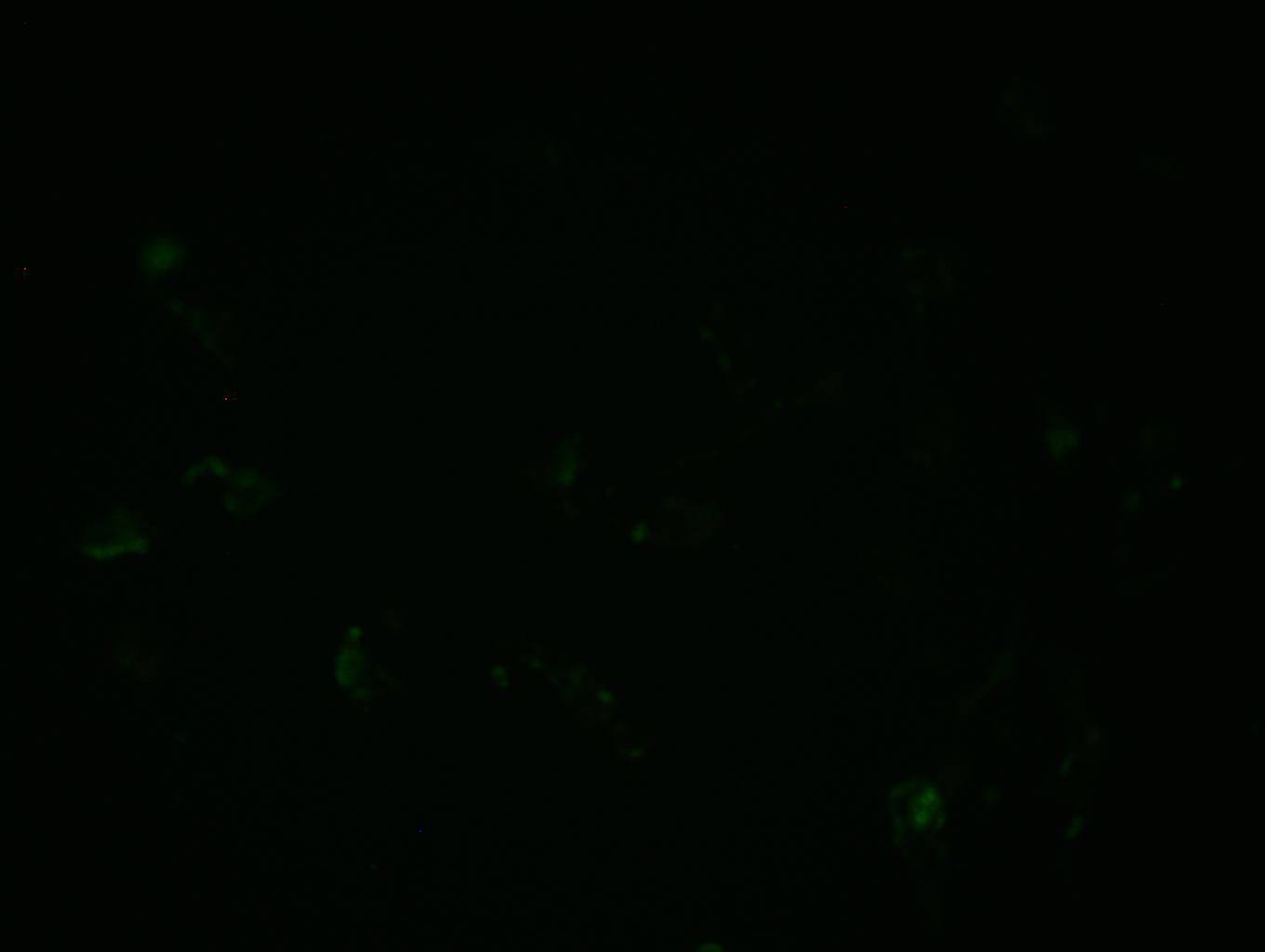

Supplement: S3 File — (ZIP) [file pone.0335890.s003.zip › Supporting Information3/Fig3/Fig3d/CTL/1..png]

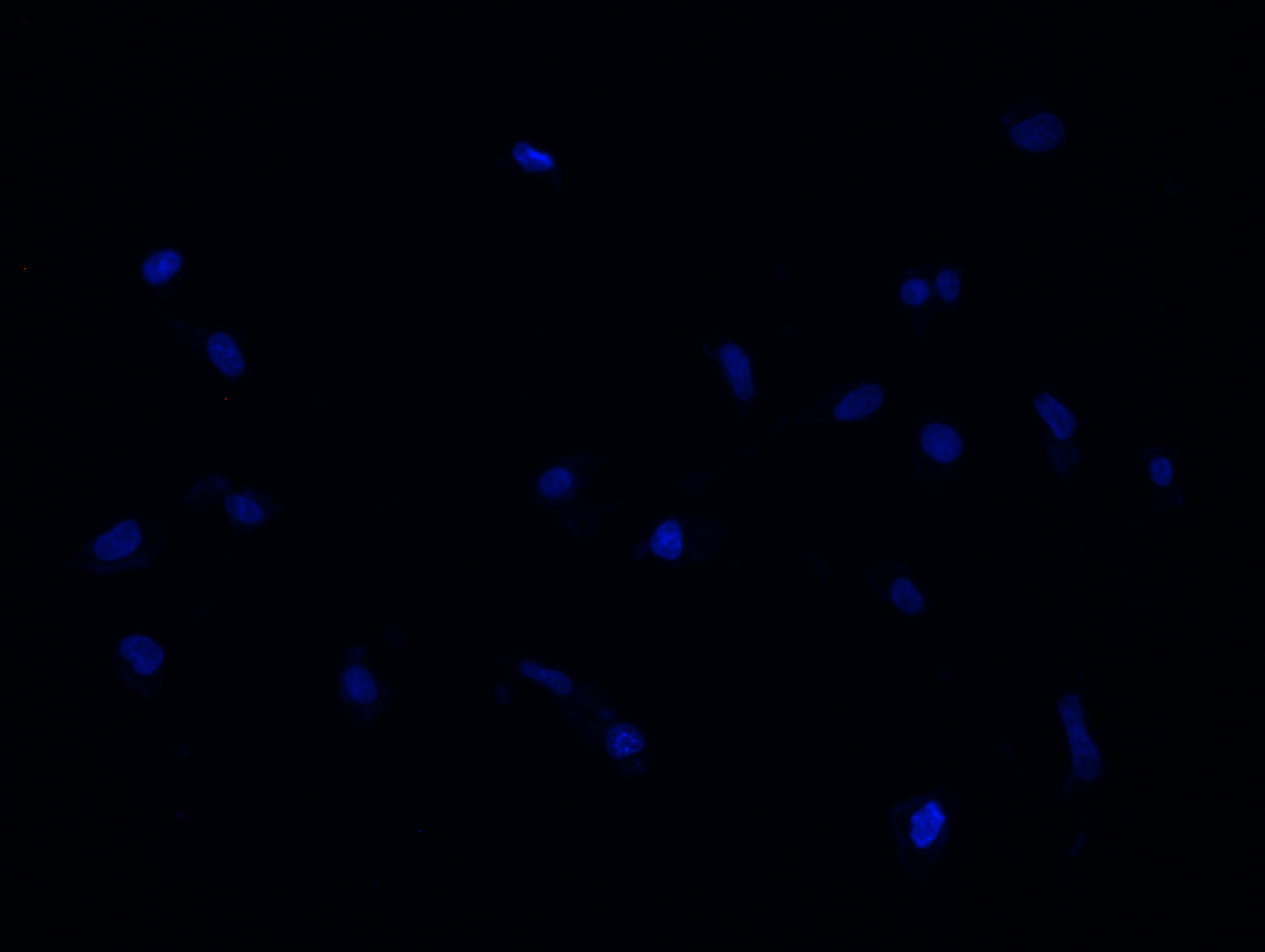

Supplement: S3 File — (ZIP) [file pone.0335890.s003.zip › Supporting Information3/Fig3/Fig3d/CTL/1.png]

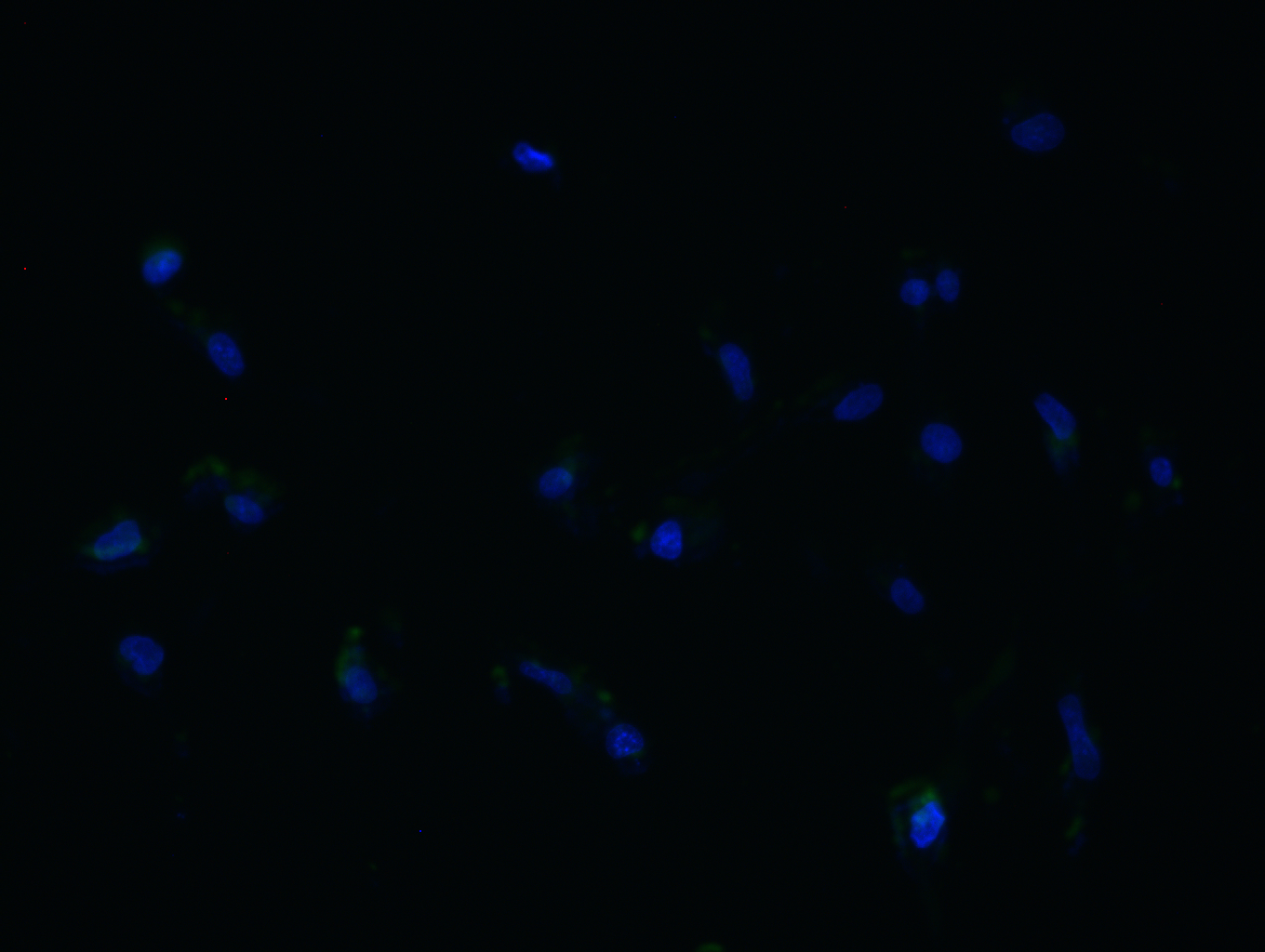

Supplement: S3 File — (ZIP) [file pone.0335890.s003.zip › Supporting Information3/Fig3/Fig3d/CTL/1OK.png]

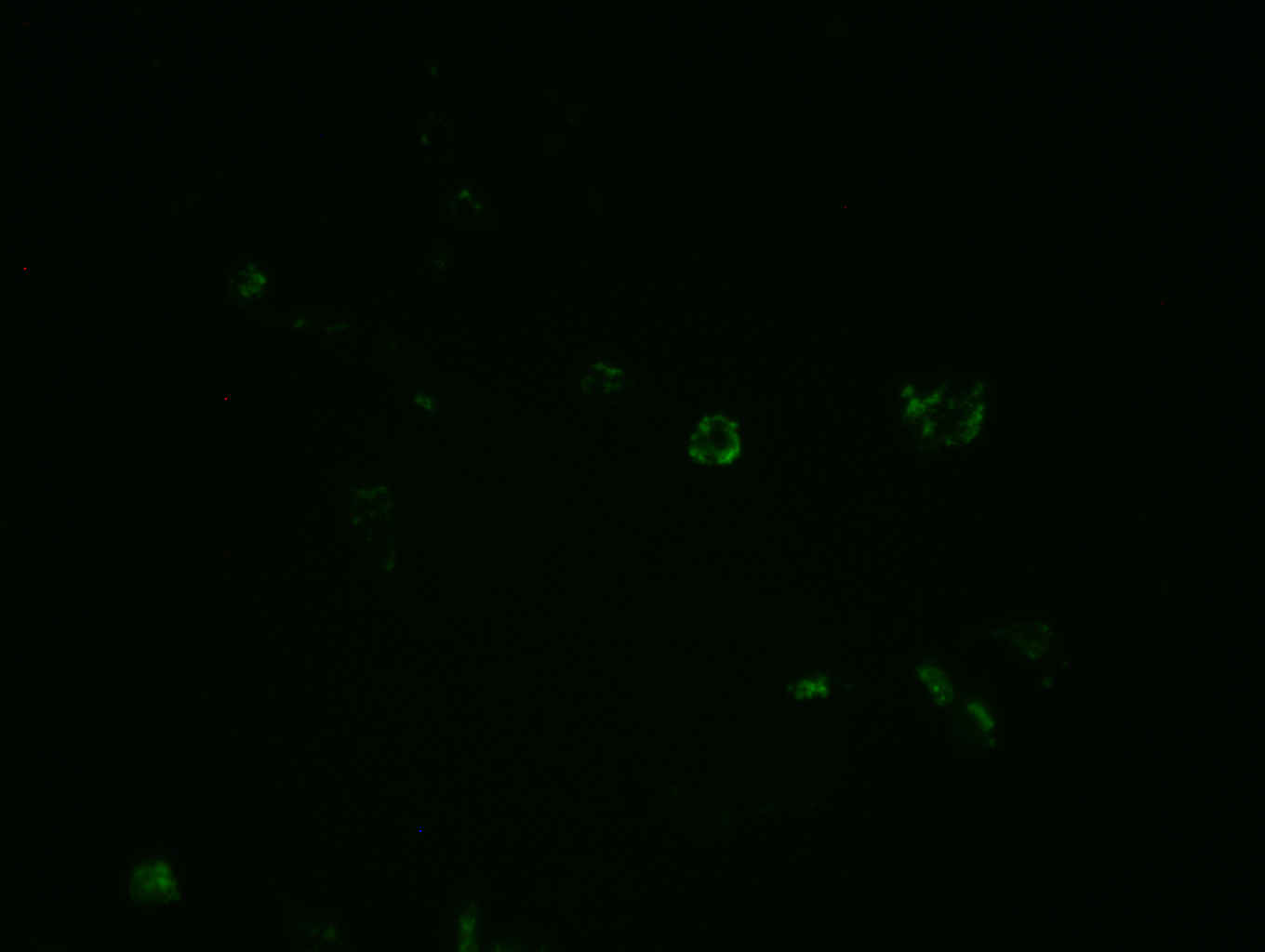

Supplement: S3 File — (ZIP) [file pone.0335890.s003.zip › Supporting Information3/Fig3/Fig3d/CTL/2..png]

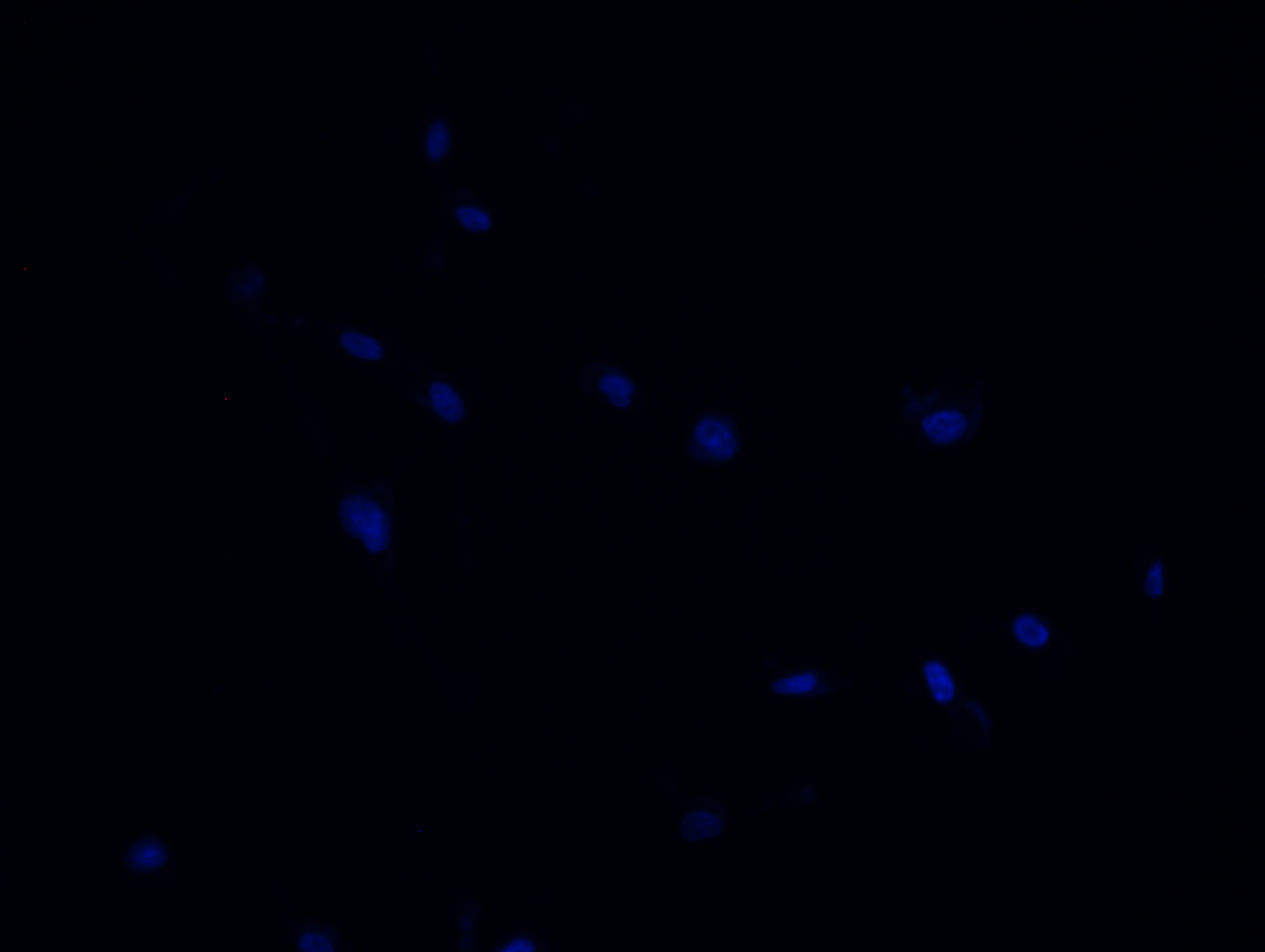

Supplement: S3 File — (ZIP) [file pone.0335890.s003.zip › Supporting Information3/Fig3/Fig3d/CTL/2.png]

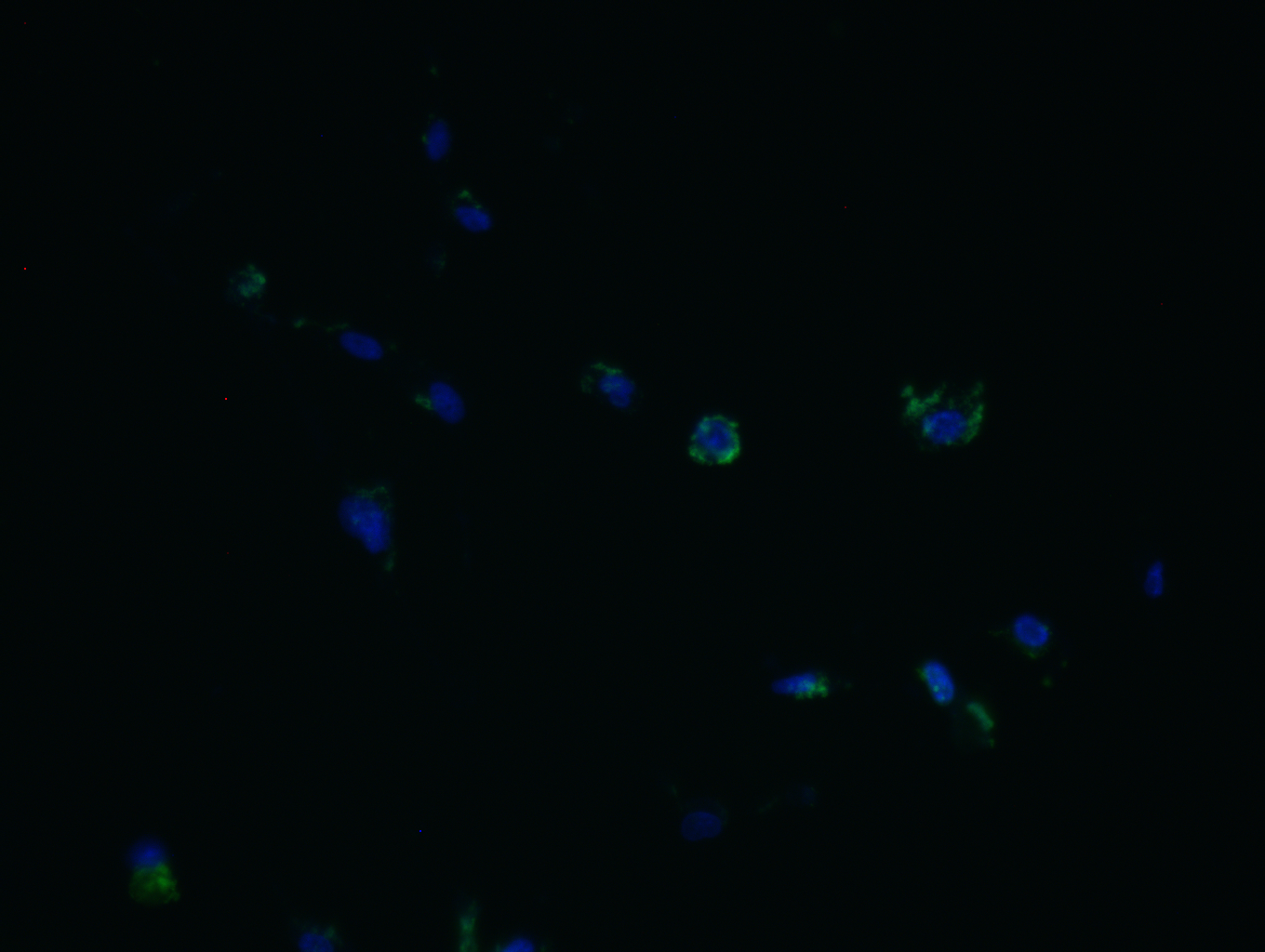

Supplement: S3 File — (ZIP) [file pone.0335890.s003.zip › Supporting Information3/Fig3/Fig3d/CTL/2OK.png]

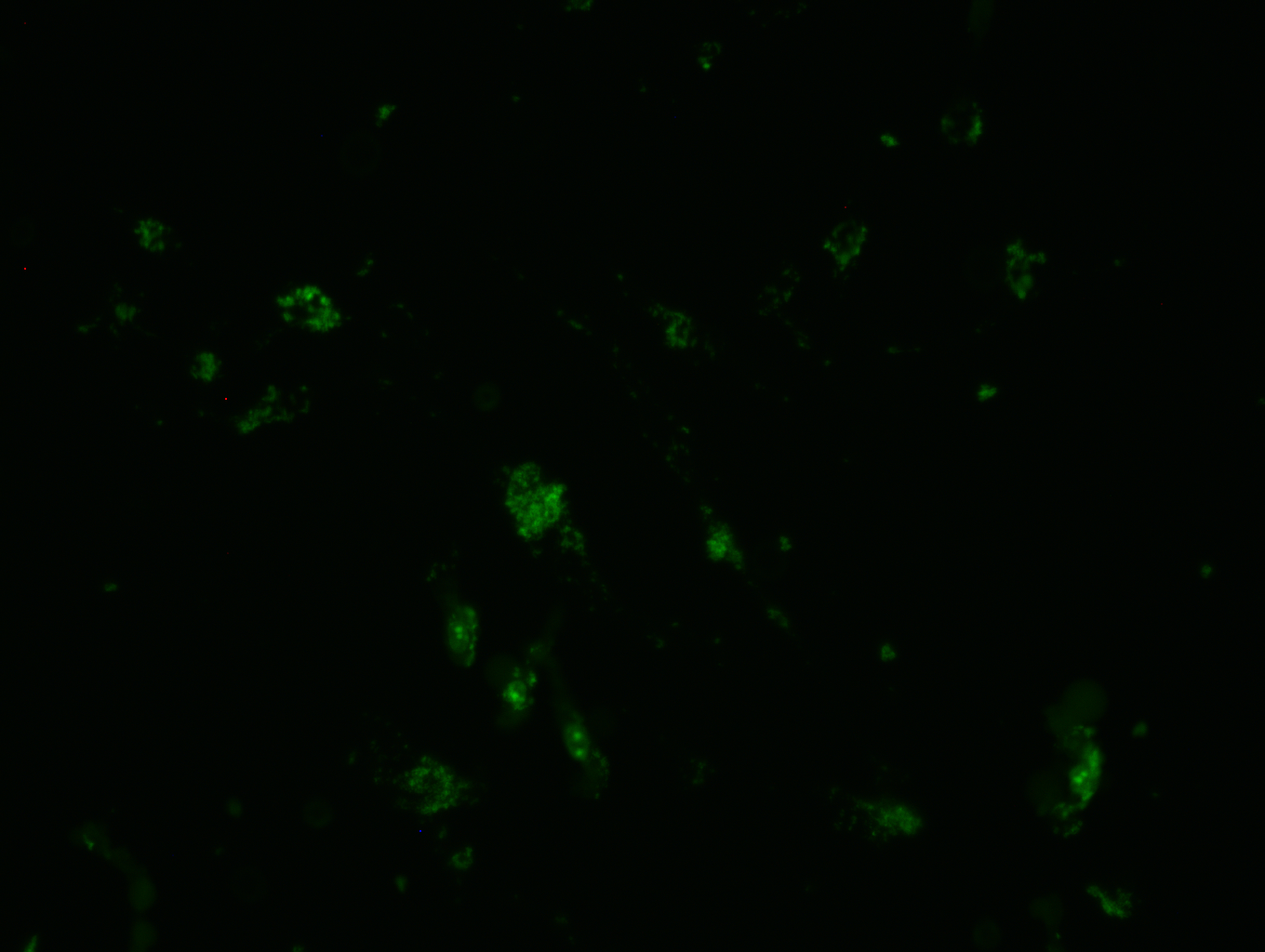

Supplement: S3 File — (ZIP) [file pone.0335890.s003.zip › Supporting Information3/Fig3/Fig3d/dbet6/1..png]

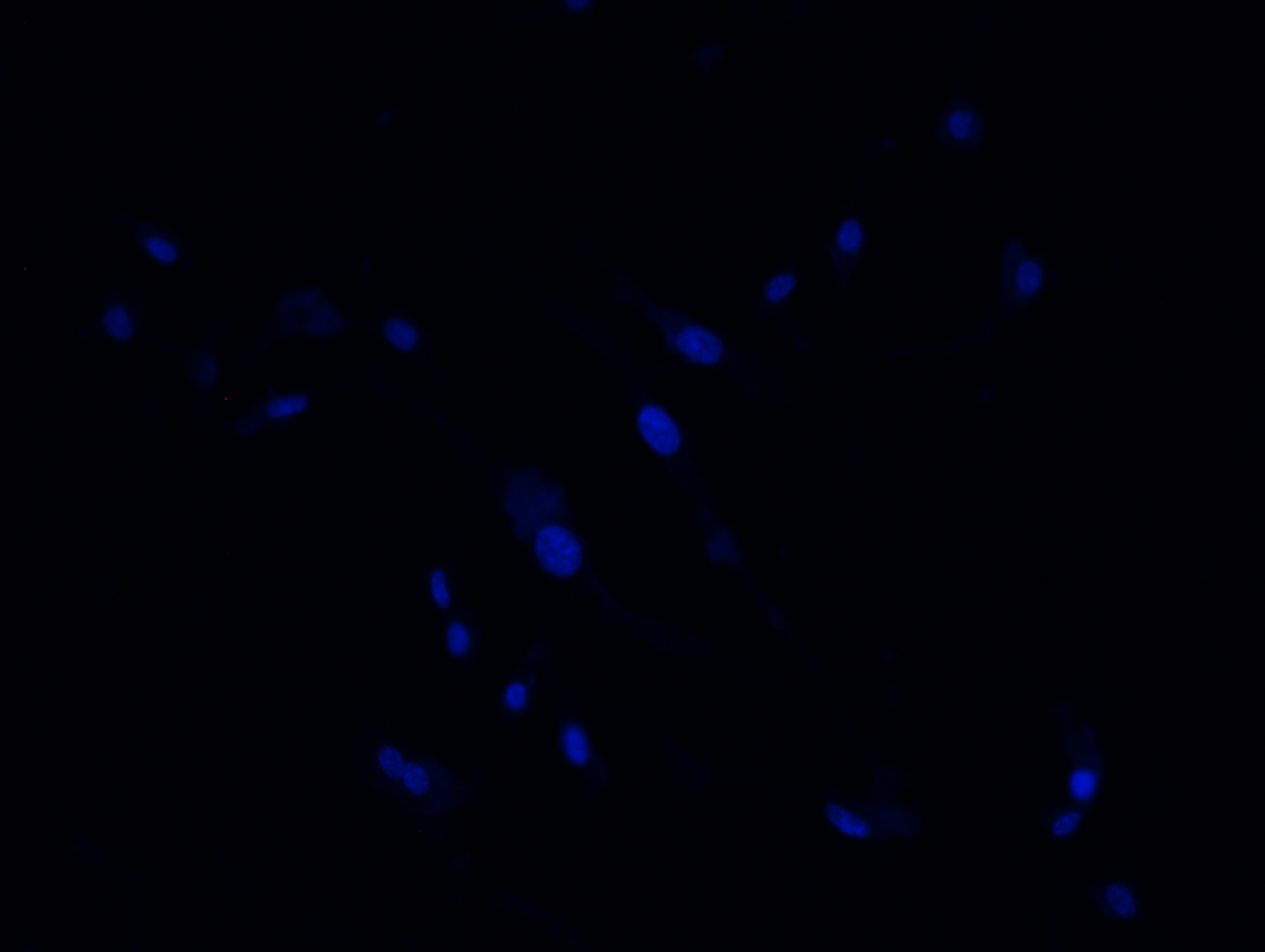

Supplement: S3 File — (ZIP) [file pone.0335890.s003.zip › Supporting Information3/Fig3/Fig3d/dbet6/1.png]

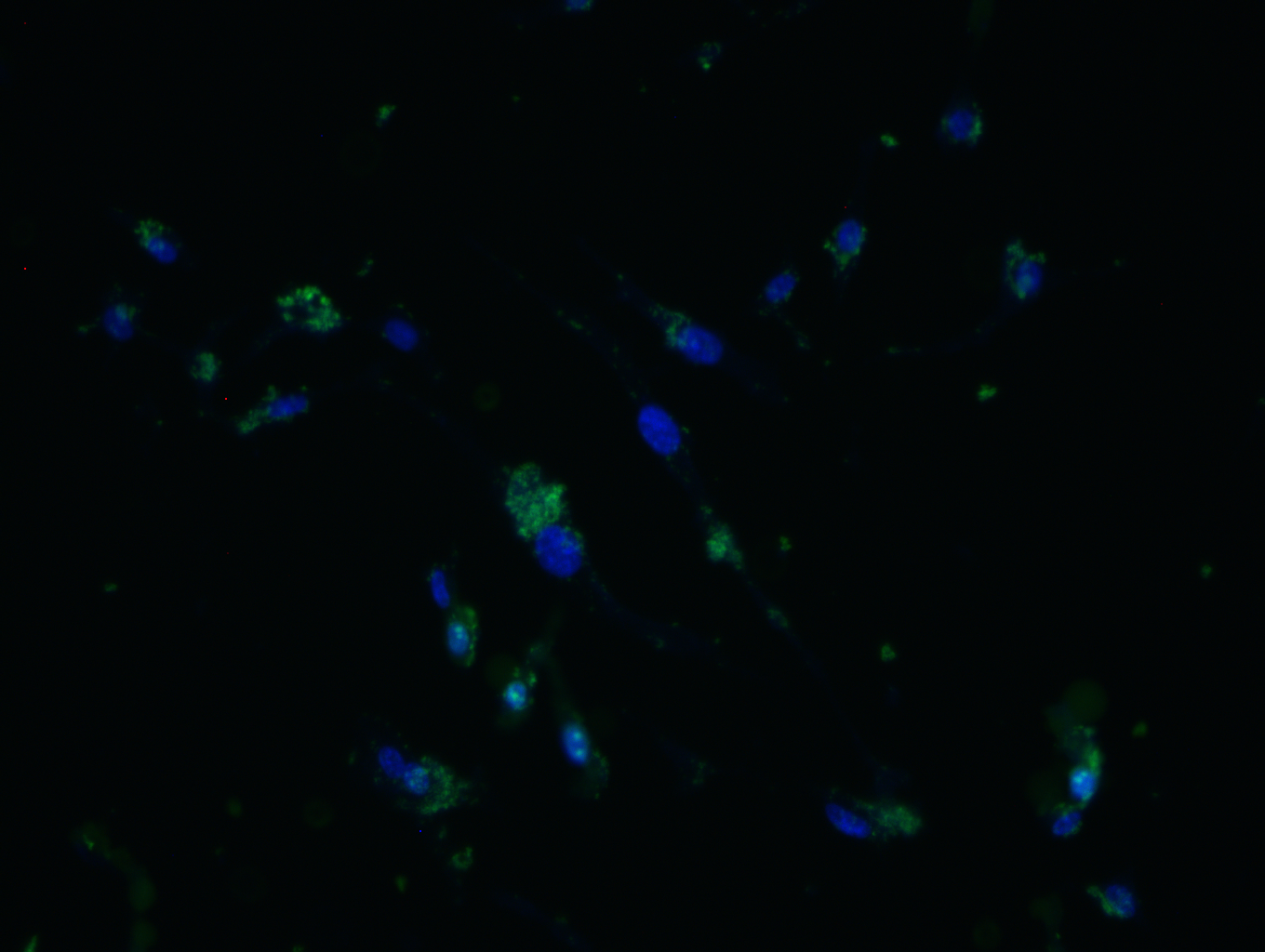

Supplement: S3 File — (ZIP) [file pone.0335890.s003.zip › Supporting Information3/Fig3/Fig3d/dbet6/1OK.png]

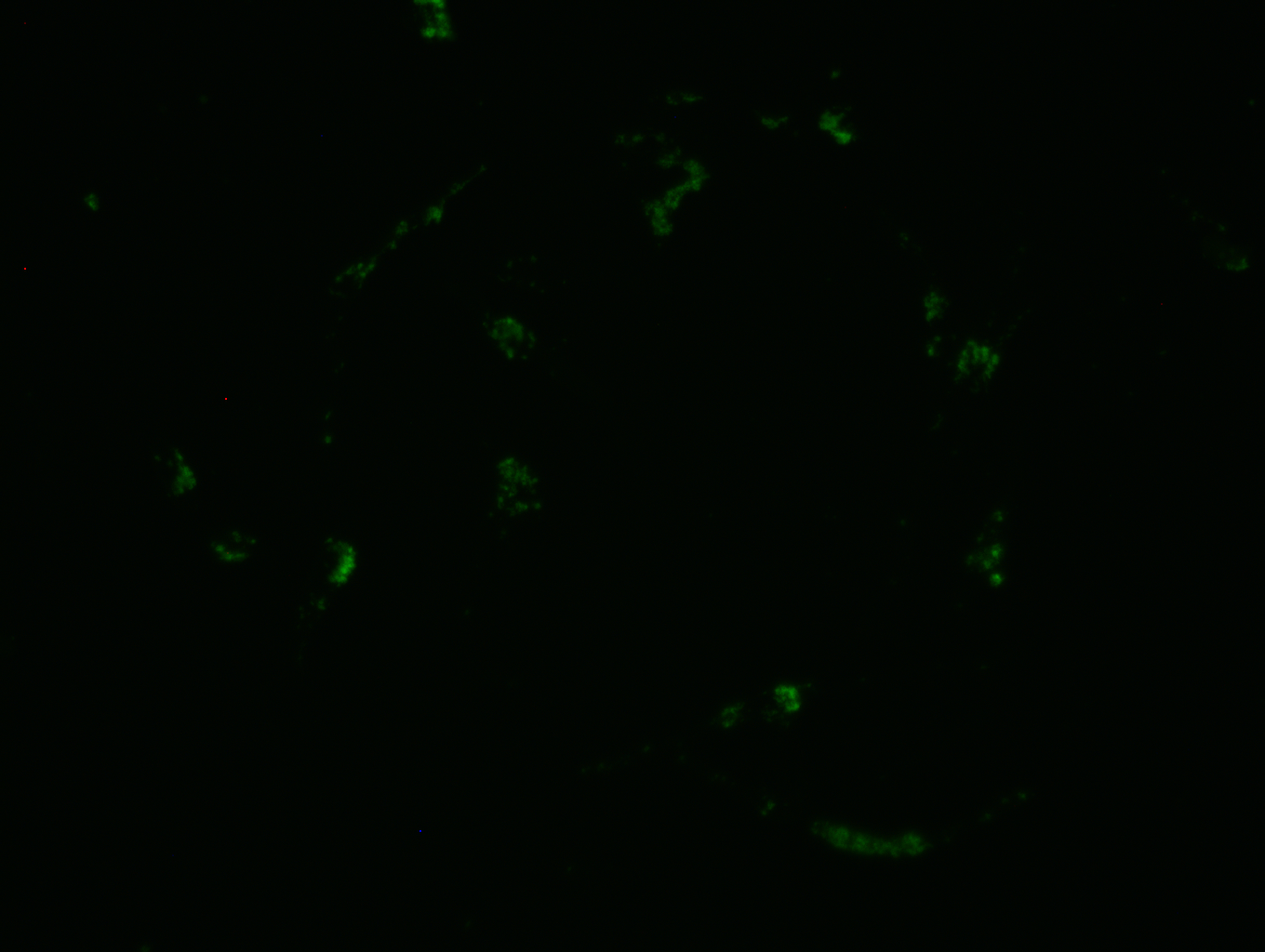

Supplement: S3 File — (ZIP) [file pone.0335890.s003.zip › Supporting Information3/Fig3/Fig3d/dbet6/2..png]

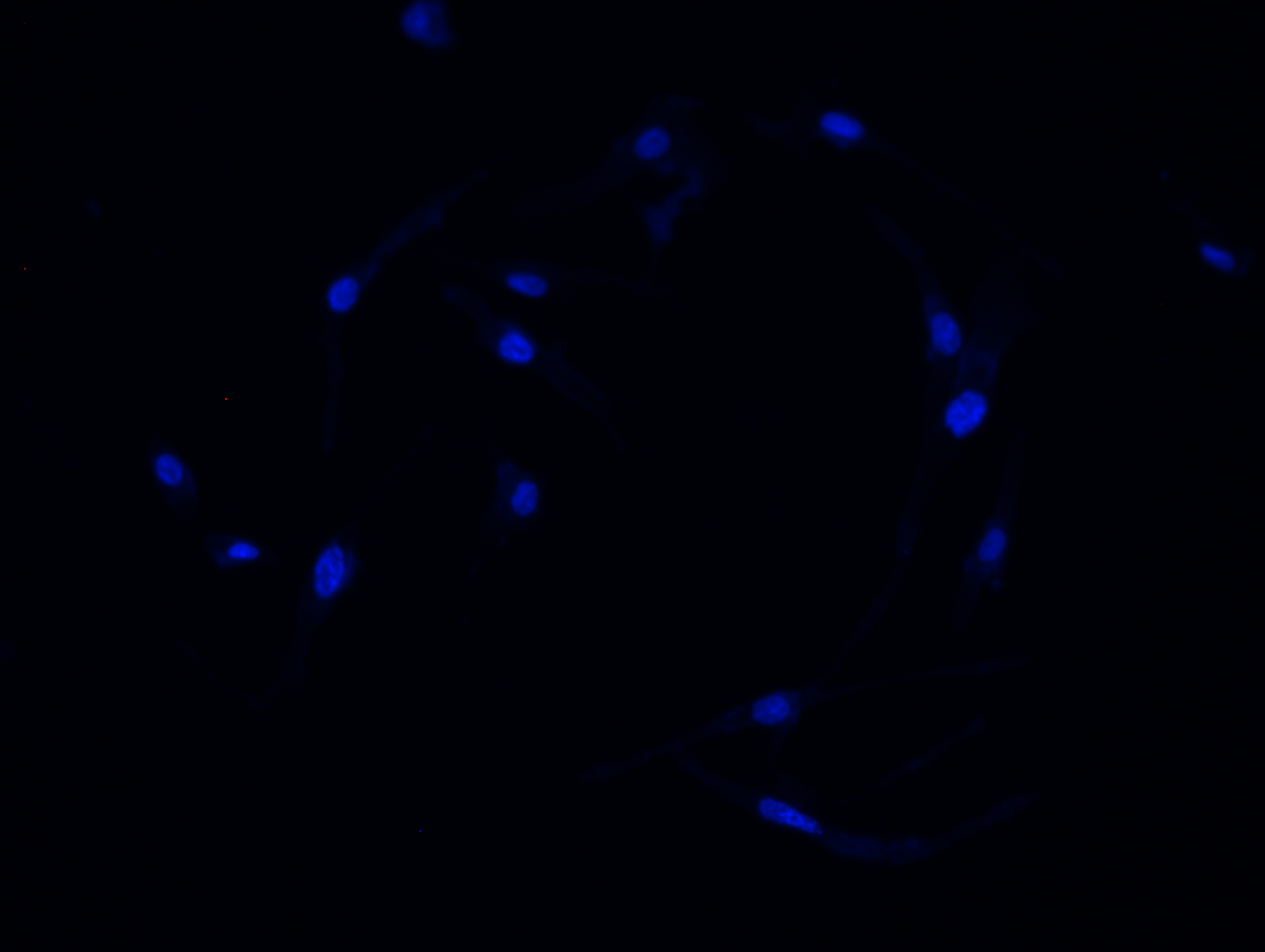

Supplement: S3 File — (ZIP) [file pone.0335890.s003.zip › Supporting Information3/Fig3/Fig3d/dbet6/2.png]

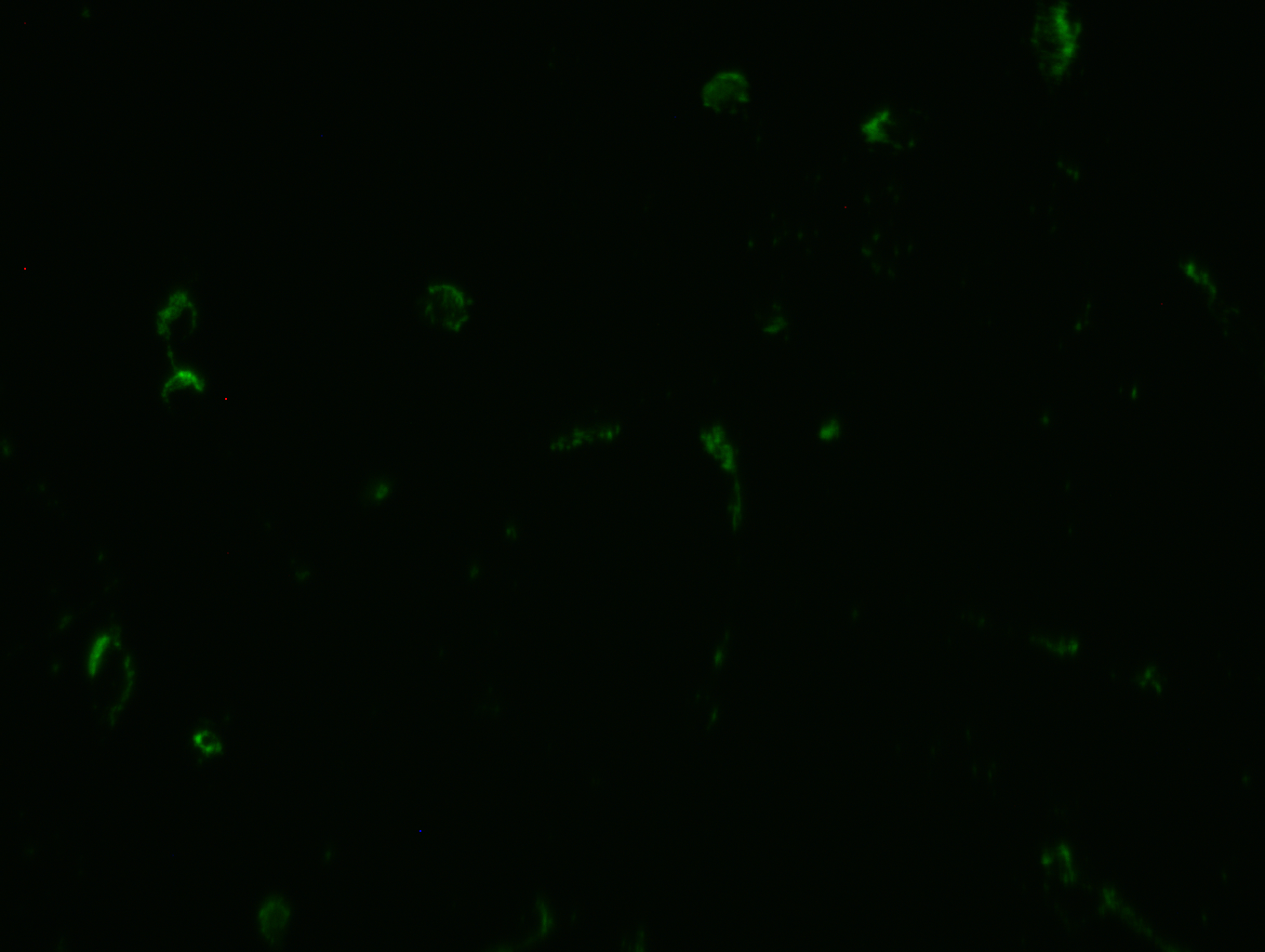

Supplement: S3 File — (ZIP) [file pone.0335890.s003.zip › Supporting Information3/Fig3/Fig3d/dbet6/3..png]

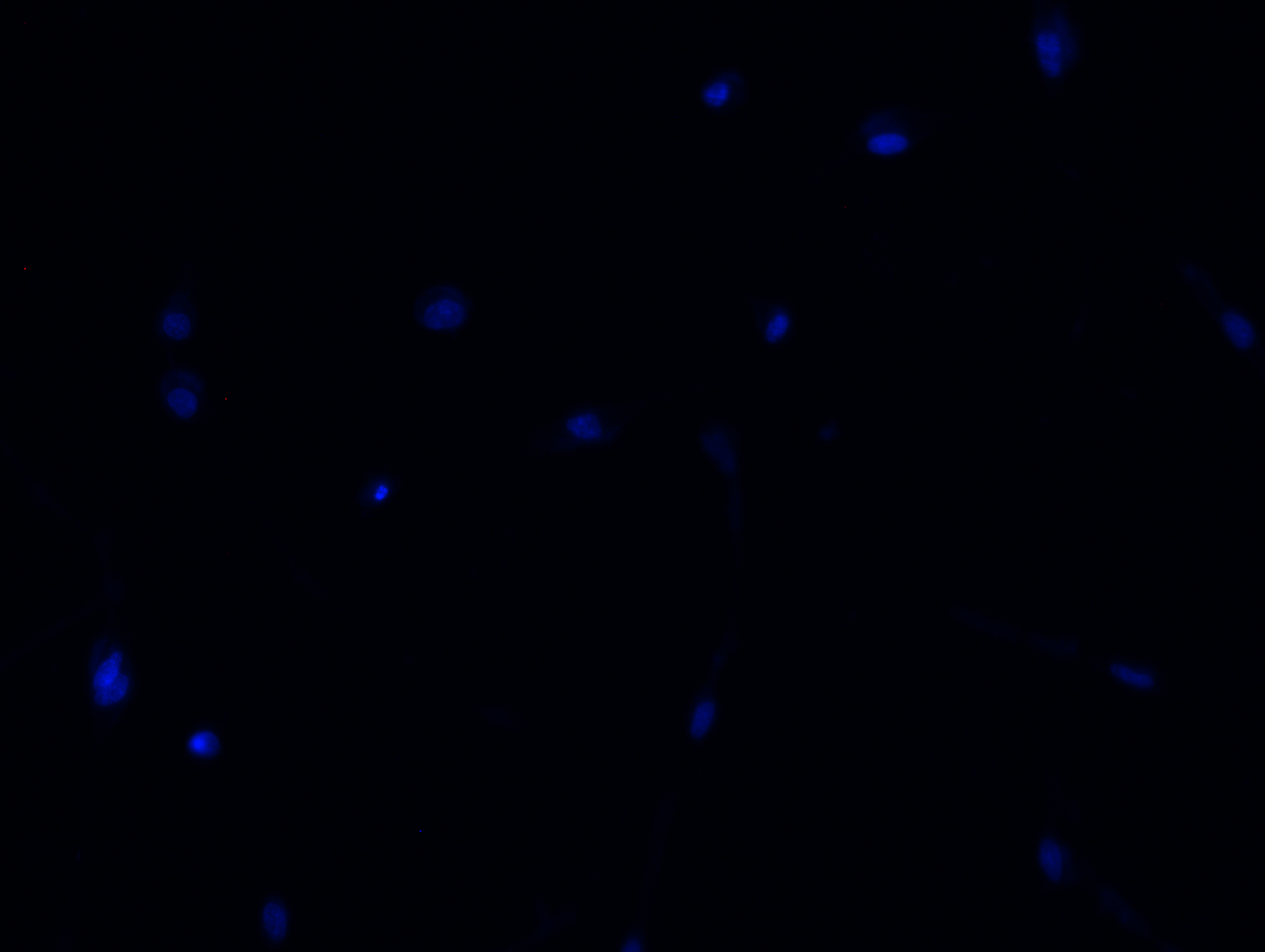

Supplement: S3 File — (ZIP) [file pone.0335890.s003.zip › Supporting Information3/Fig3/Fig3d/dbet6/3.png]

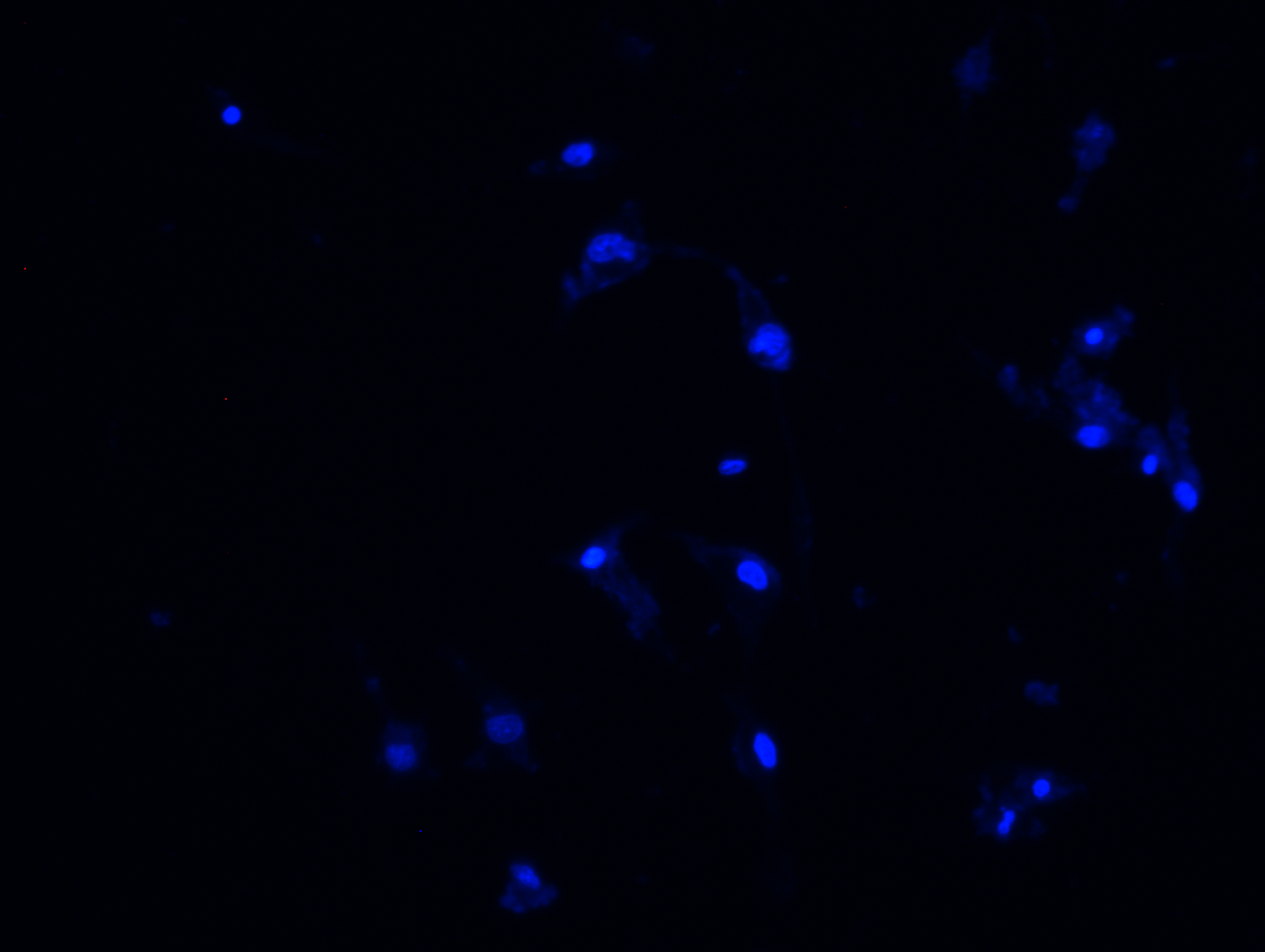

Supplement: S3 File — (ZIP) [file pone.0335890.s003.zip › Supporting Information3/Fig3/Fig3d/exo-bsa/1..png]

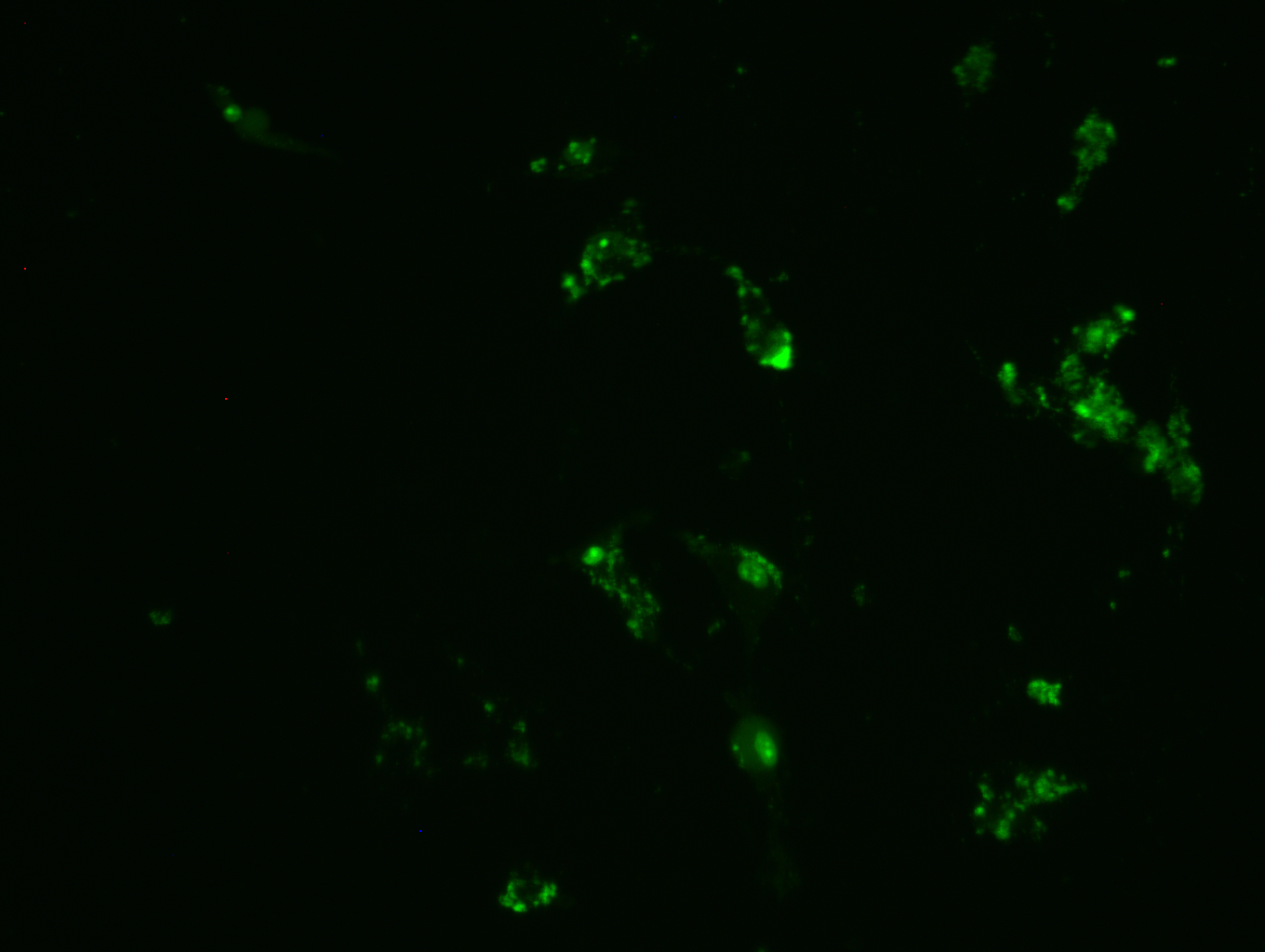

Supplement: S3 File — (ZIP) [file pone.0335890.s003.zip › Supporting Information3/Fig3/Fig3d/exo-bsa/1.png]

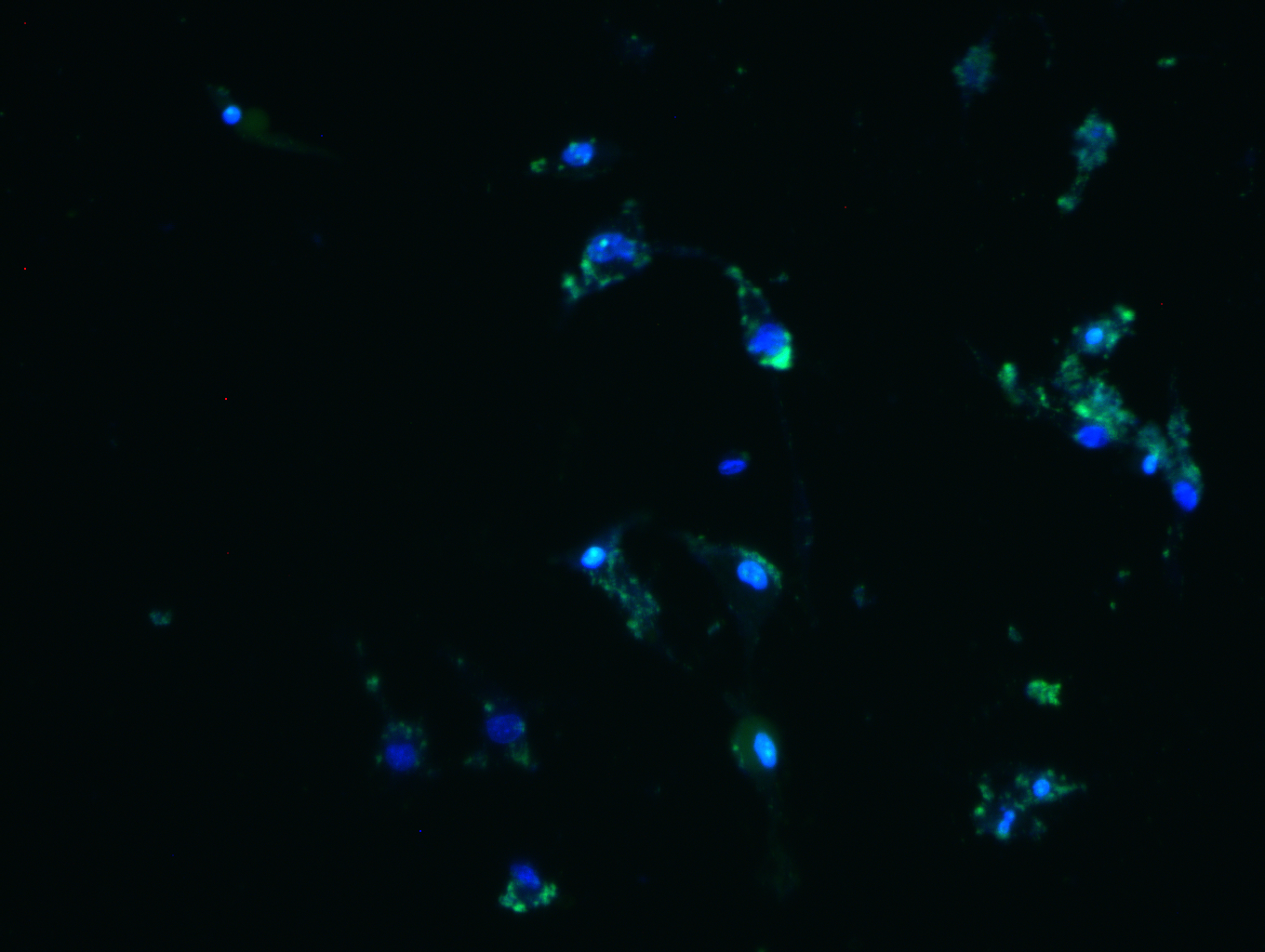

Supplement: S3 File — (ZIP) [file pone.0335890.s003.zip › Supporting Information3/Fig3/Fig3d/exo-bsa/1OK.png]

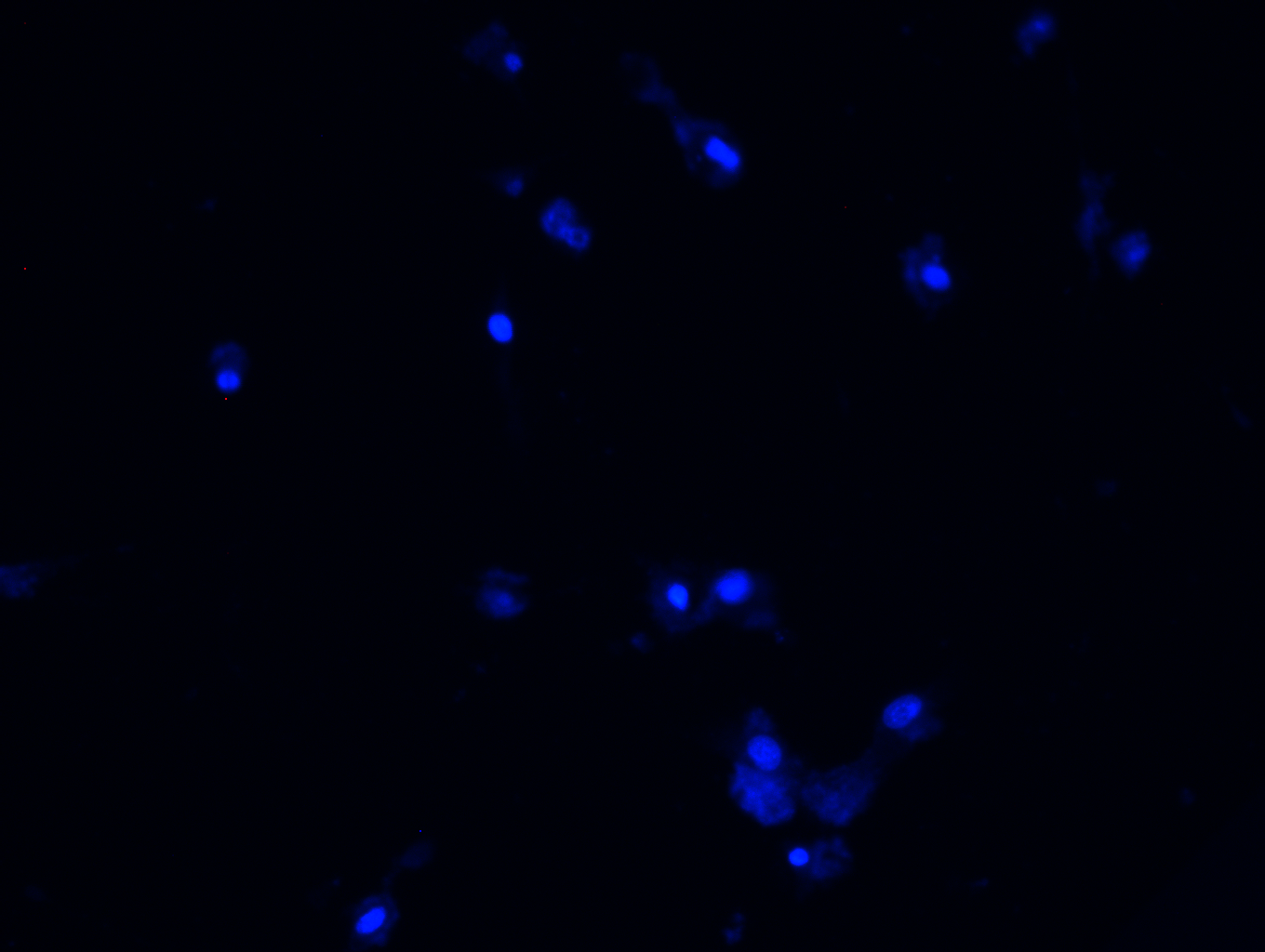

Supplement: S3 File — (ZIP) [file pone.0335890.s003.zip › Supporting Information3/Fig3/Fig3d/exo-bsa/2..png]

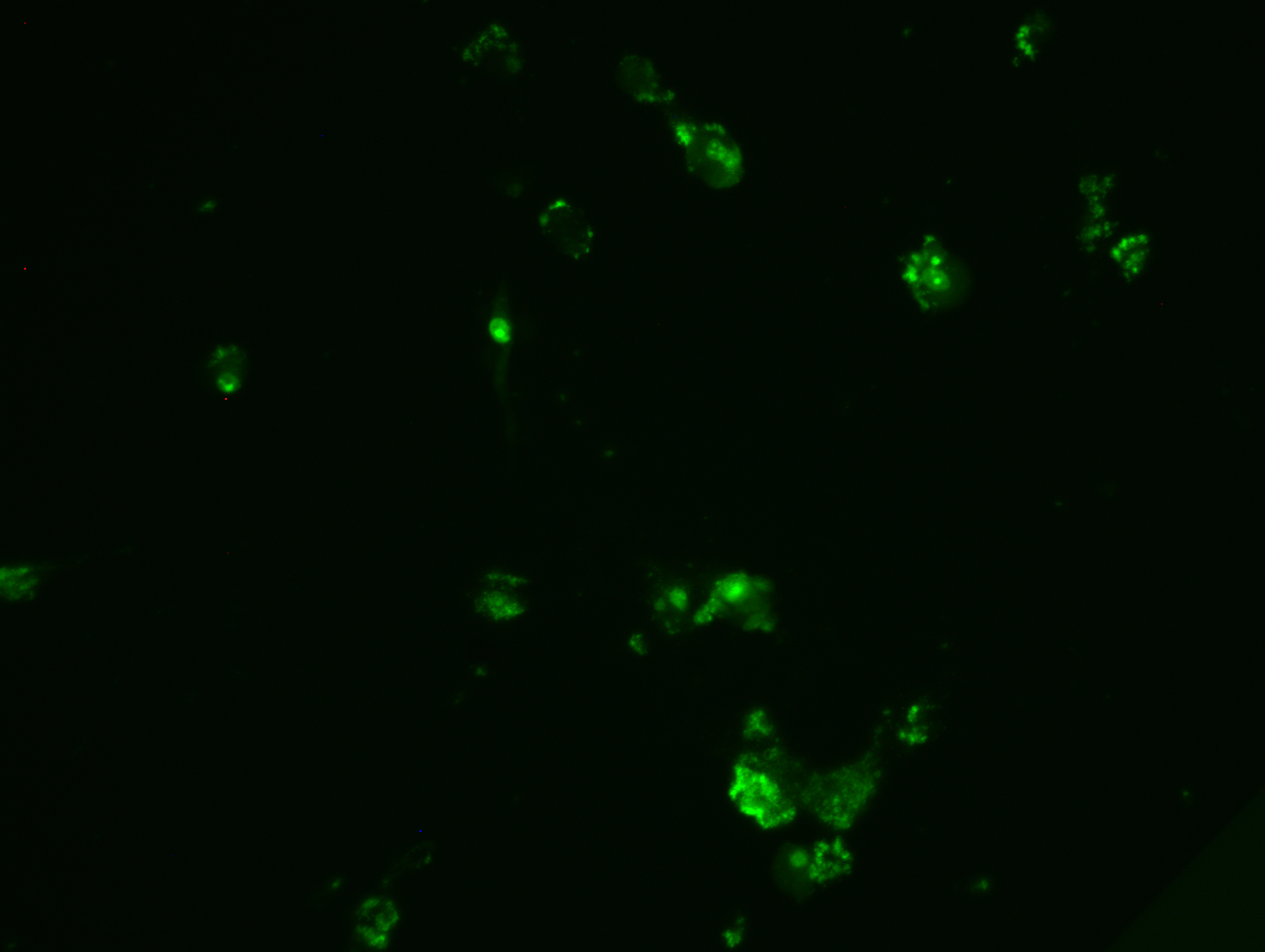

Supplement: S3 File — (ZIP) [file pone.0335890.s003.zip › Supporting Information3/Fig3/Fig3d/exo-bsa/2.png]

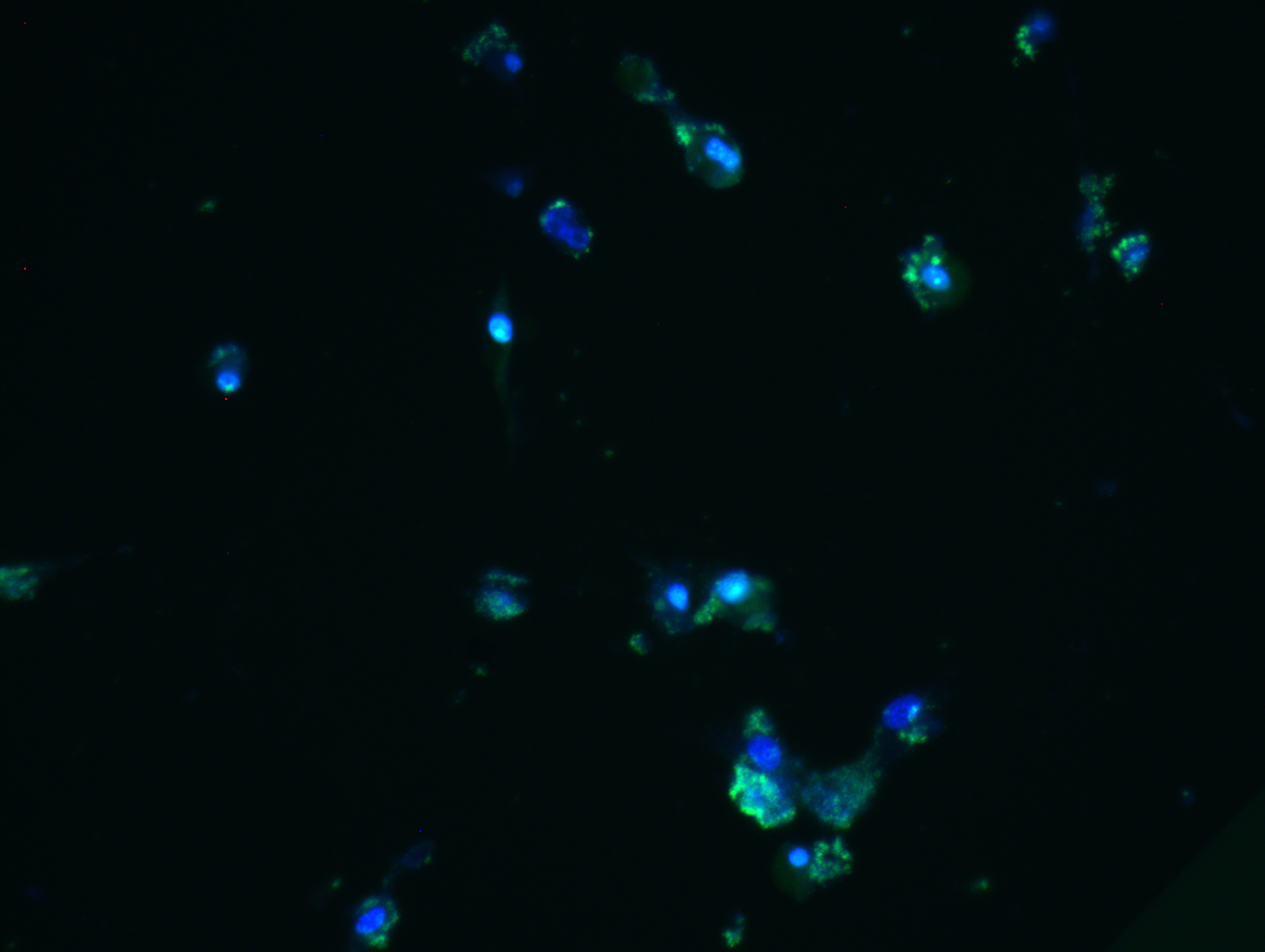

Supplement: S3 File — (ZIP) [file pone.0335890.s003.zip › Supporting Information3/Fig3/Fig3d/exo-bsa/2OK.png]

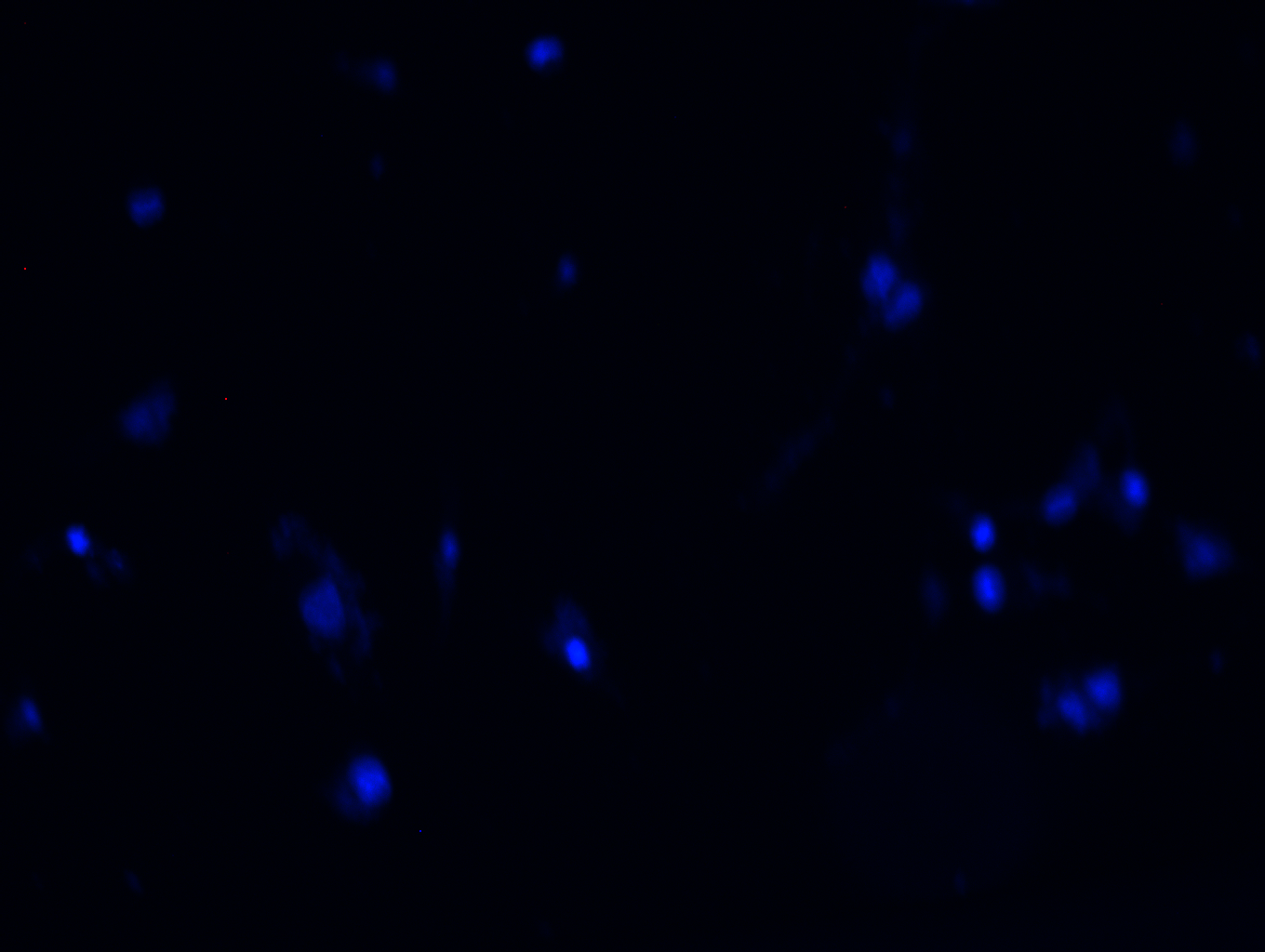

Supplement: S3 File — (ZIP) [file pone.0335890.s003.zip › Supporting Information3/Fig3/Fig3d/exo-bsa/3..png]

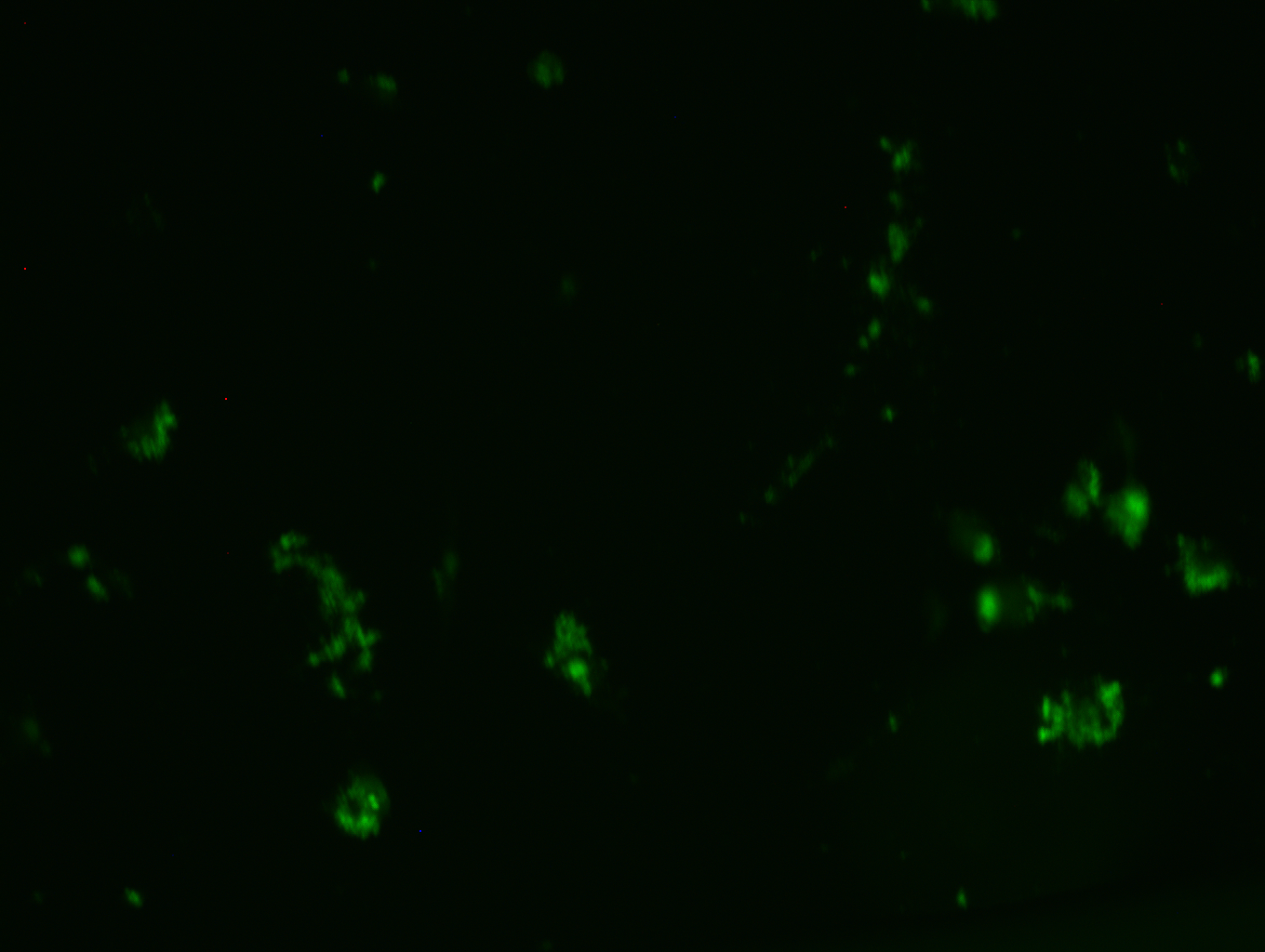

Supplement: S3 File — (ZIP) [file pone.0335890.s003.zip › Supporting Information3/Fig3/Fig3d/exo-bsa/3.png]

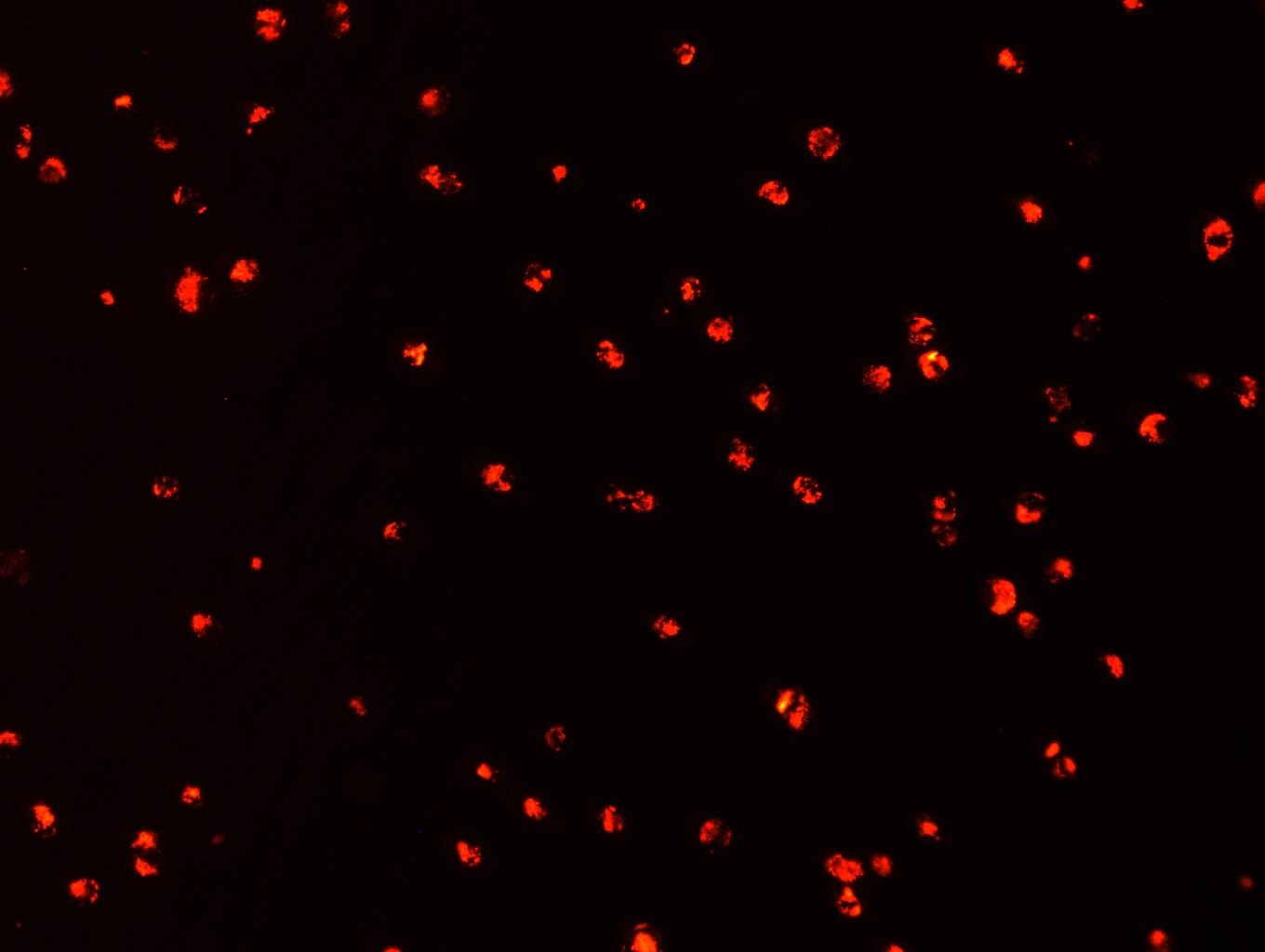

Supplement: S3 File — (ZIP) [file pone.0335890.s003.zip › Supporting Information3/Fig3/Fig3e/CTL/1..png]

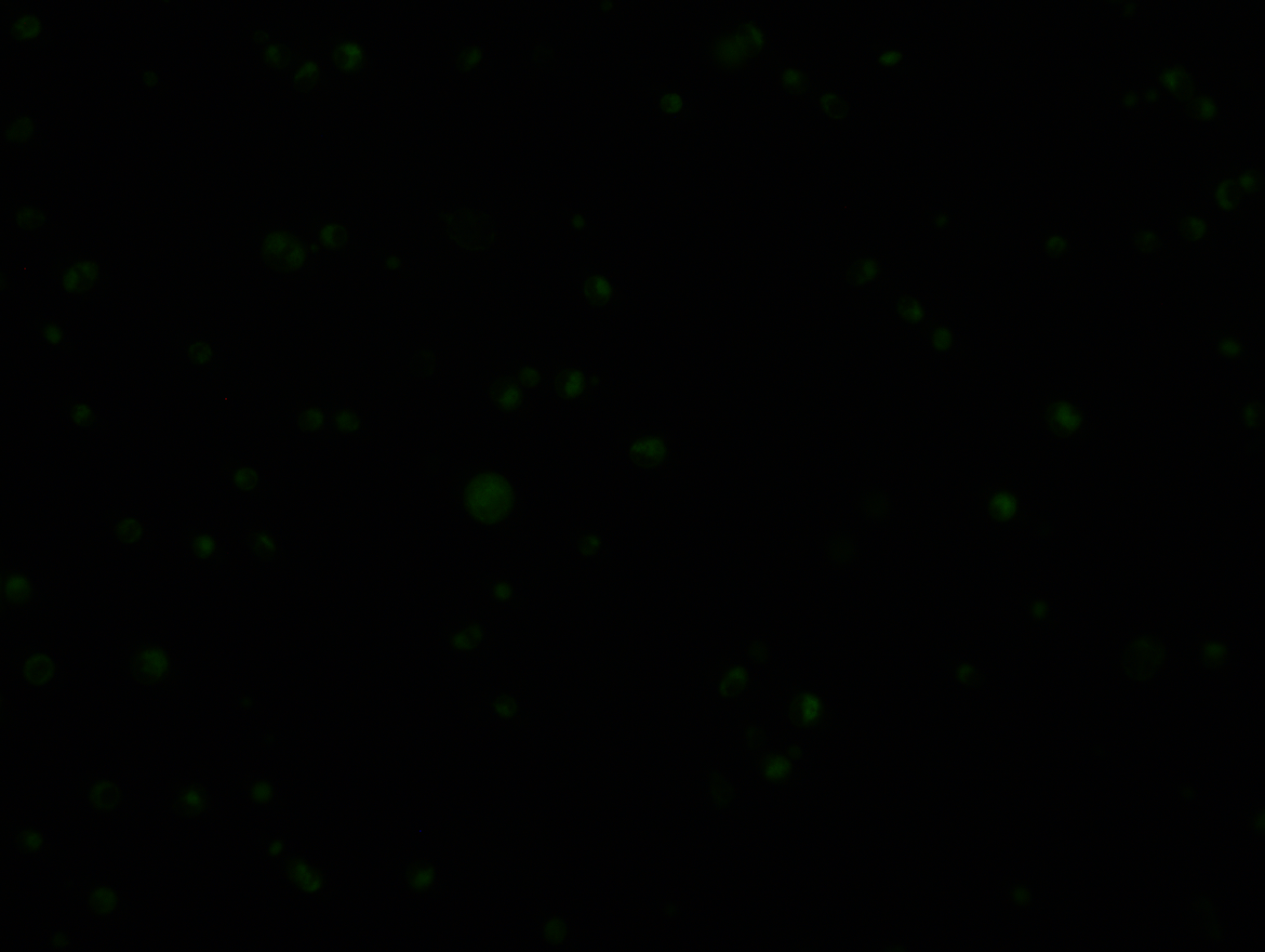

Supplement: S3 File — (ZIP) [file pone.0335890.s003.zip › Supporting Information3/Fig3/Fig3e/CTL/1.png]

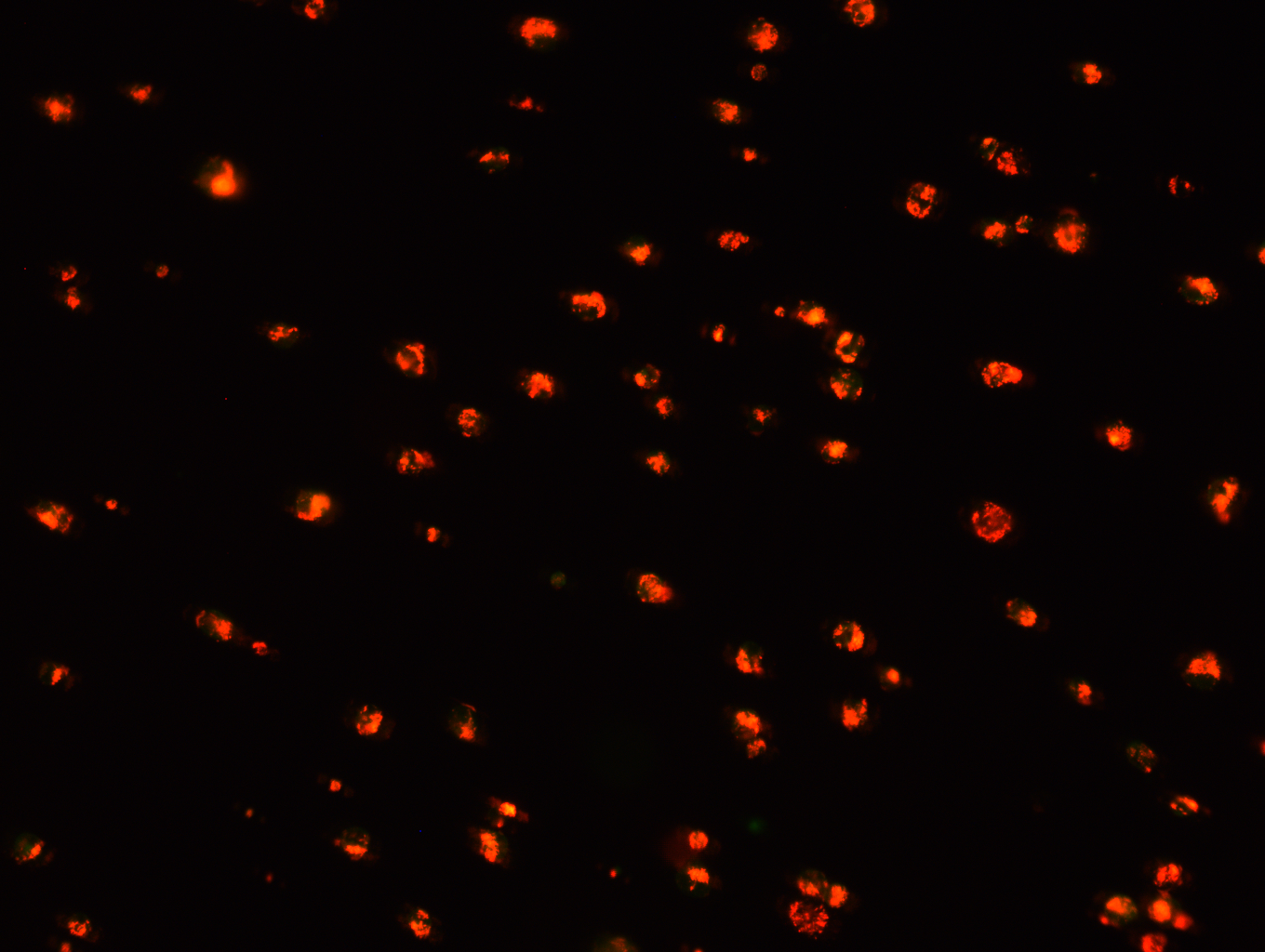

Supplement: S3 File — (ZIP) [file pone.0335890.s003.zip › Supporting Information3/Fig3/Fig3e/CTL/2..png]

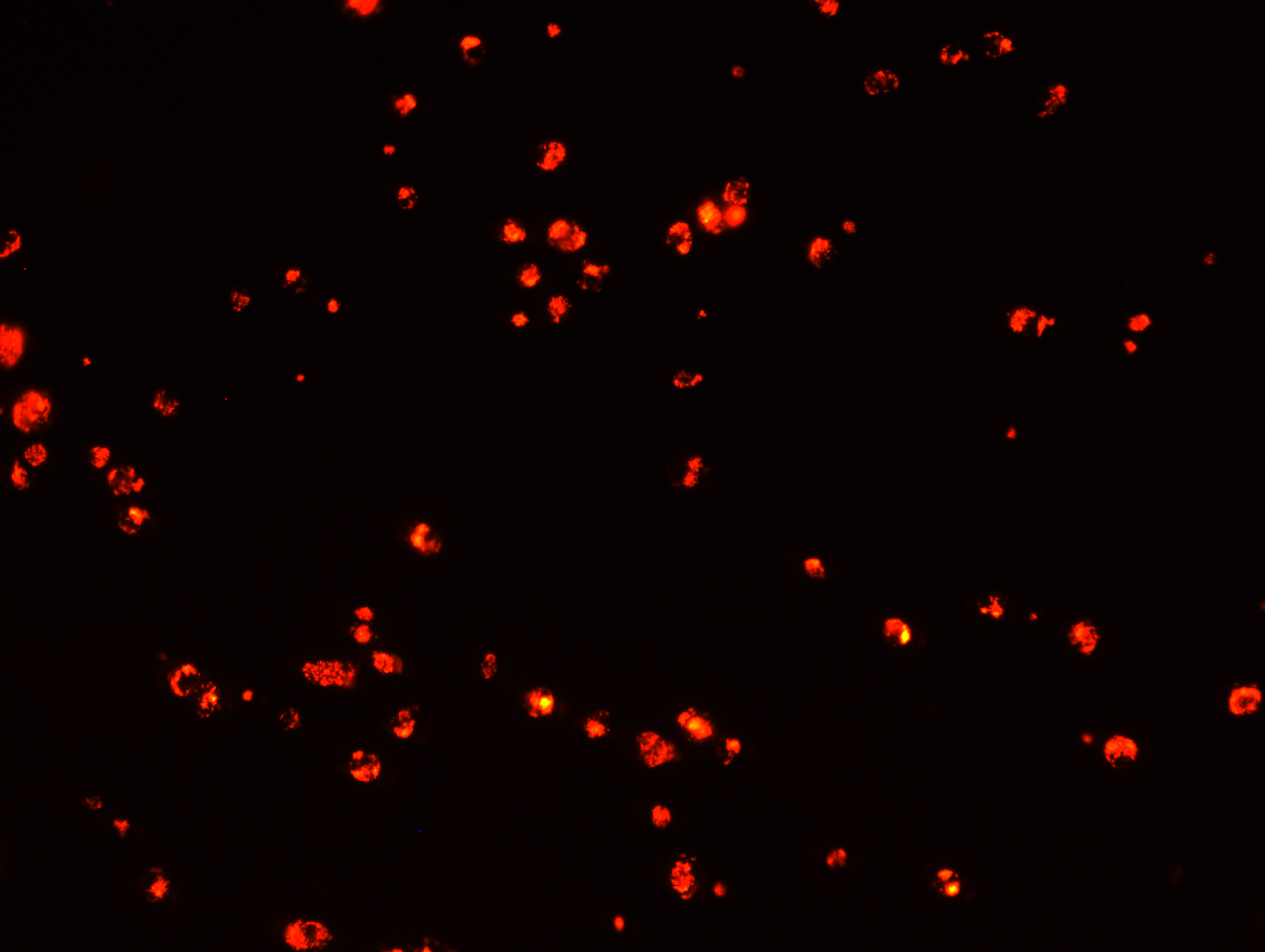

Supplement: S3 File — (ZIP) [file pone.0335890.s003.zip › Supporting Information3/Fig3/Fig3e/CTL/3..png]

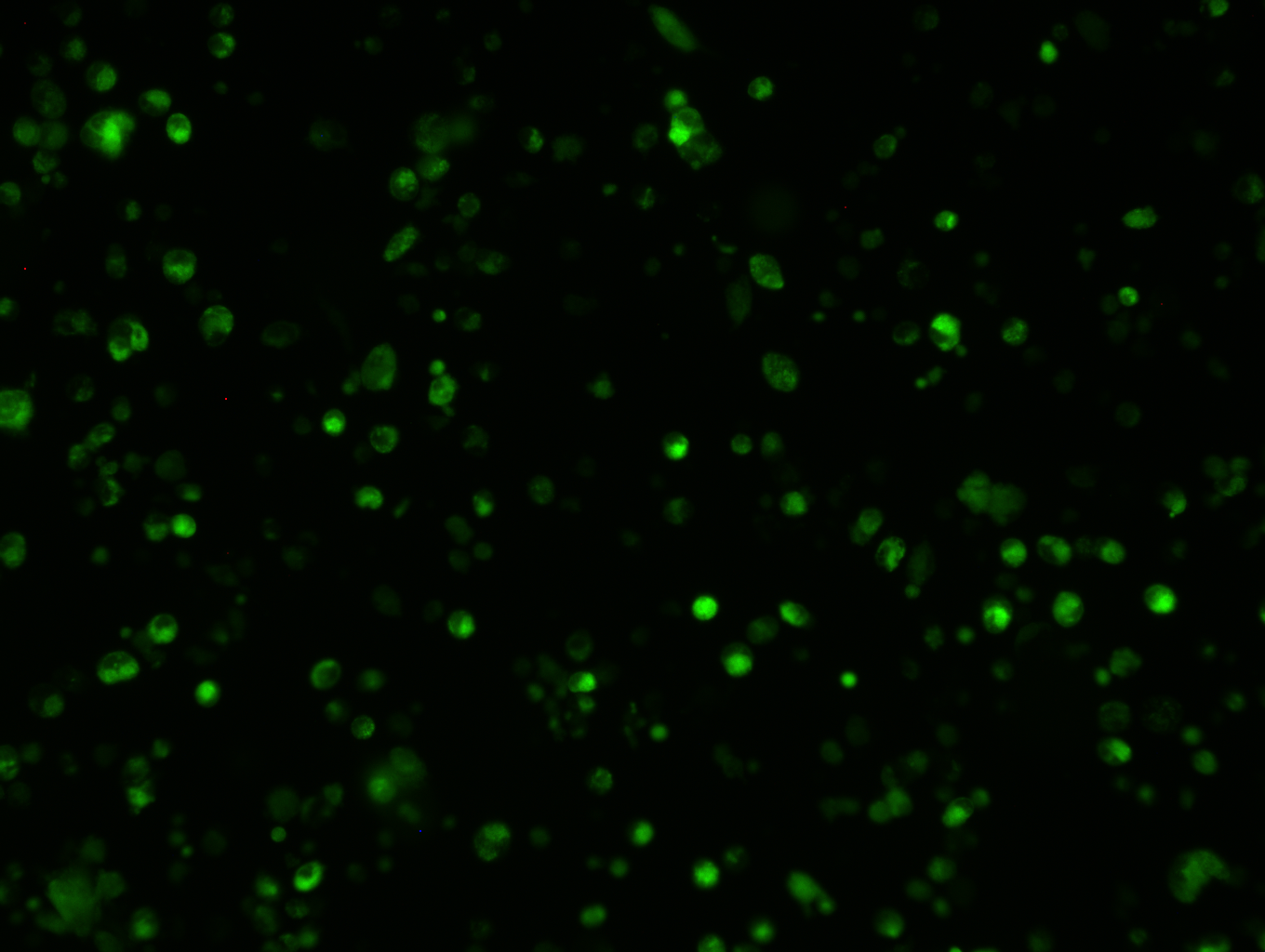

Supplement: S3 File — (ZIP) [file pone.0335890.s003.zip › Supporting Information3/Fig3/Fig3e/Exo-BSA@dbet6/1..png]

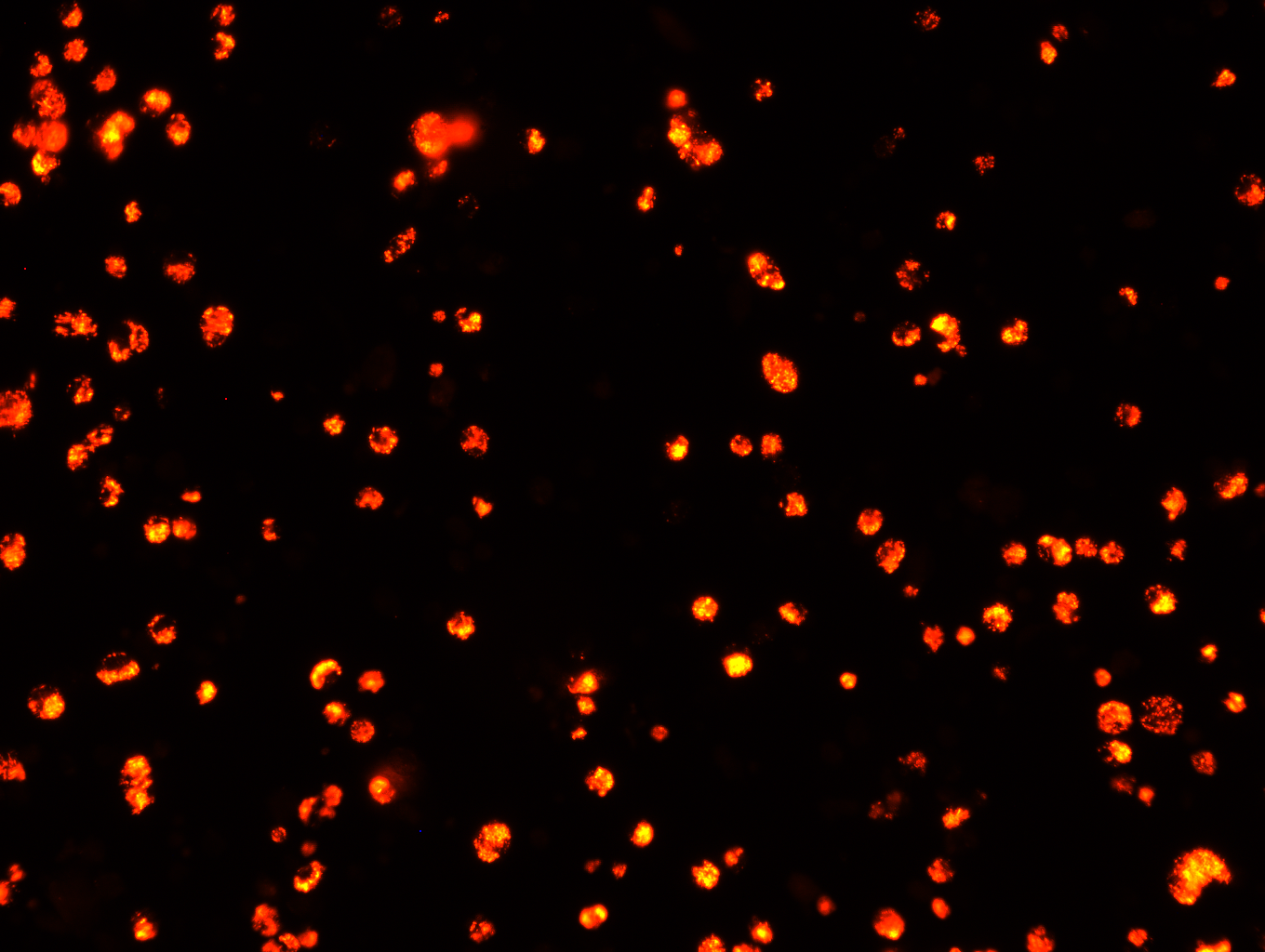

Supplement: S3 File — (ZIP) [file pone.0335890.s003.zip › Supporting Information3/Fig3/Fig3e/Exo-BSA@dbet6/1.png]

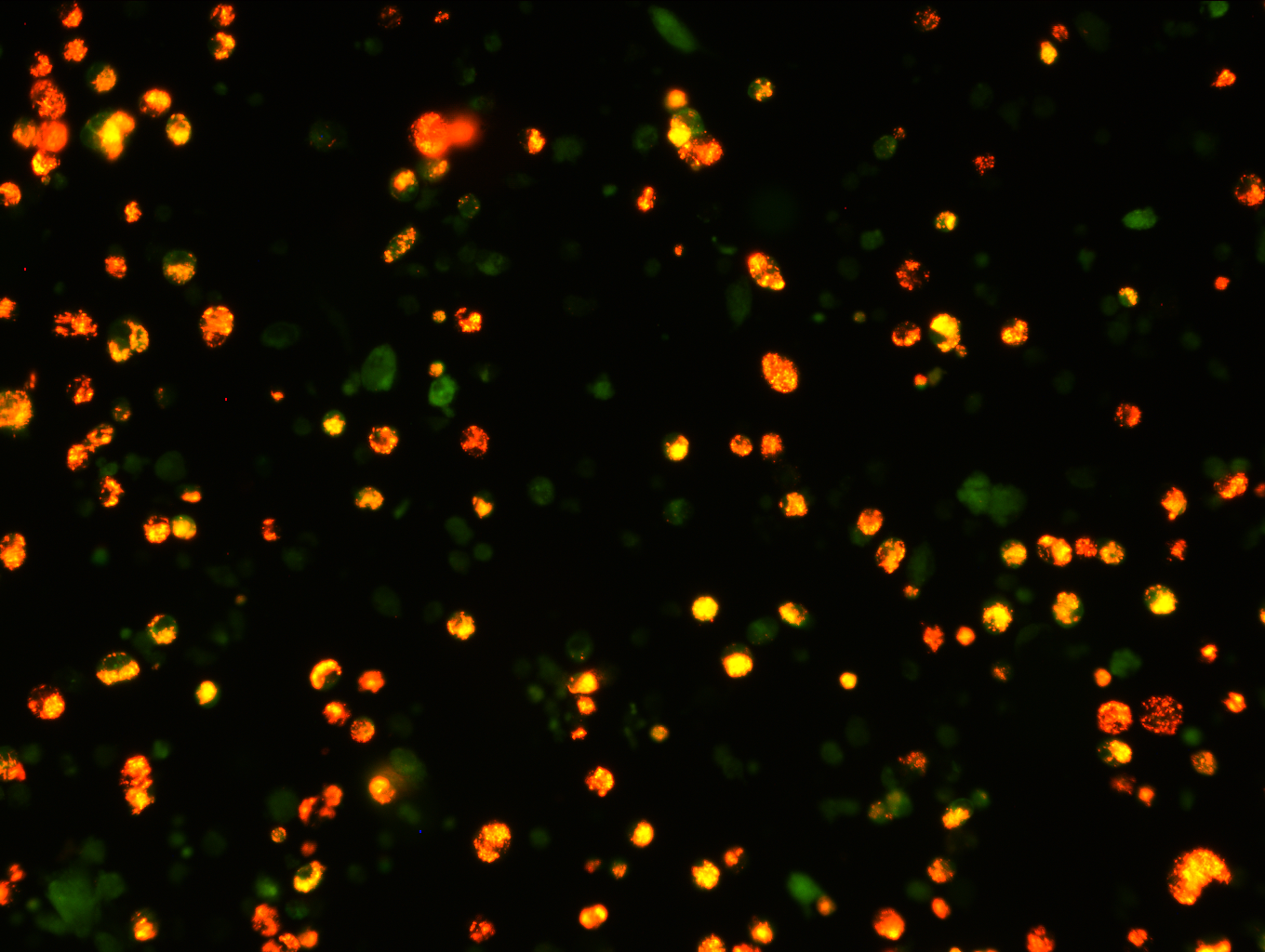

Supplement: S3 File — (ZIP) [file pone.0335890.s003.zip › Supporting Information3/Fig3/Fig3e/Exo-BSA@dbet6/1ok.png]

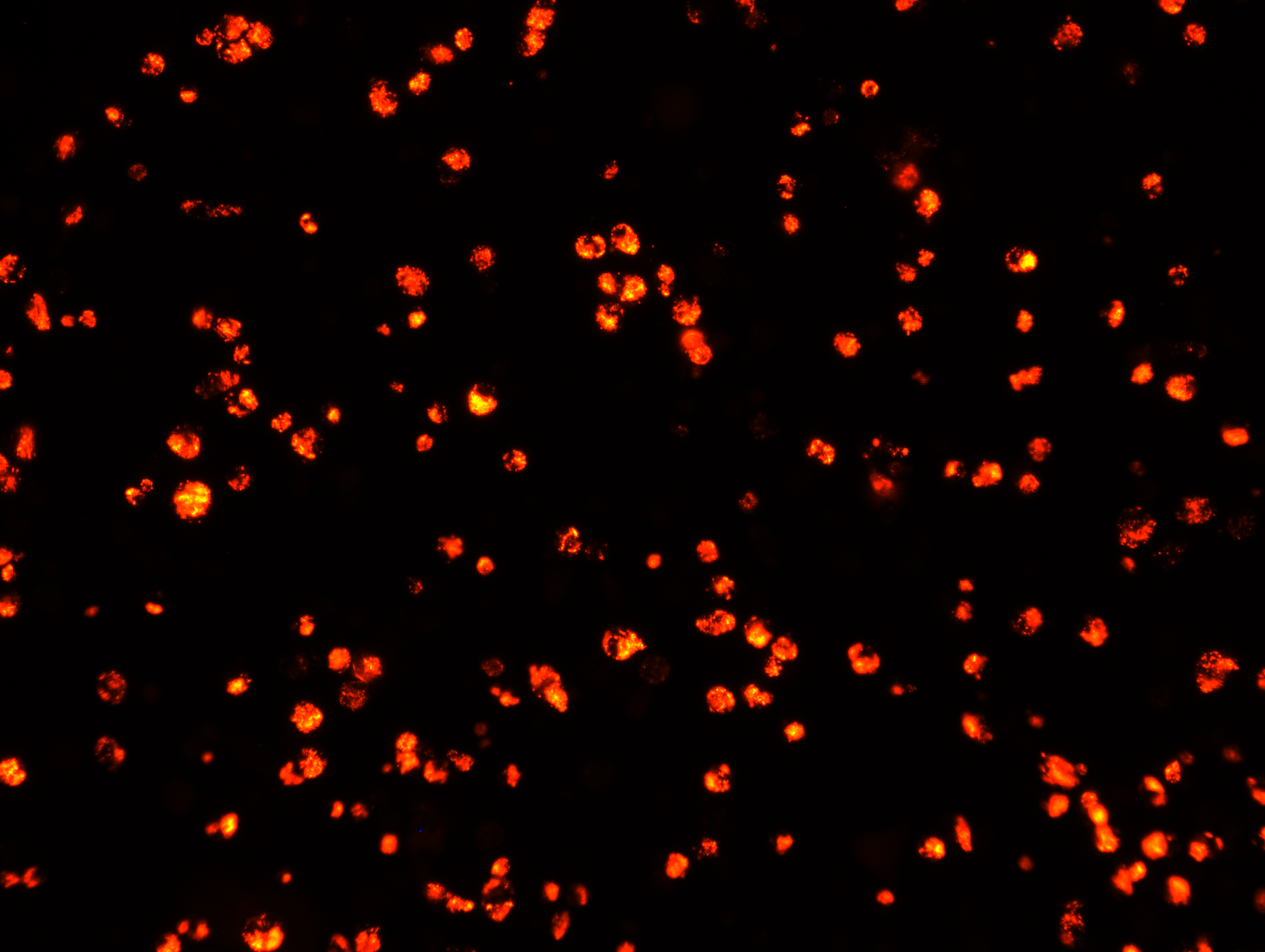

Supplement: S3 File — (ZIP) [file pone.0335890.s003.zip › Supporting Information3/Fig3/Fig3e/Exo-BSA@dbet6/2..png]

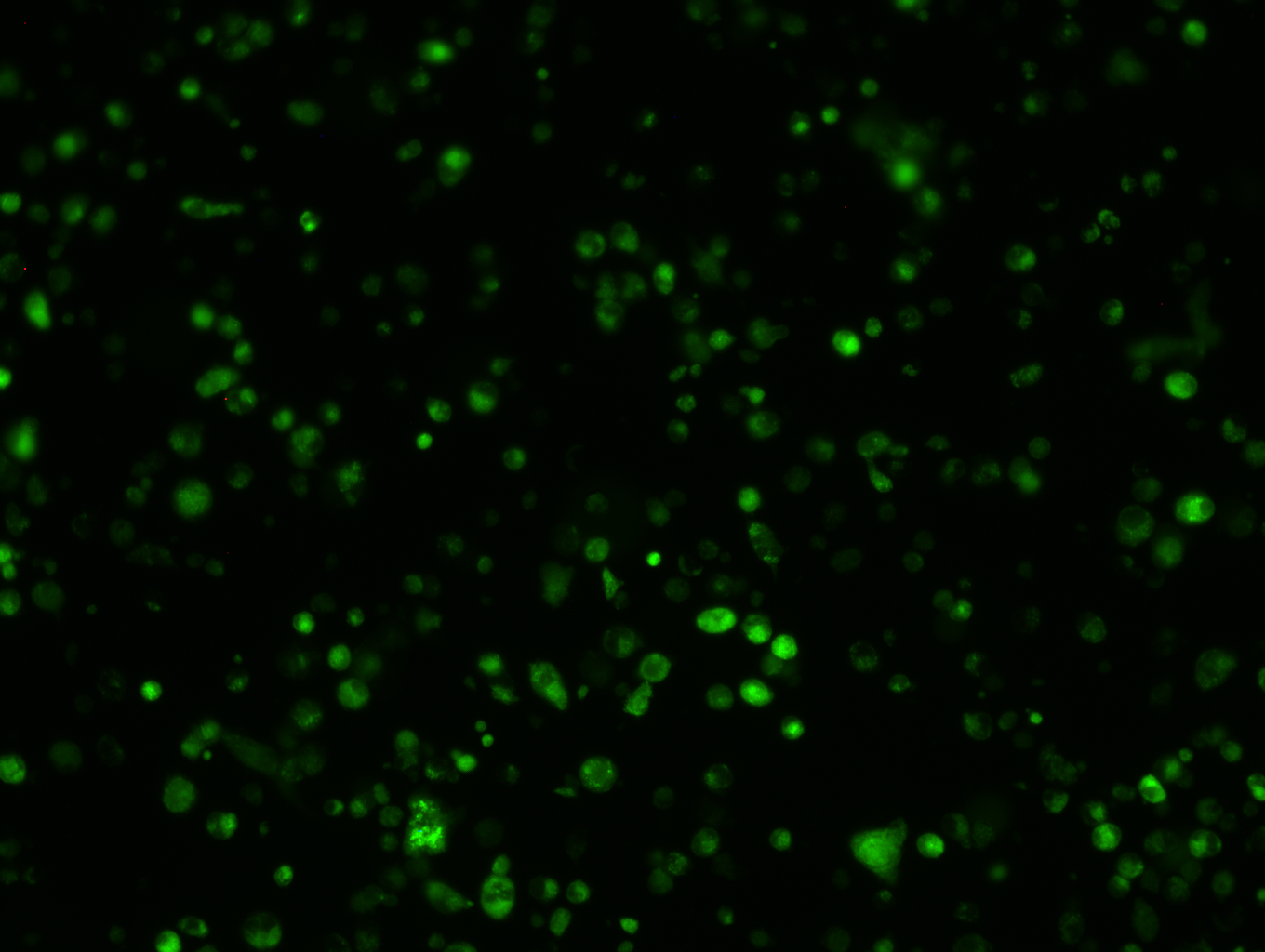

Supplement: S3 File — (ZIP) [file pone.0335890.s003.zip › Supporting Information3/Fig3/Fig3e/Exo-BSA@dbet6/2.png]

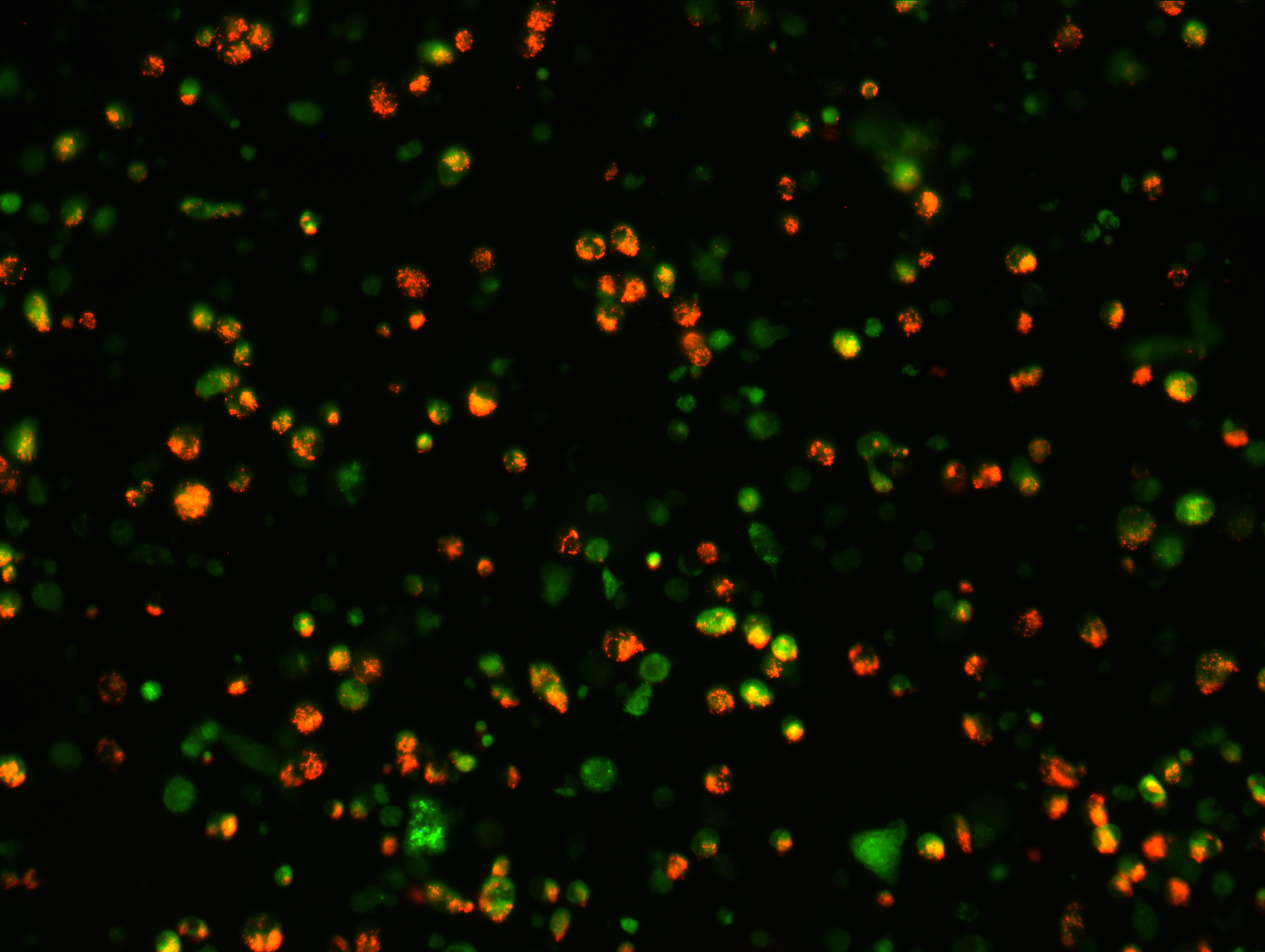

Supplement: S3 File — (ZIP) [file pone.0335890.s003.zip › Supporting Information3/Fig3/Fig3e/Exo-BSA@dbet6/2ok.png]

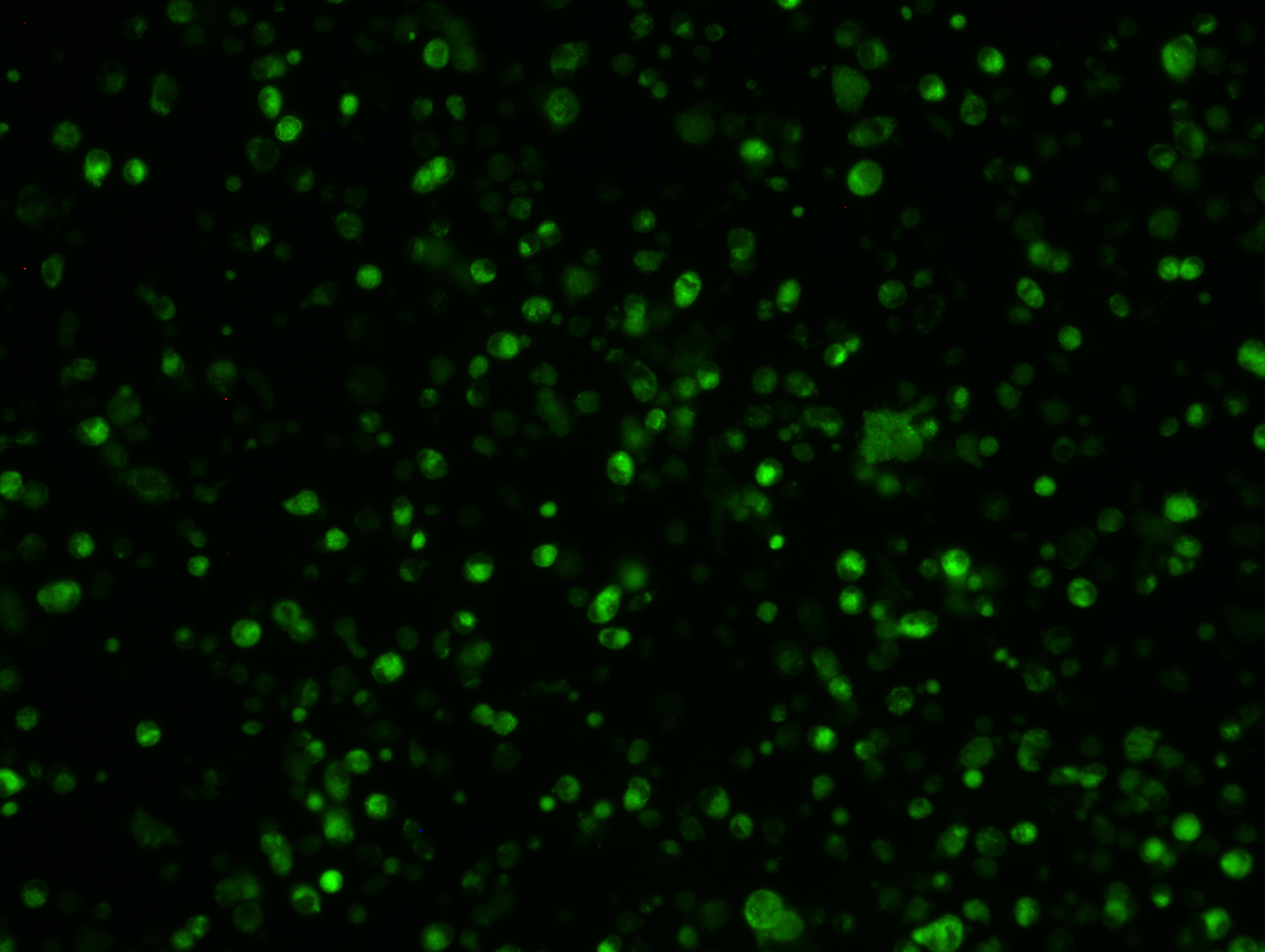

Supplement: S3 File — (ZIP) [file pone.0335890.s003.zip › Supporting Information3/Fig3/Fig3e/Exo-BSA@dbet6/3..png]

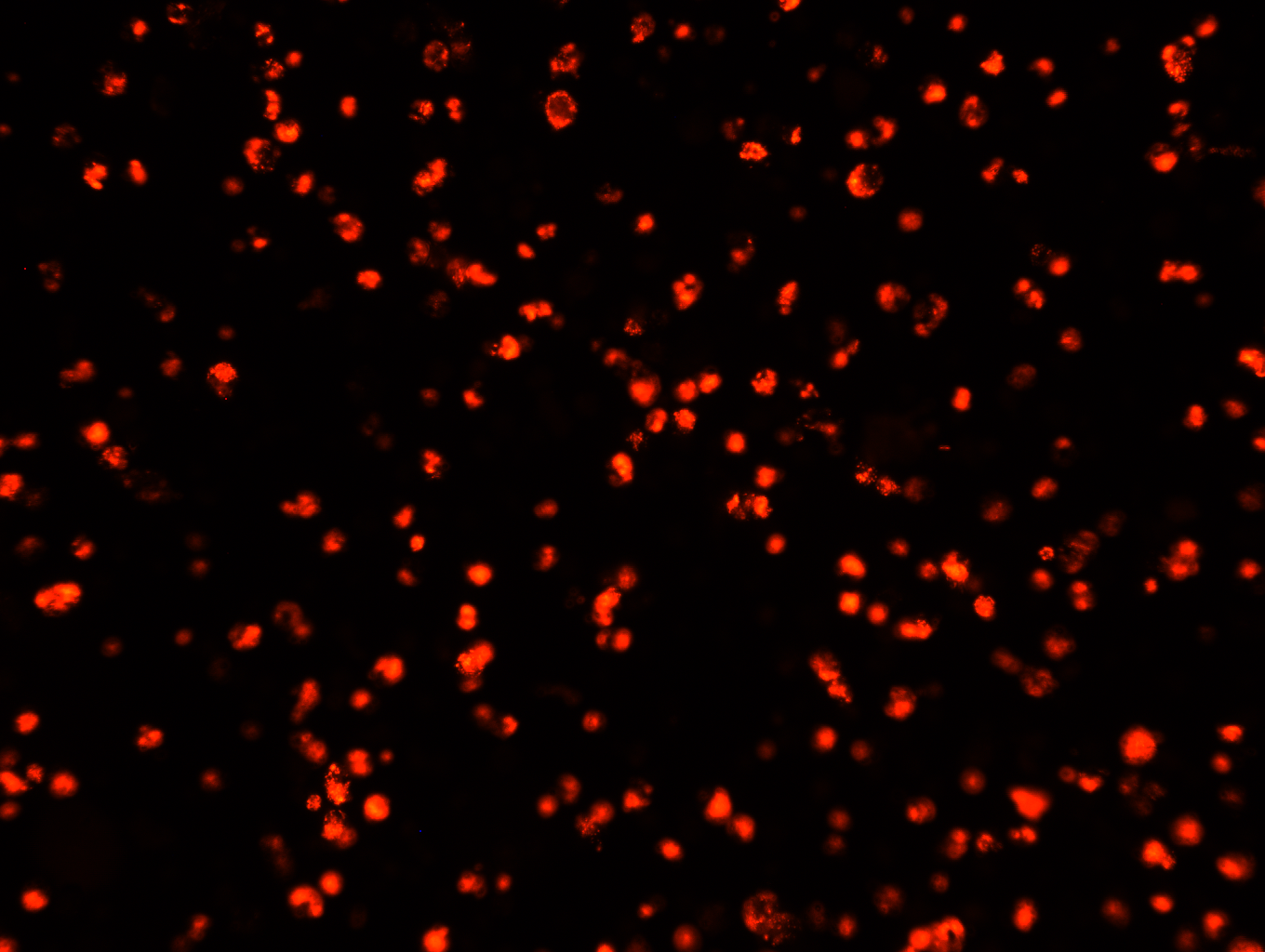

Supplement: S3 File — (ZIP) [file pone.0335890.s003.zip › Supporting Information3/Fig3/Fig3e/Exo-BSA@dbet6/3.png]

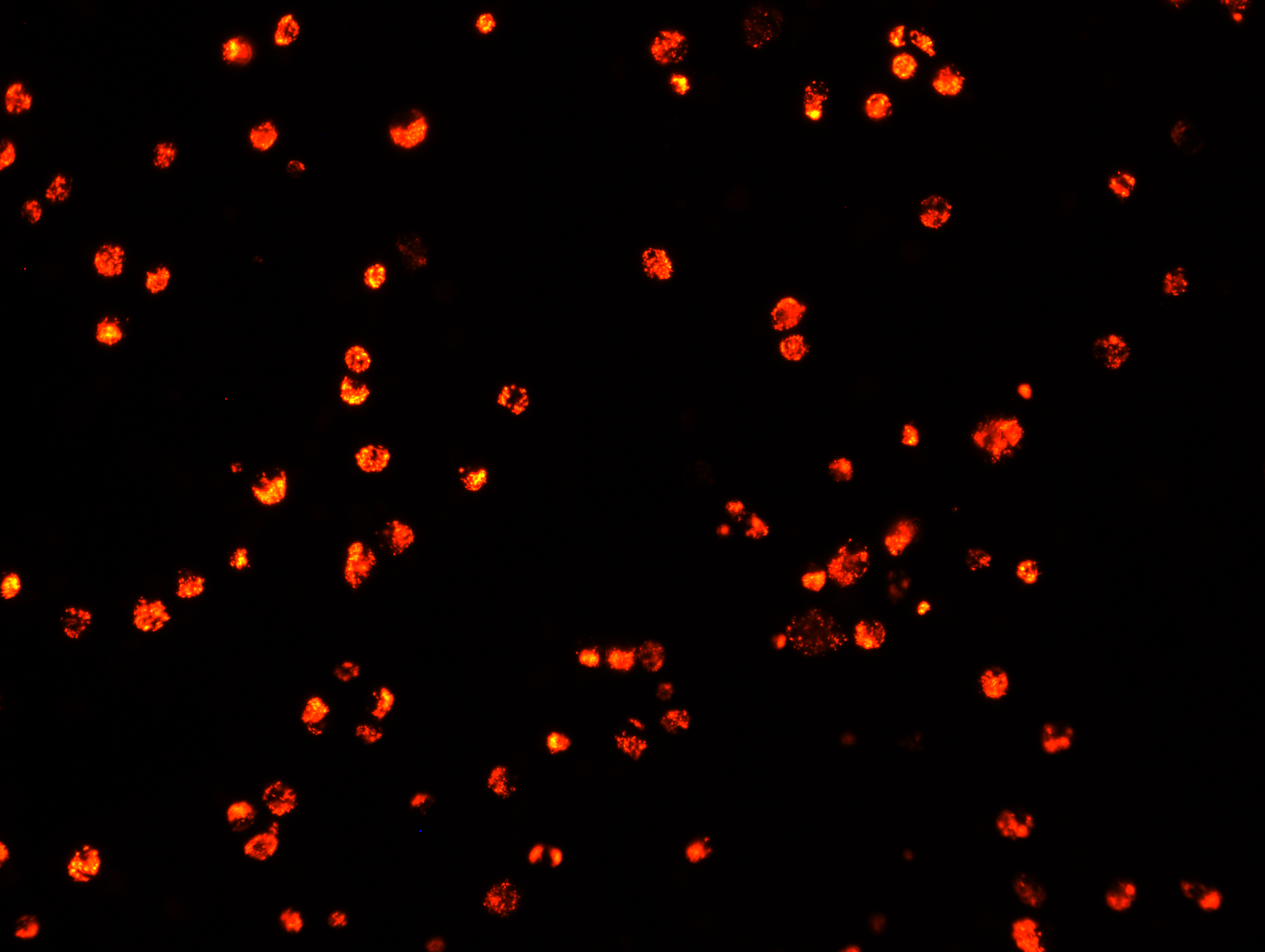

Supplement: S3 File — (ZIP) [file pone.0335890.s003.zip › Supporting Information3/Fig3/Fig3e/dbet6/1..png]

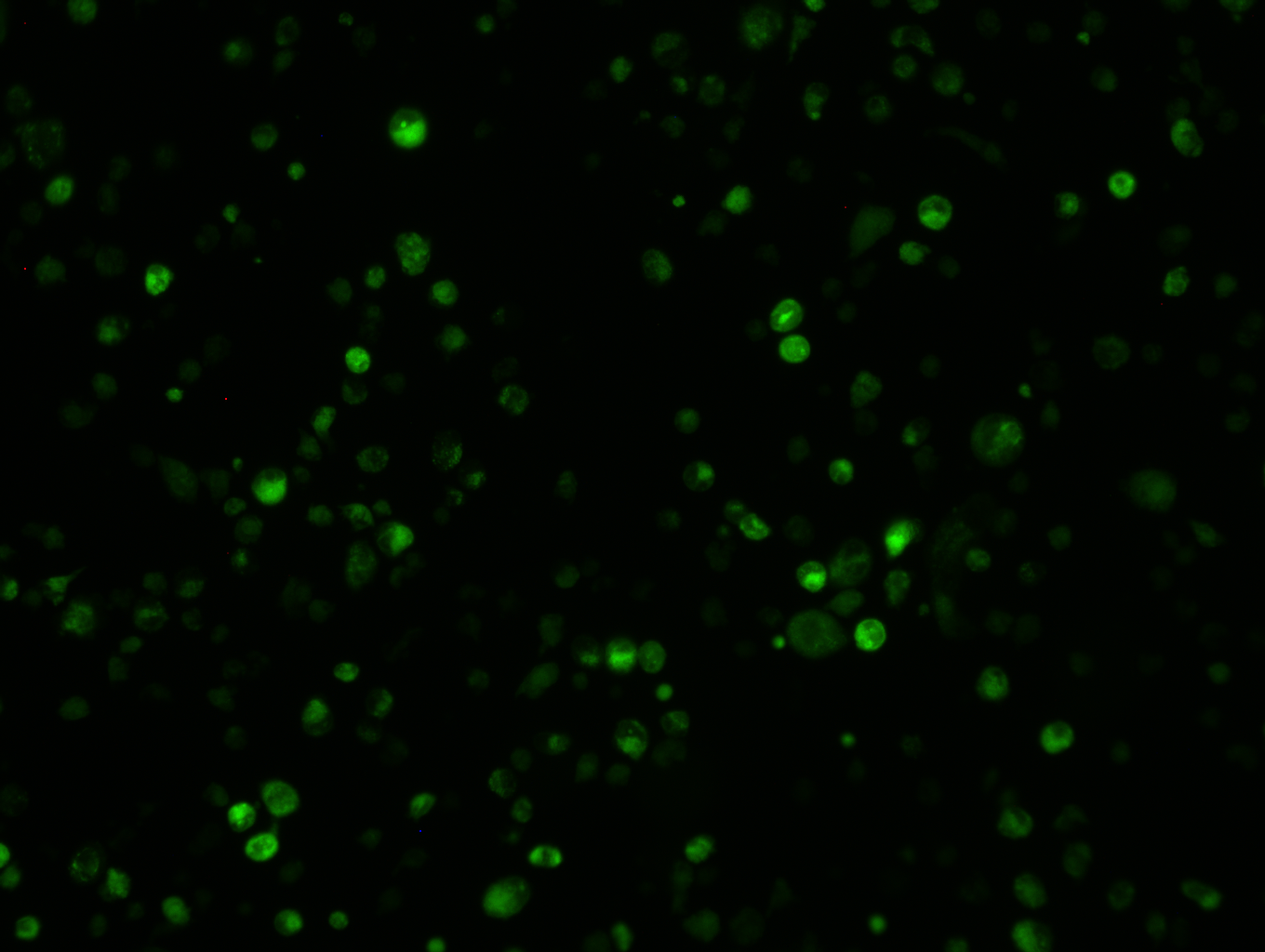

Supplement: S3 File — (ZIP) [file pone.0335890.s003.zip › Supporting Information3/Fig3/Fig3e/dbet6/1.png]

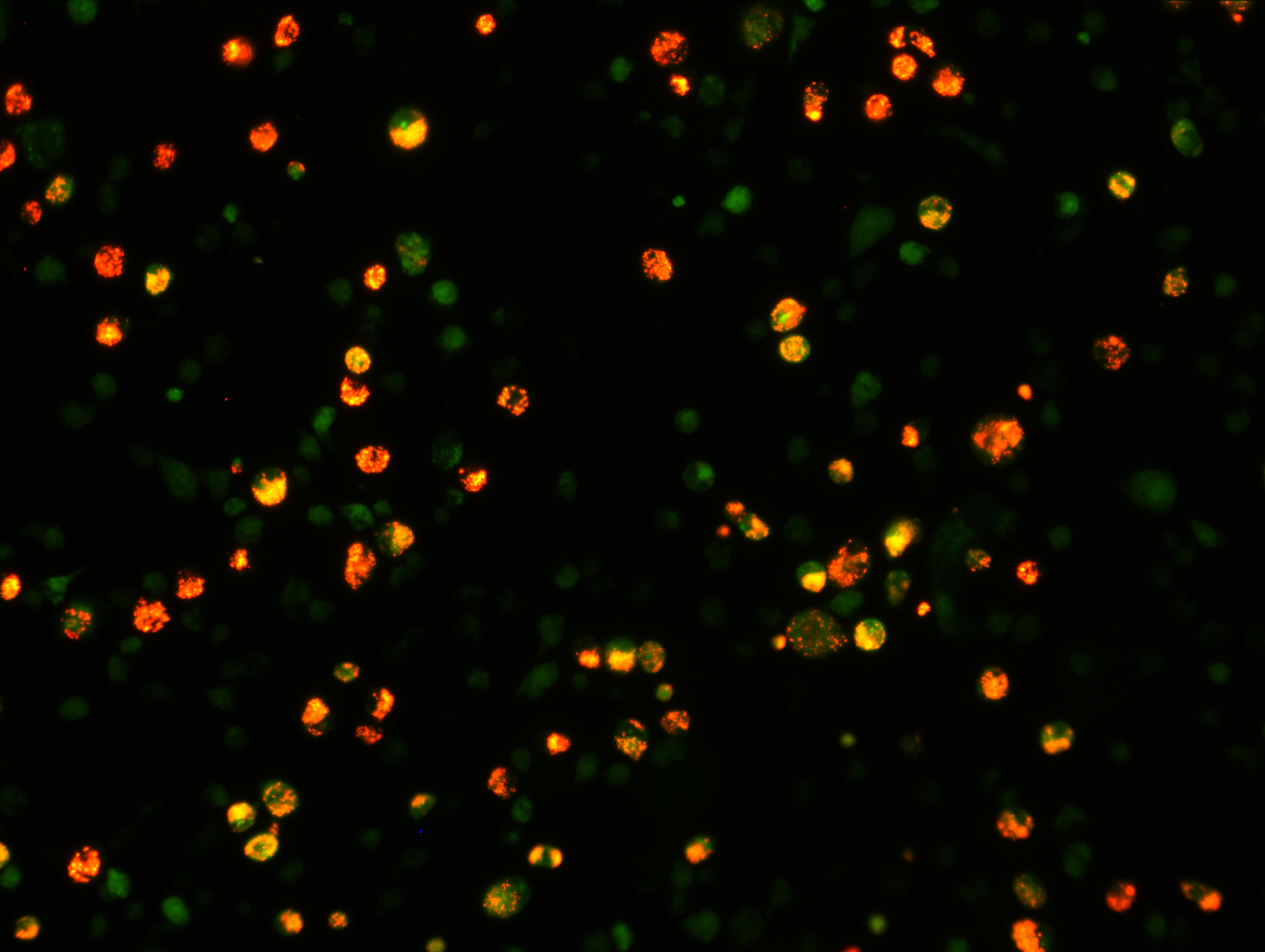

Supplement: S3 File — (ZIP) [file pone.0335890.s003.zip › Supporting Information3/Fig3/Fig3e/dbet6/1ok.png]

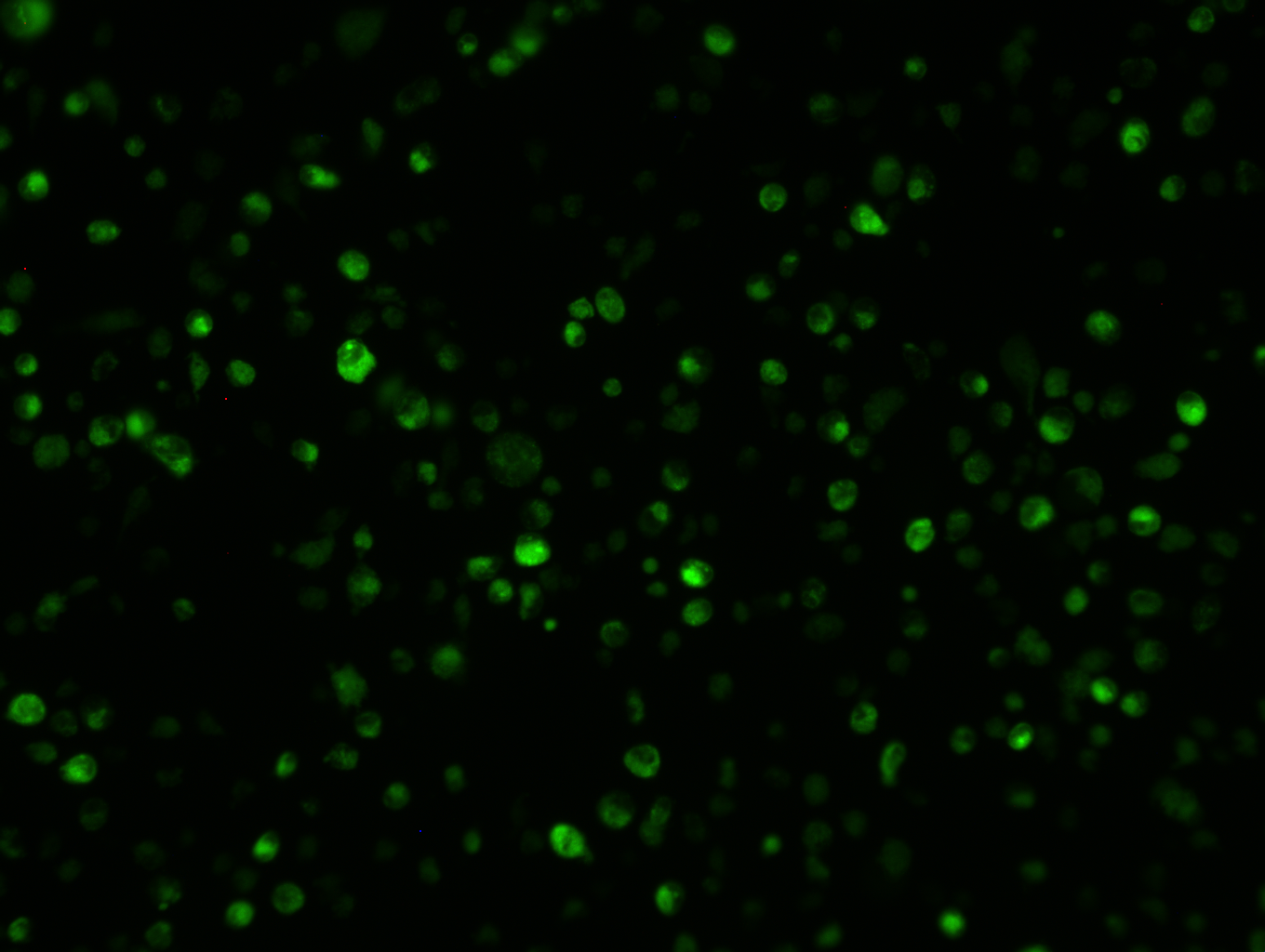

Supplement: S3 File — (ZIP) [file pone.0335890.s003.zip › Supporting Information3/Fig3/Fig3e/dbet6/2..png]

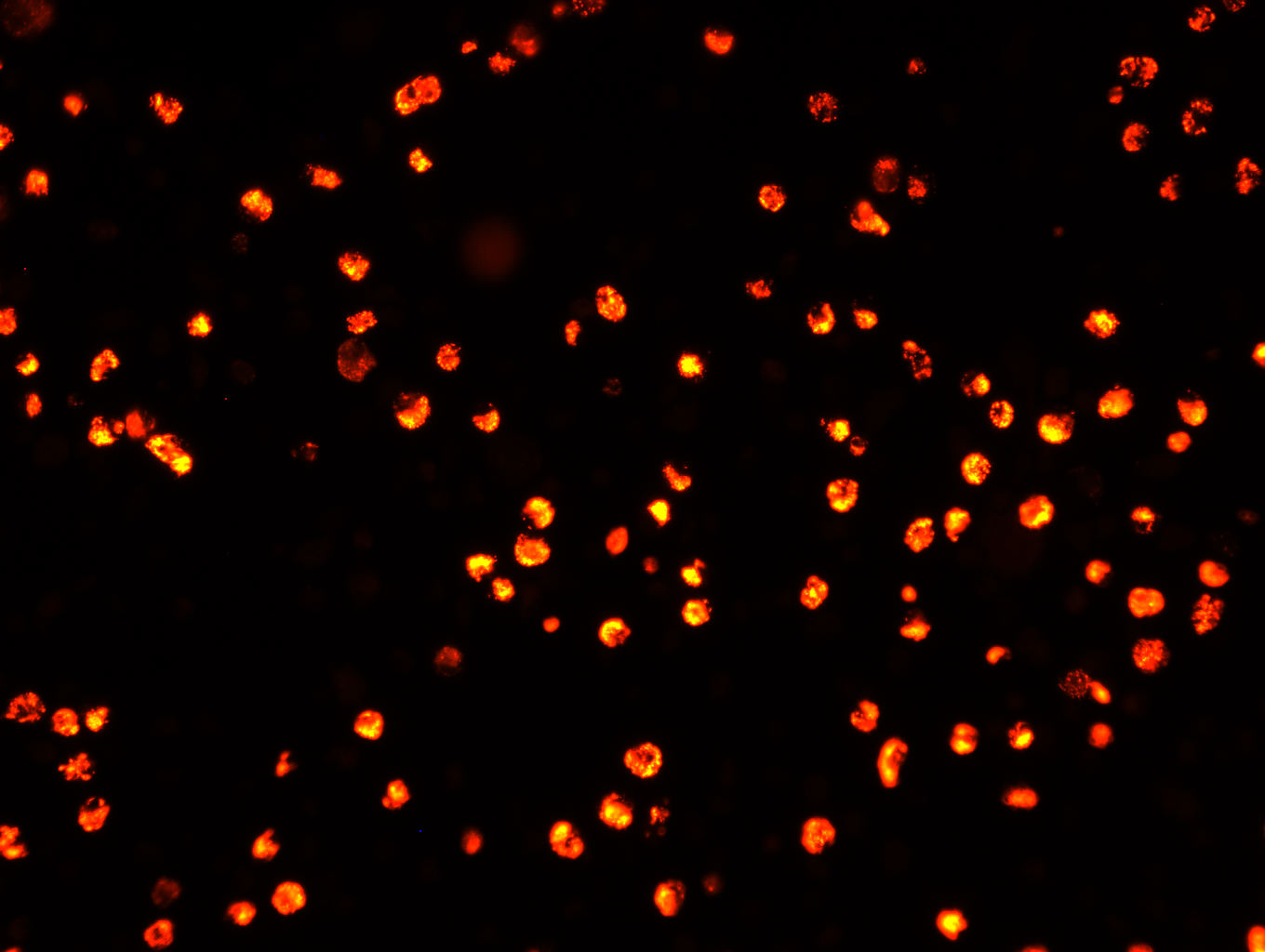

Supplement: S3 File — (ZIP) [file pone.0335890.s003.zip › Supporting Information3/Fig3/Fig3e/dbet6/2.png]

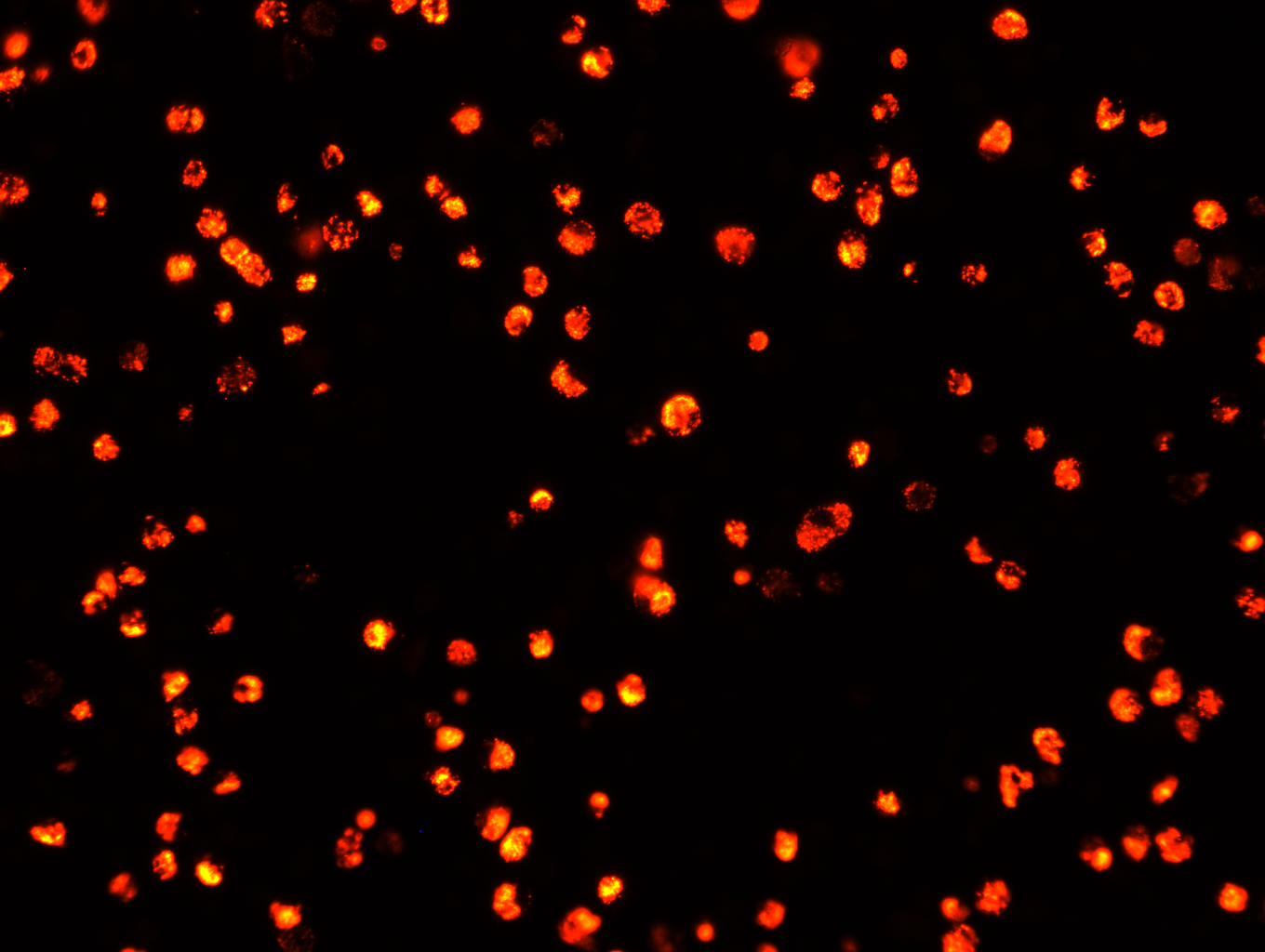

Supplement: S3 File — (ZIP) [file pone.0335890.s003.zip › Supporting Information3/Fig3/Fig3e/dbet6/3..png]

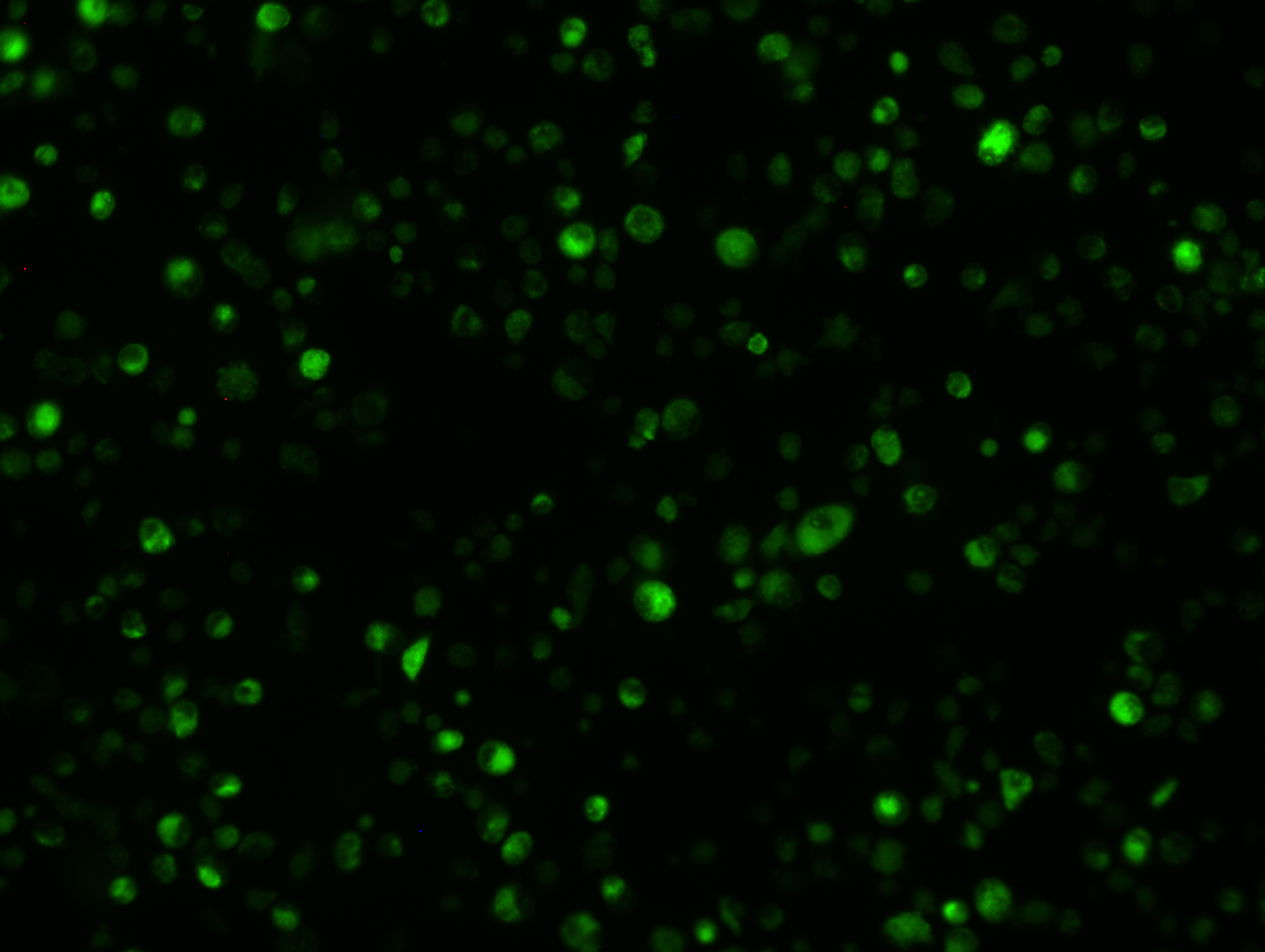

Supplement: S3 File — (ZIP) [file pone.0335890.s003.zip › Supporting Information3/Fig3/Fig3e/dbet6/3.png]

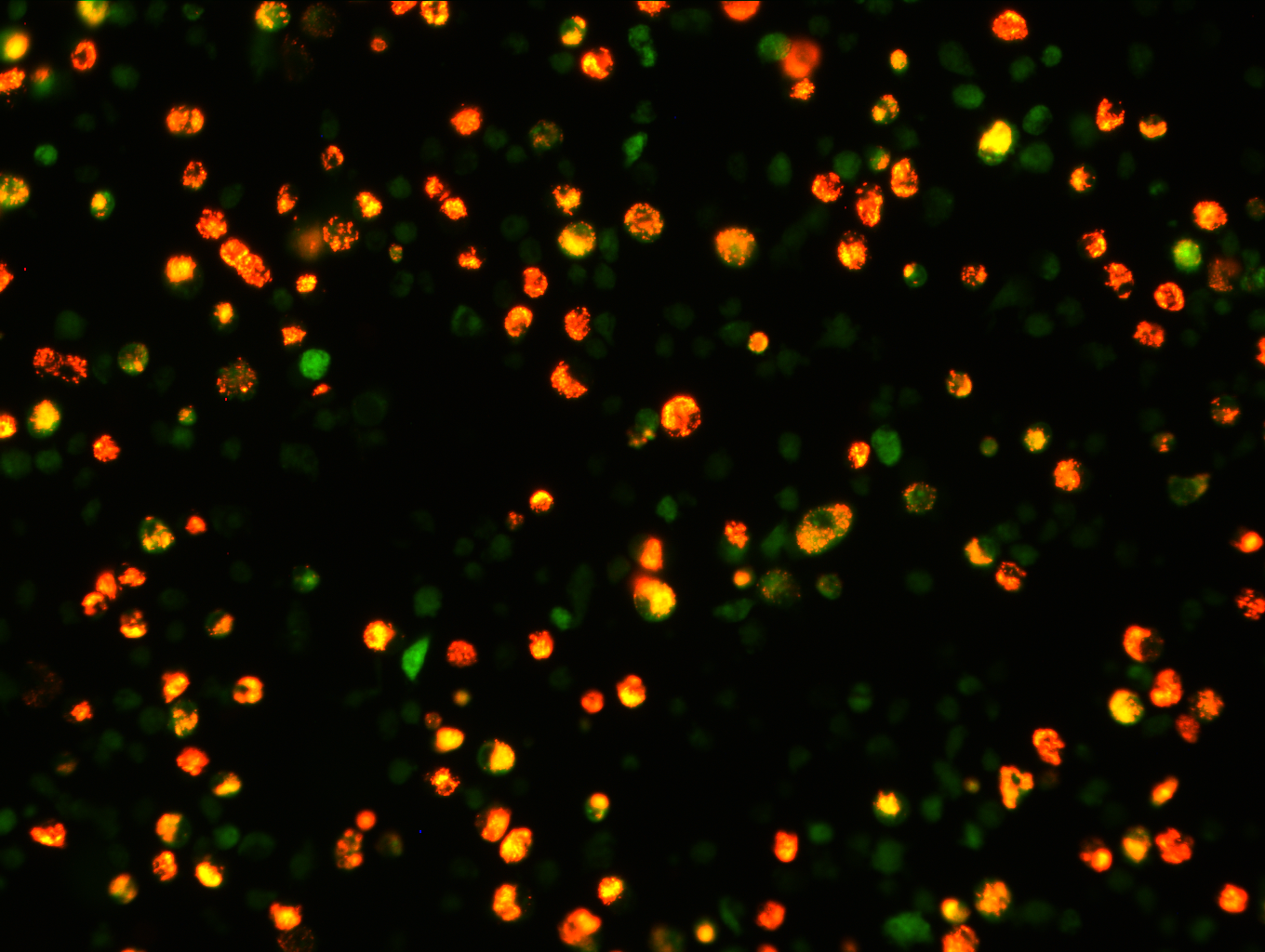

Supplement: S3 File — (ZIP) [file pone.0335890.s003.zip › Supporting Information3/Fig3/Fig3e/dbet6/3ok.png]

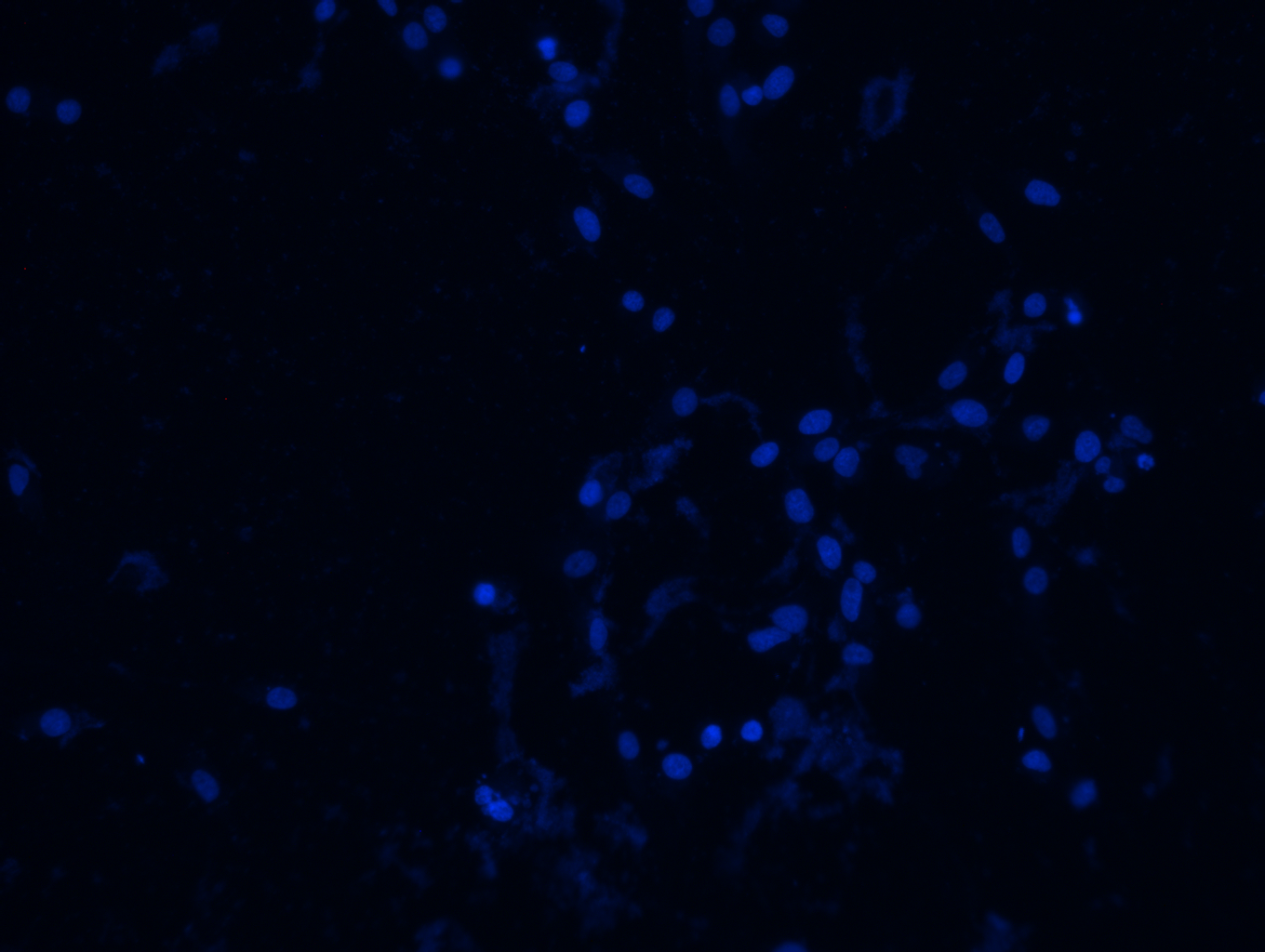

Supplement: S3 File — (ZIP) [file pone.0335890.s003.zip › Supporting Information3/Fig3/Fig3f/CTL/1..png]

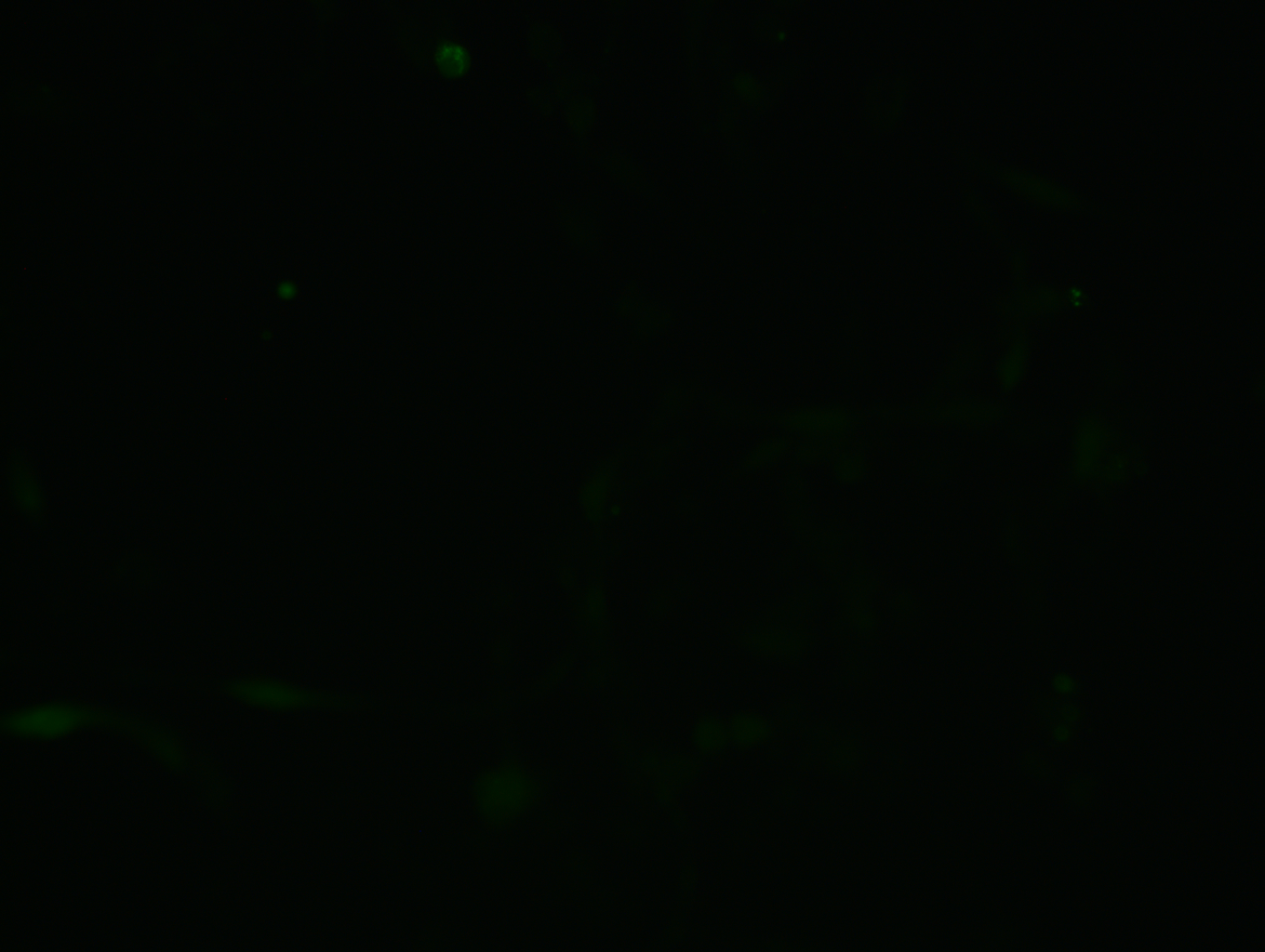

Supplement: S3 File — (ZIP) [file pone.0335890.s003.zip › Supporting Information3/Fig3/Fig3f/CTL/1.png]

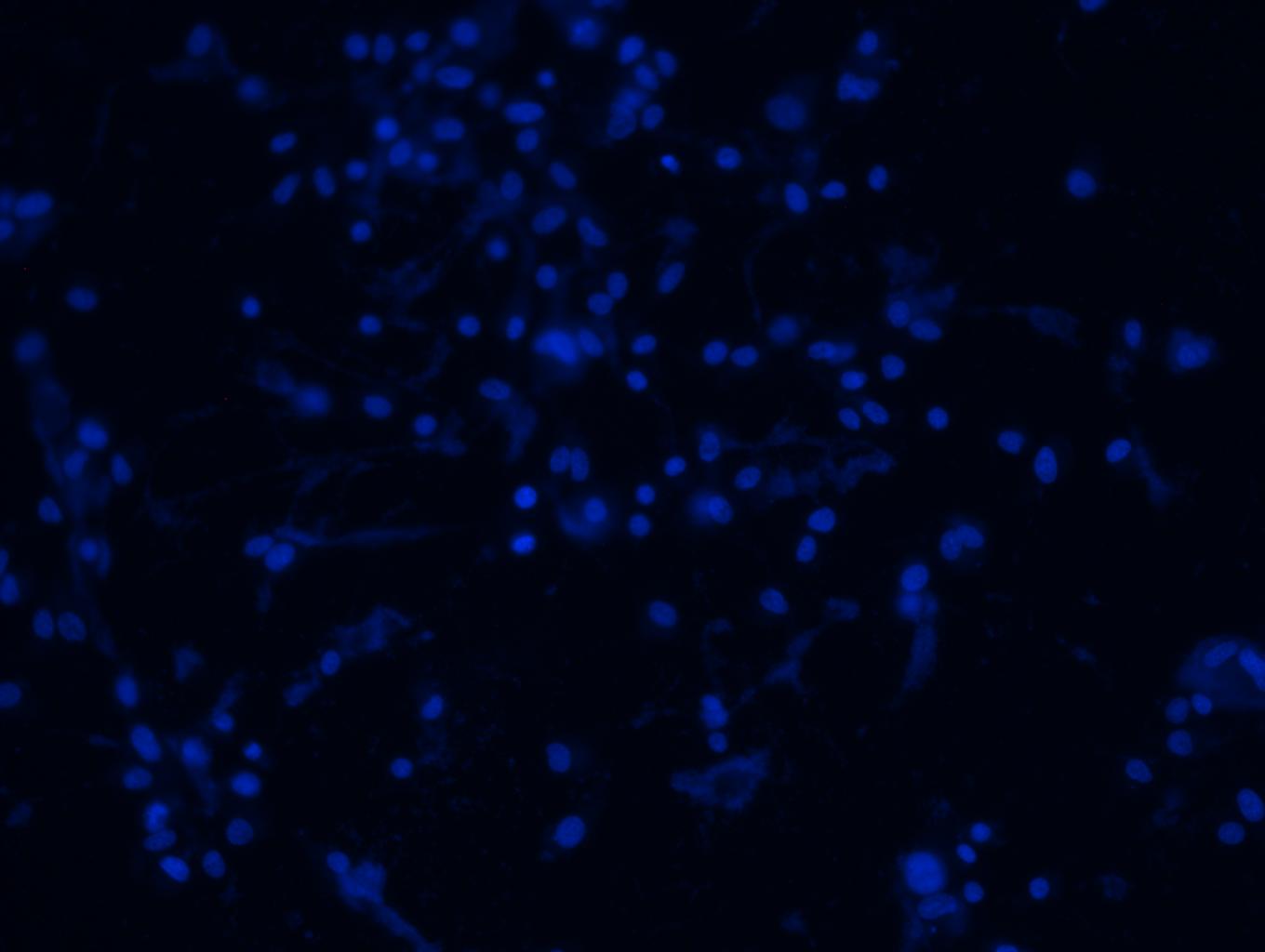

Supplement: S3 File — (ZIP) [file pone.0335890.s003.zip › Supporting Information3/Fig3/Fig3f/CTL/2..png]

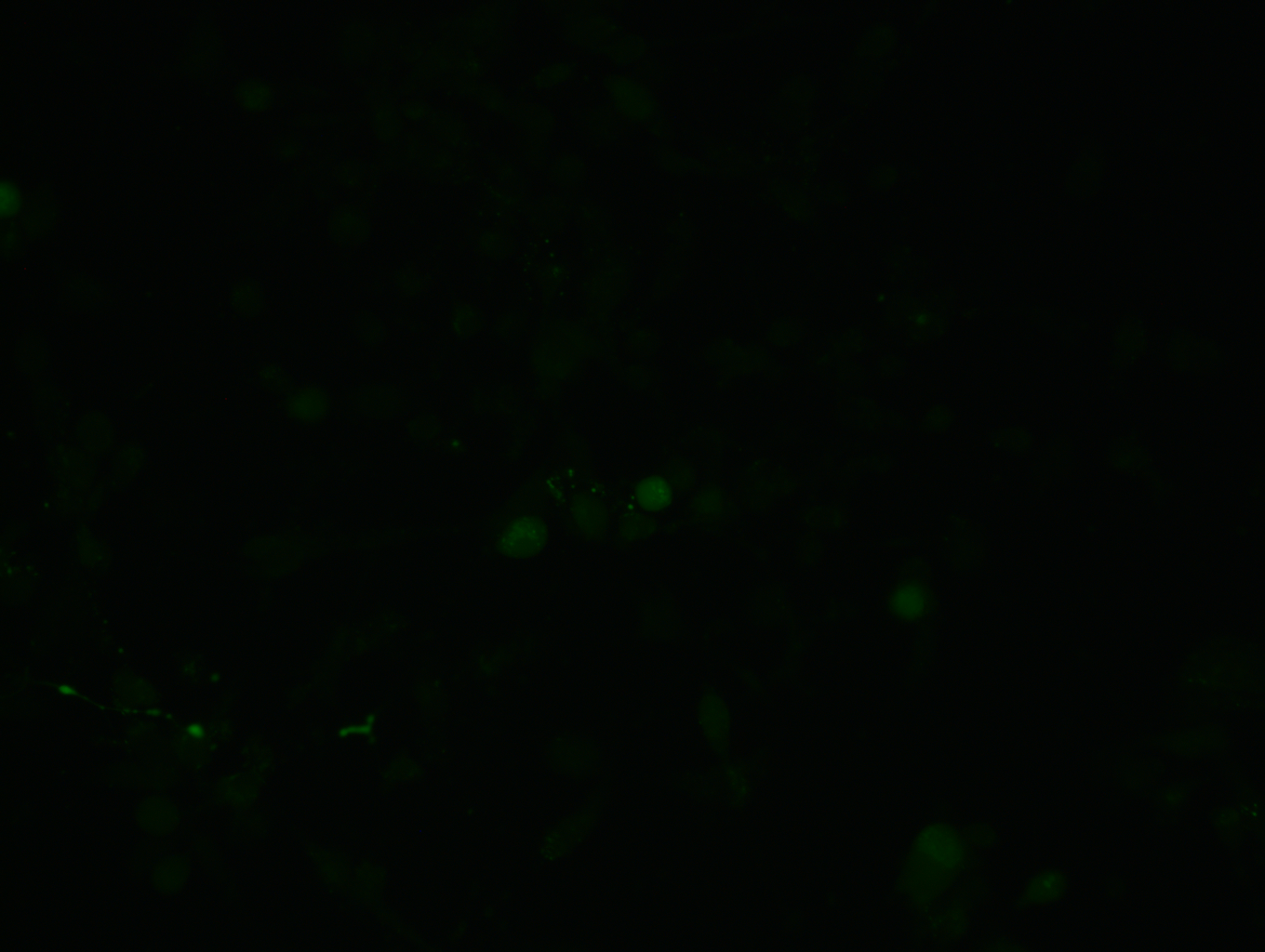

Supplement: S3 File — (ZIP) [file pone.0335890.s003.zip › Supporting Information3/Fig3/Fig3f/CTL/2.png]

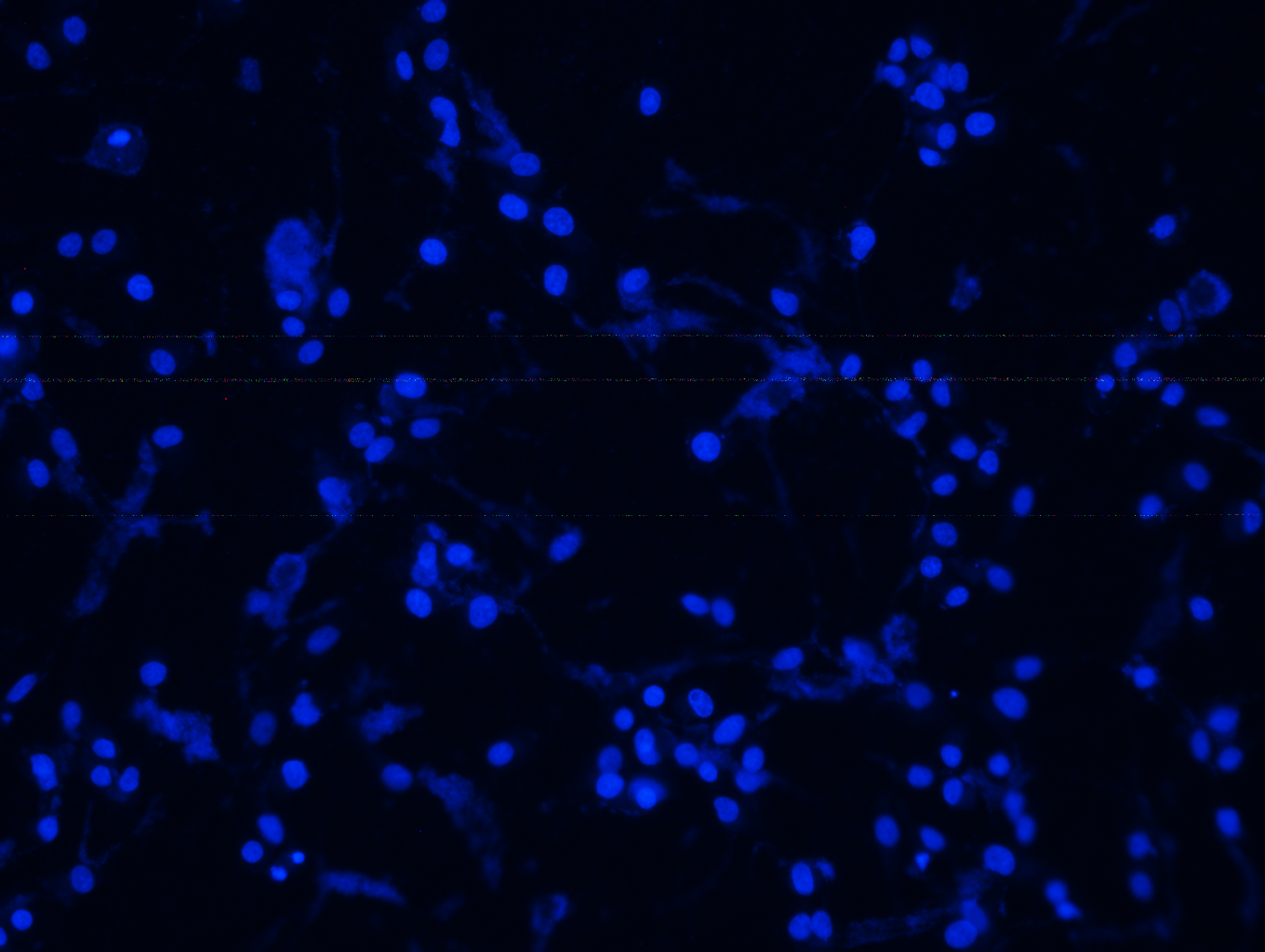

Supplement: S3 File — (ZIP) [file pone.0335890.s003.zip › Supporting Information3/Fig3/Fig3f/CTL/3..png]

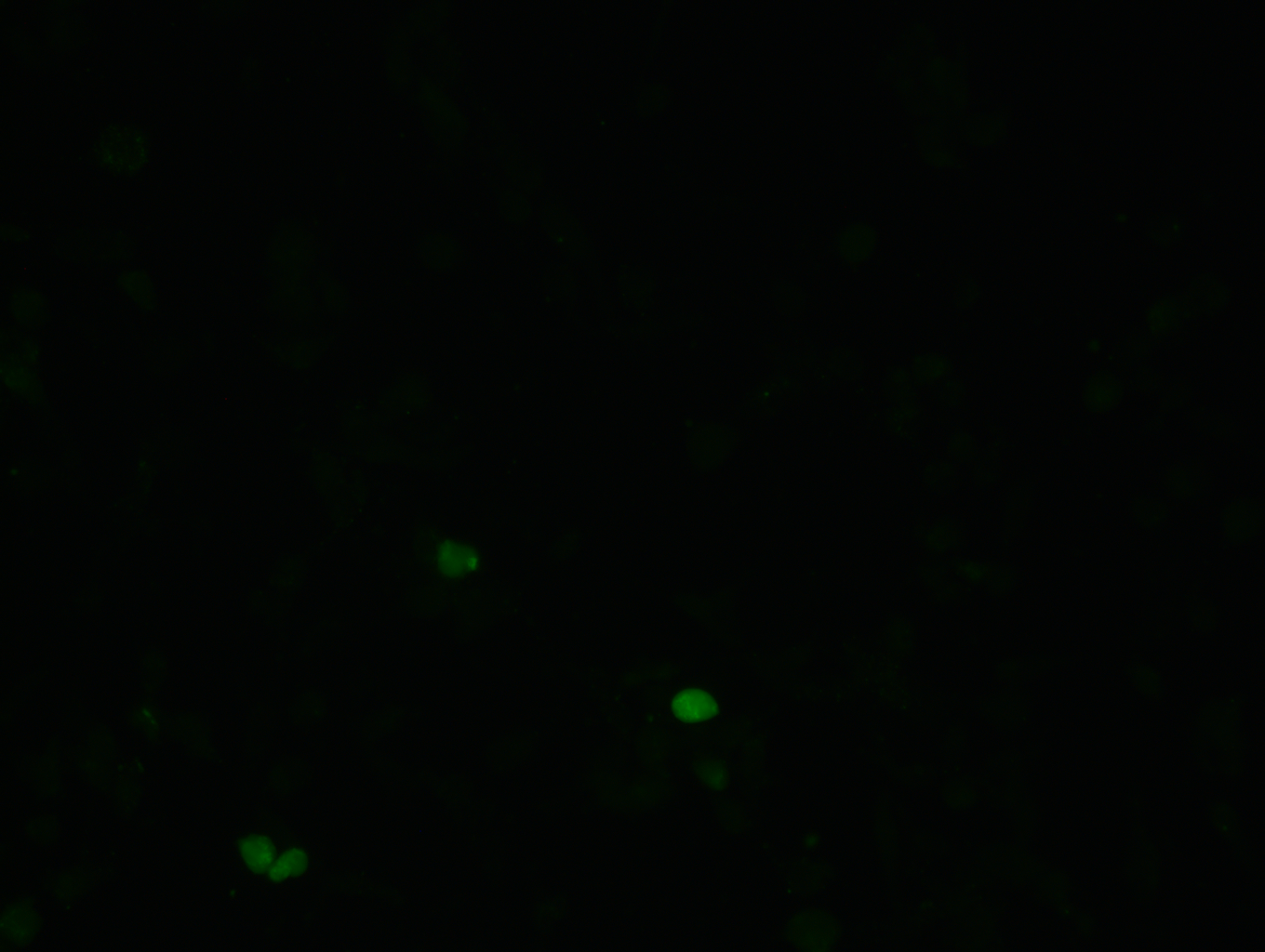

Supplement: S3 File — (ZIP) [file pone.0335890.s003.zip › Supporting Information3/Fig3/Fig3f/CTL/3.png]

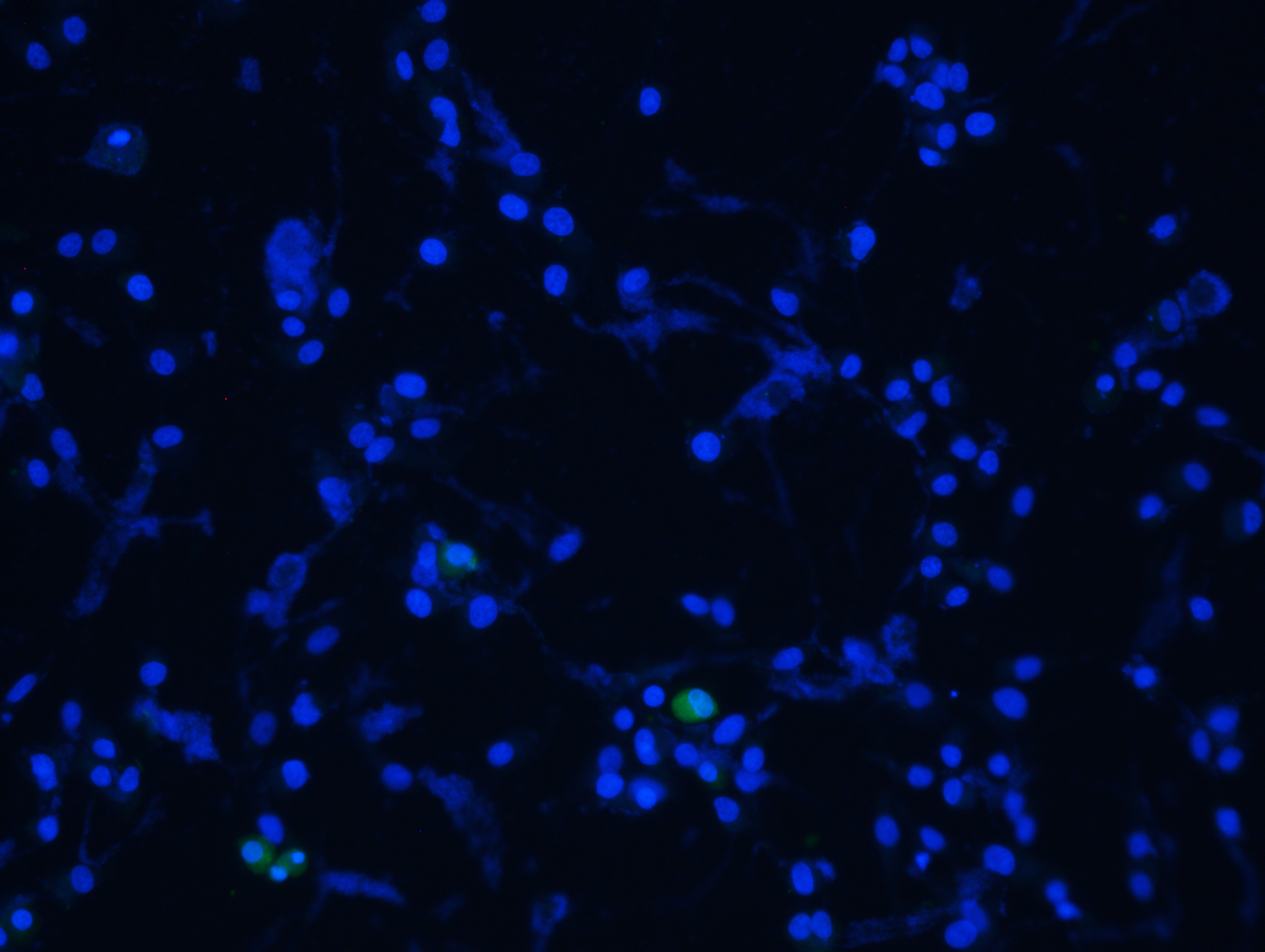

Supplement: S3 File — (ZIP) [file pone.0335890.s003.zip › Supporting Information3/Fig3/Fig3f/CTL/3OK.png]

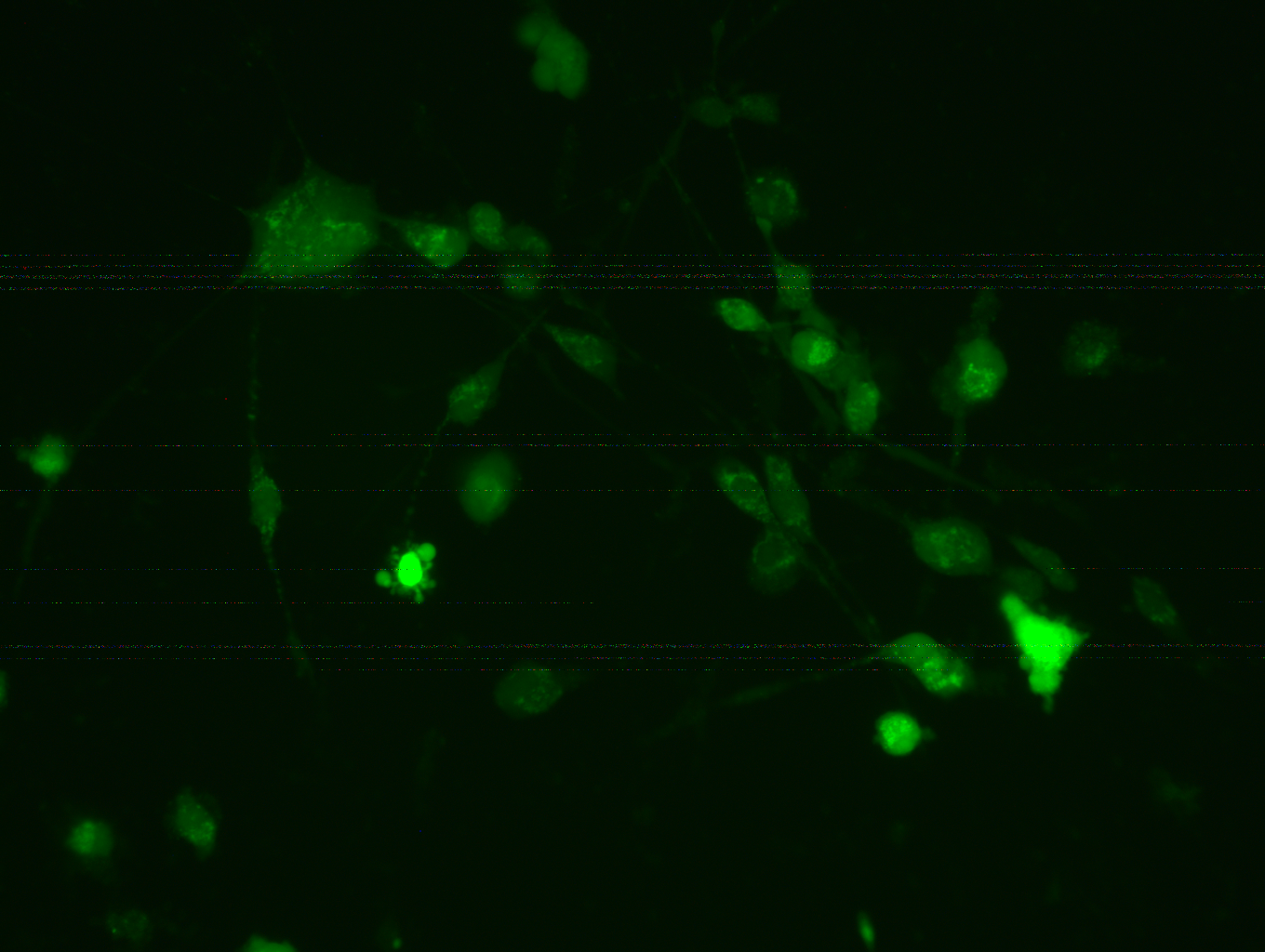

Supplement: S3 File — (ZIP) [file pone.0335890.s003.zip › Supporting Information3/Fig3/Fig3f/DBET6/1..png]

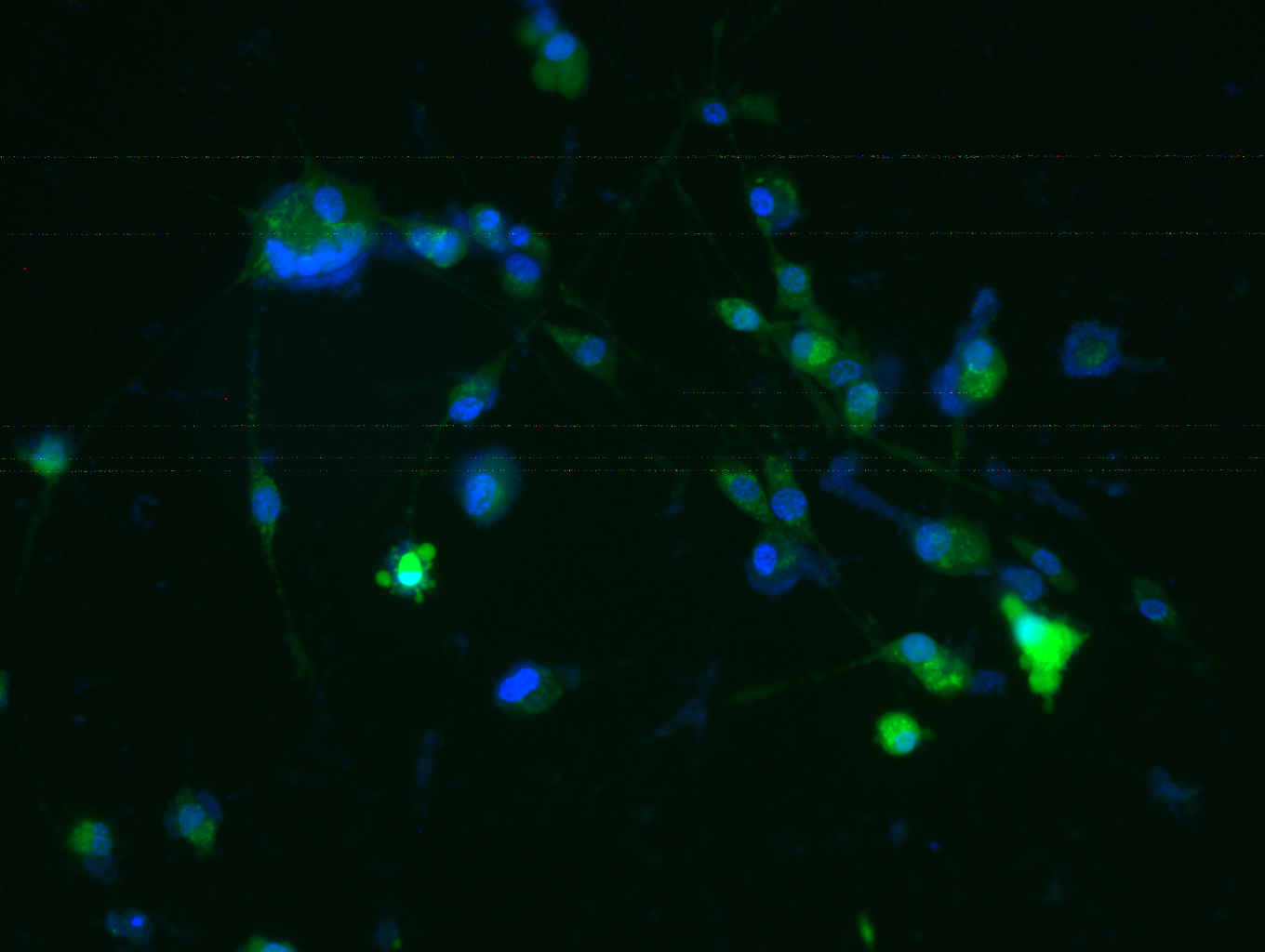

Supplement: S3 File — (ZIP) [file pone.0335890.s003.zip › Supporting Information3/Fig3/Fig3f/DBET6/1.ok.png]

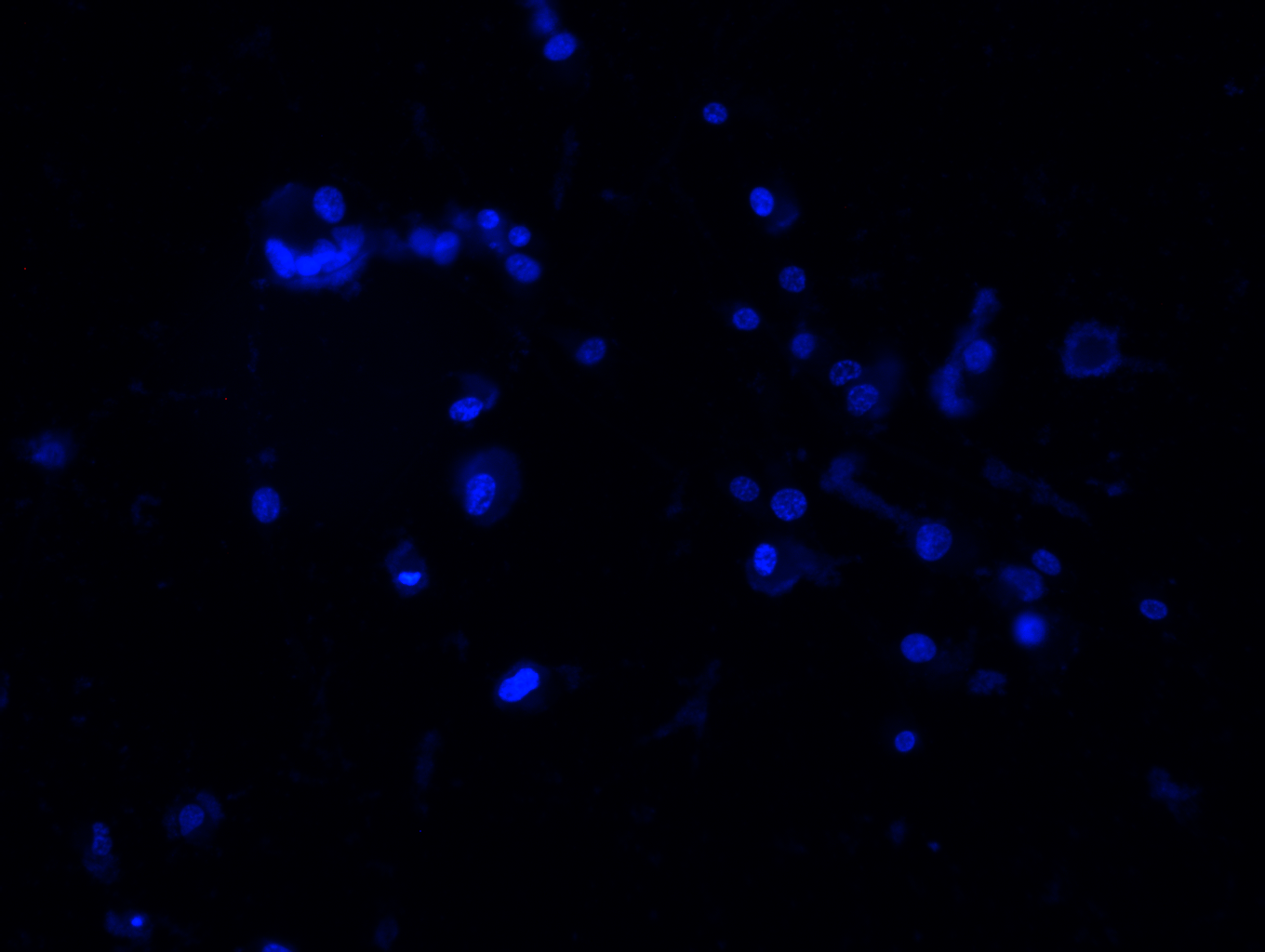

Supplement: S3 File — (ZIP) [file pone.0335890.s003.zip › Supporting Information3/Fig3/Fig3f/DBET6/1.png]

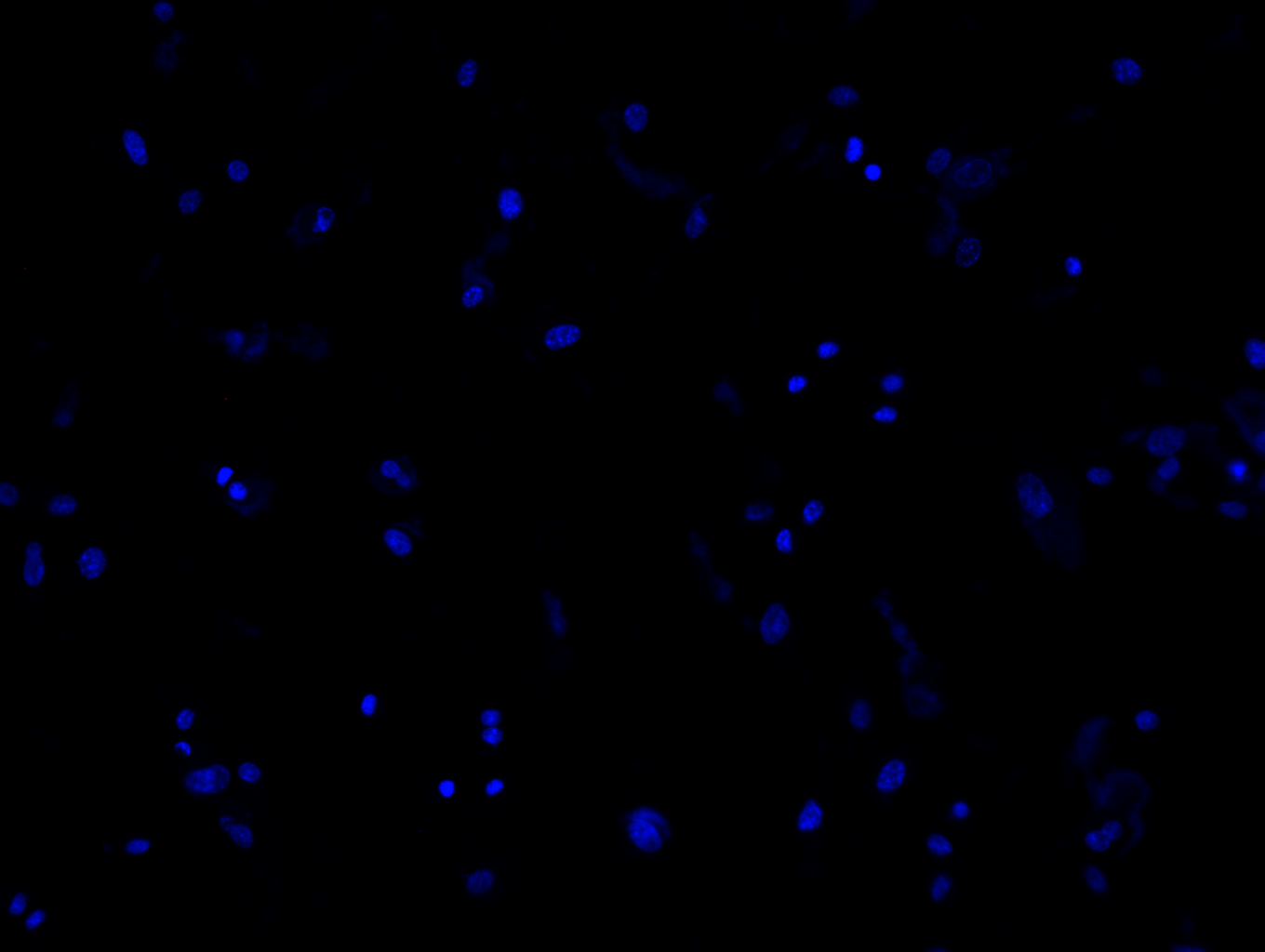

Supplement: S3 File — (ZIP) [file pone.0335890.s003.zip › Supporting Information3/Fig3/Fig3f/DBET6/2..png]

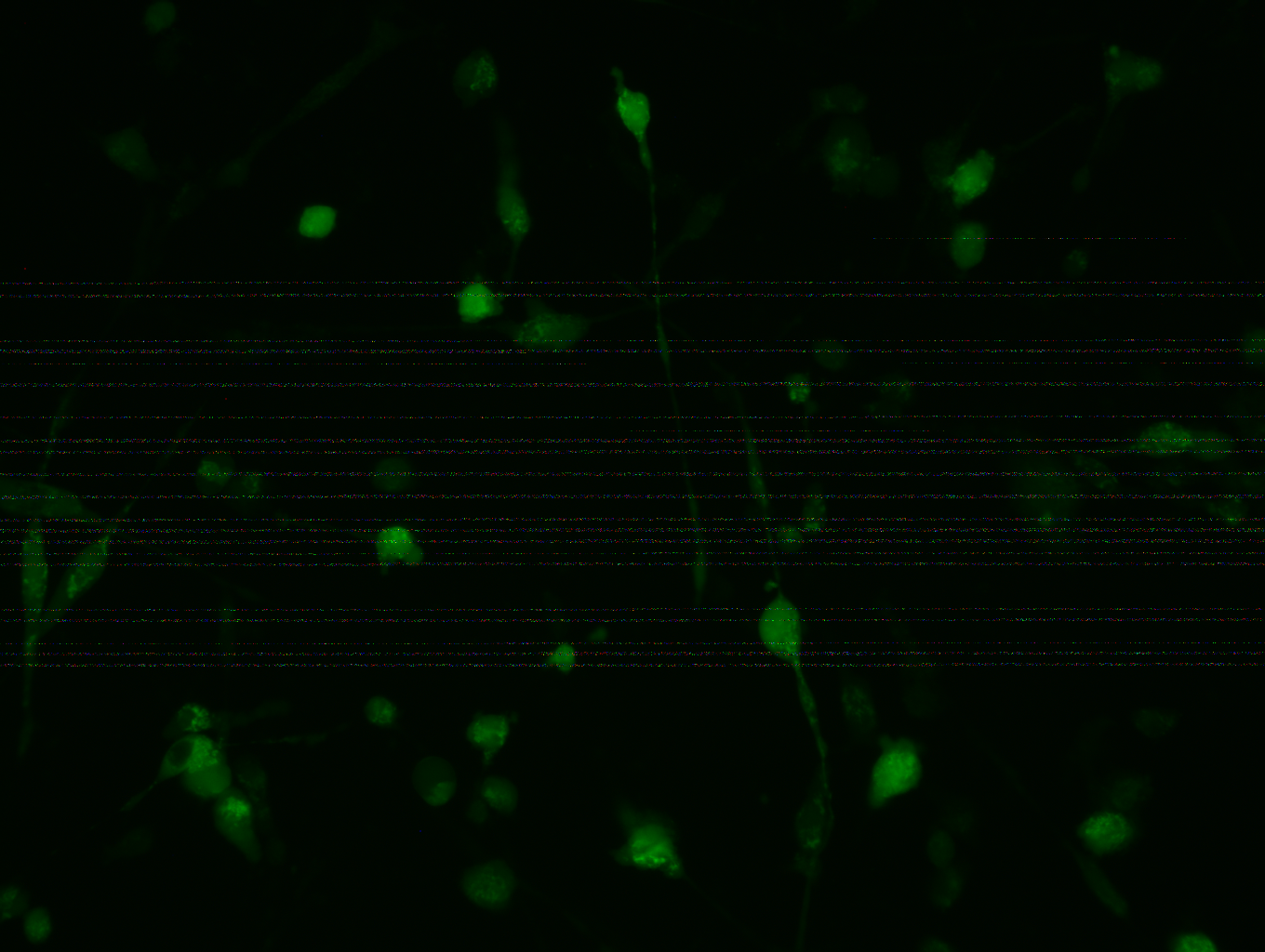

Supplement: S3 File — (ZIP) [file pone.0335890.s003.zip › Supporting Information3/Fig3/Fig3f/DBET6/2.png]

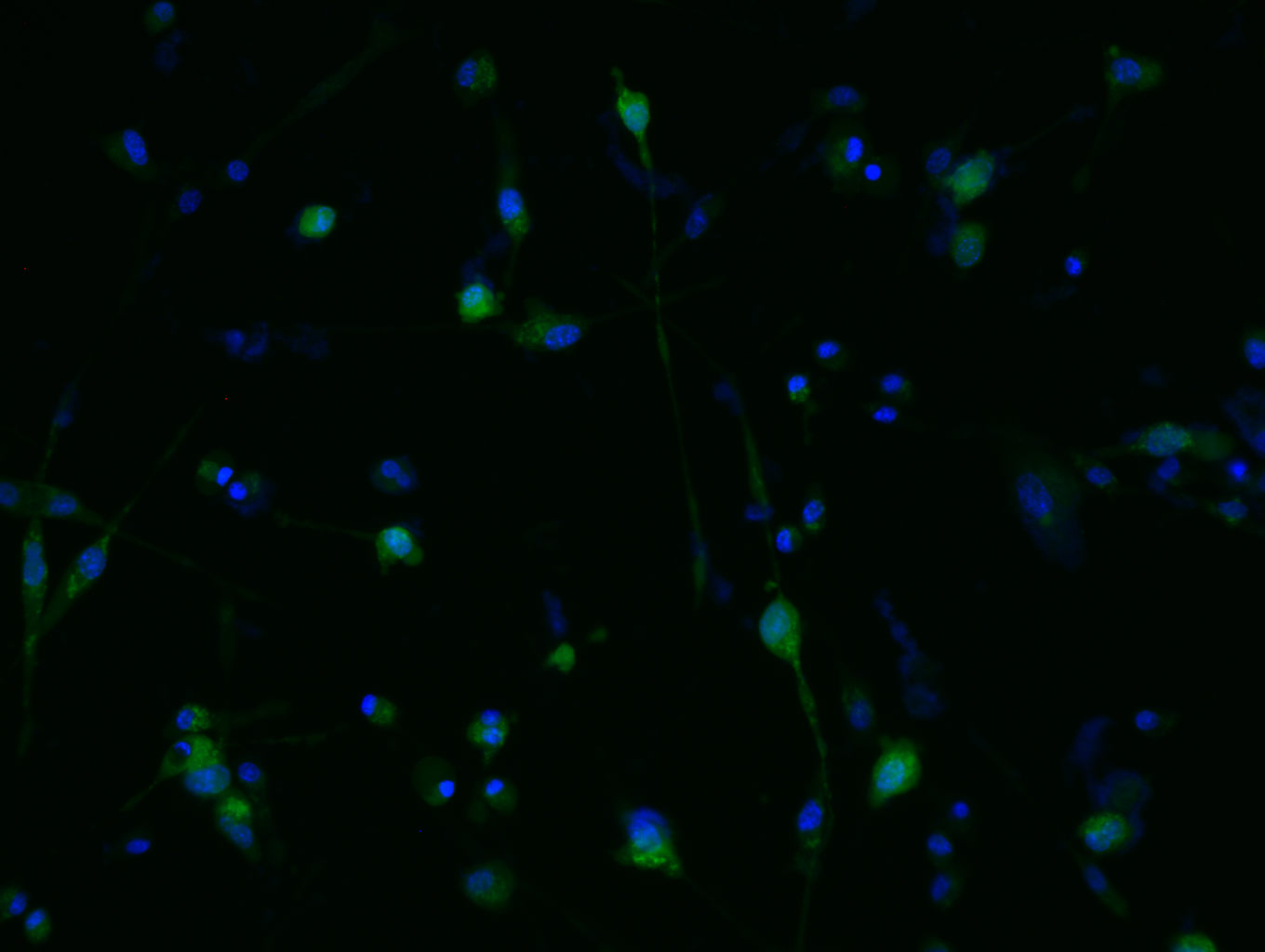

Supplement: S3 File — (ZIP) [file pone.0335890.s003.zip › Supporting Information3/Fig3/Fig3f/DBET6/2OK.png]

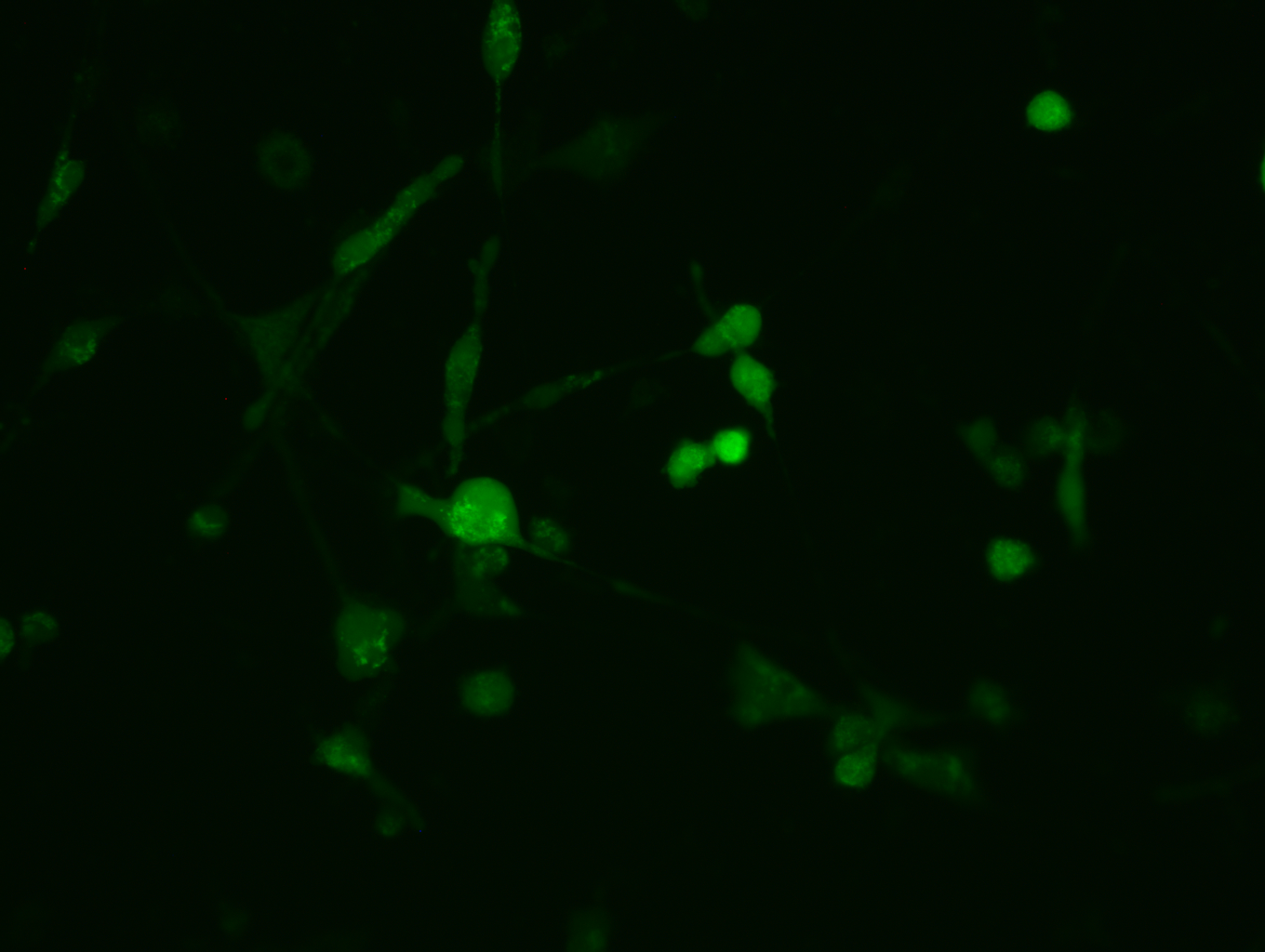

Supplement: S3 File — (ZIP) [file pone.0335890.s003.zip › Supporting Information3/Fig3/Fig3f/DBET6/3..png]

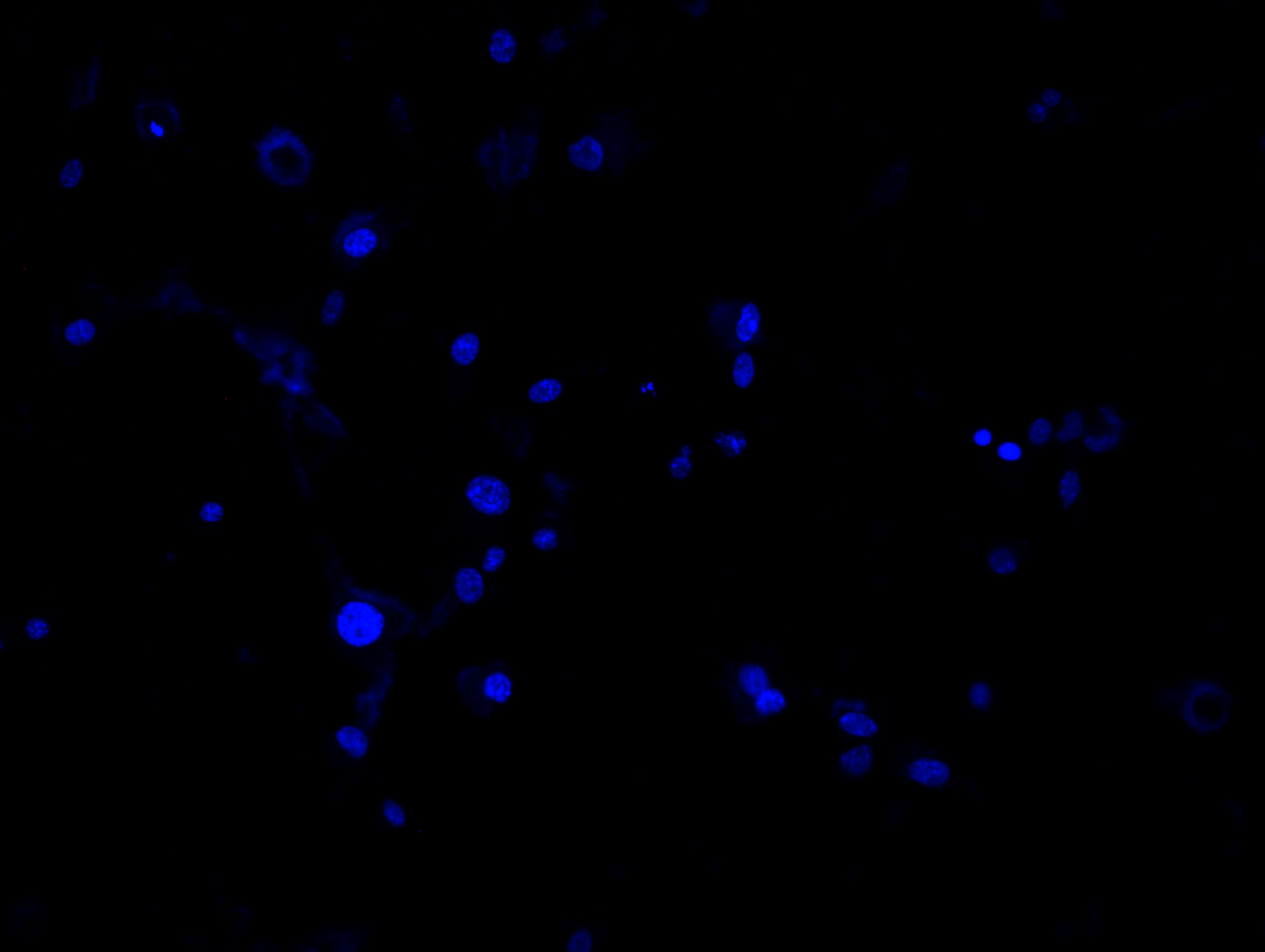

Supplement: S3 File — (ZIP) [file pone.0335890.s003.zip › Supporting Information3/Fig3/Fig3f/DBET6/3.png]

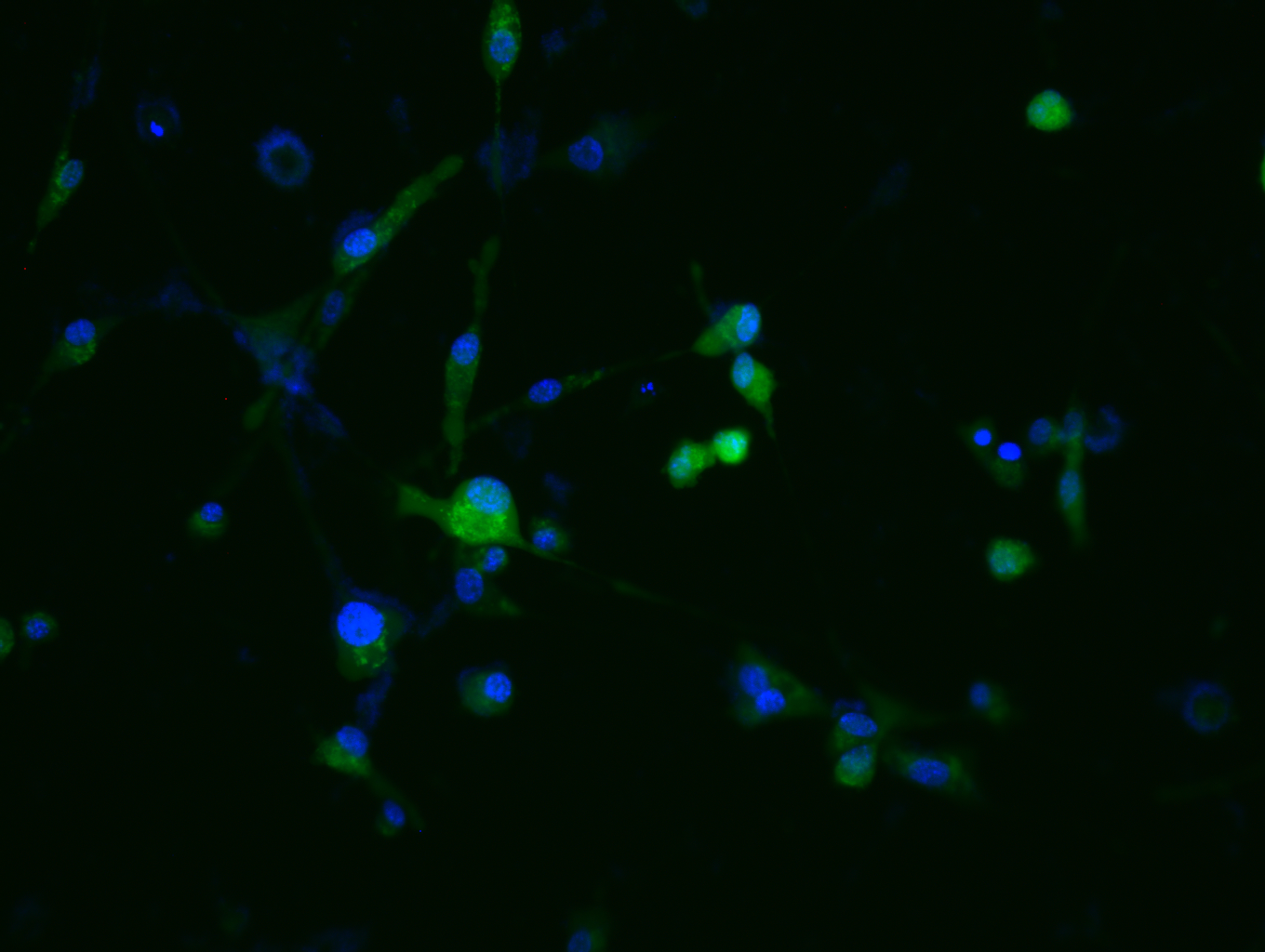

Supplement: S3 File — (ZIP) [file pone.0335890.s003.zip › Supporting Information3/Fig3/Fig3f/DBET6/3OK.png]

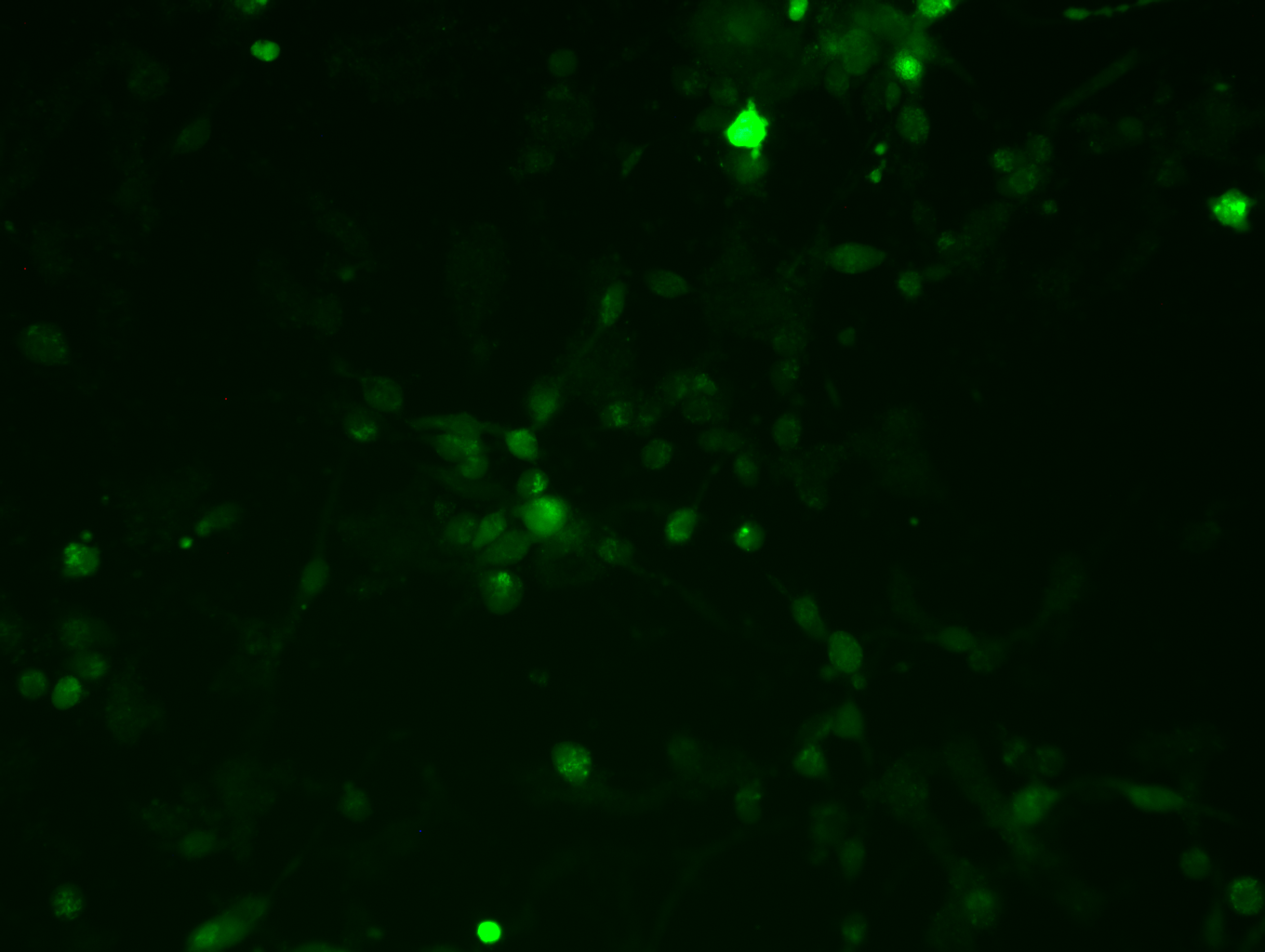

Supplement: S3 File — (ZIP) [file pone.0335890.s003.zip › Supporting Information3/Fig3/Fig3f/Exo-BSA@dbet6/1..png]

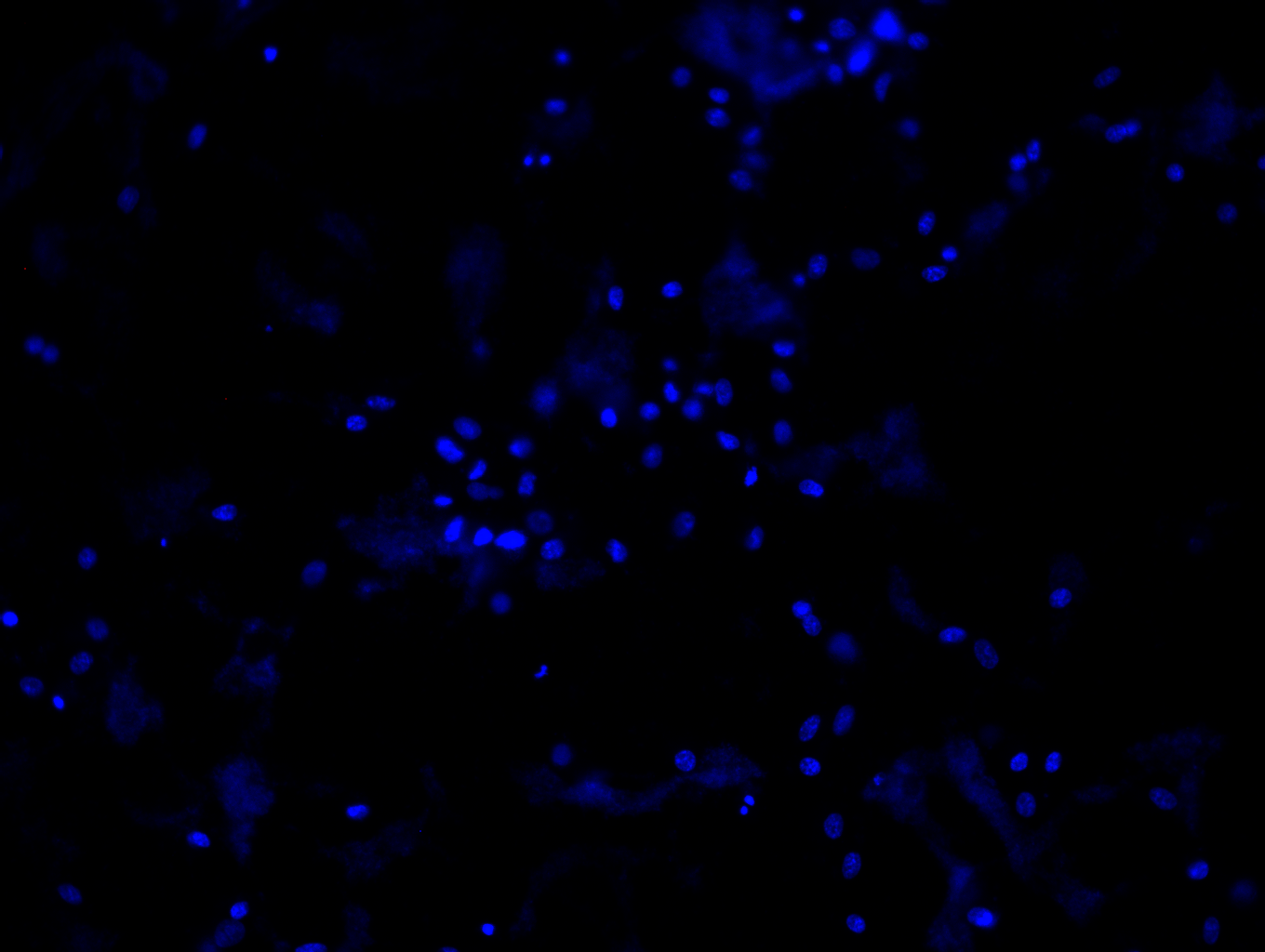

Supplement: S3 File — (ZIP) [file pone.0335890.s003.zip › Supporting Information3/Fig3/Fig3f/Exo-BSA@dbet6/1.png]

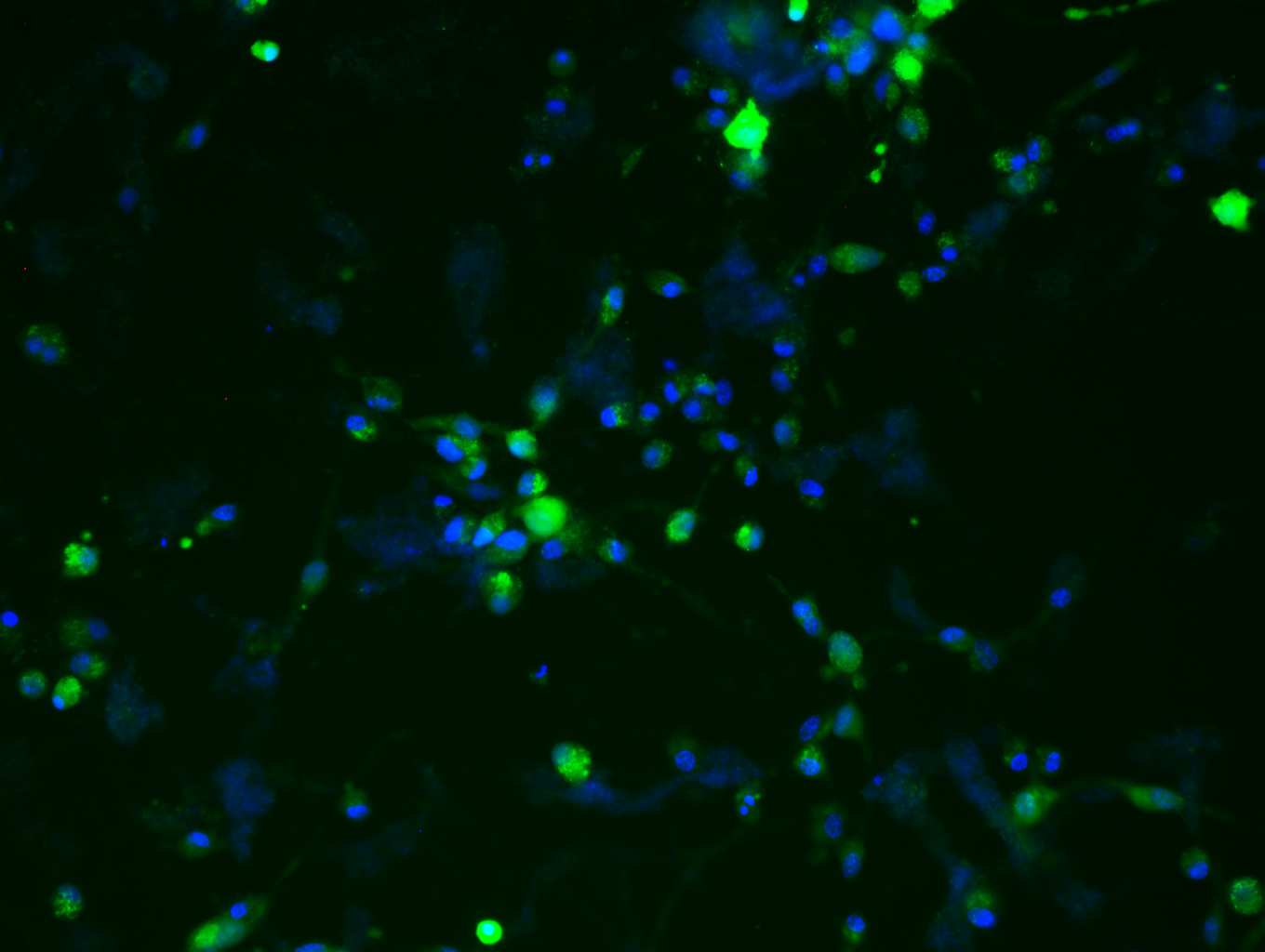

Supplement: S3 File — (ZIP) [file pone.0335890.s003.zip › Supporting Information3/Fig3/Fig3f/Exo-BSA@dbet6/1ok.png]

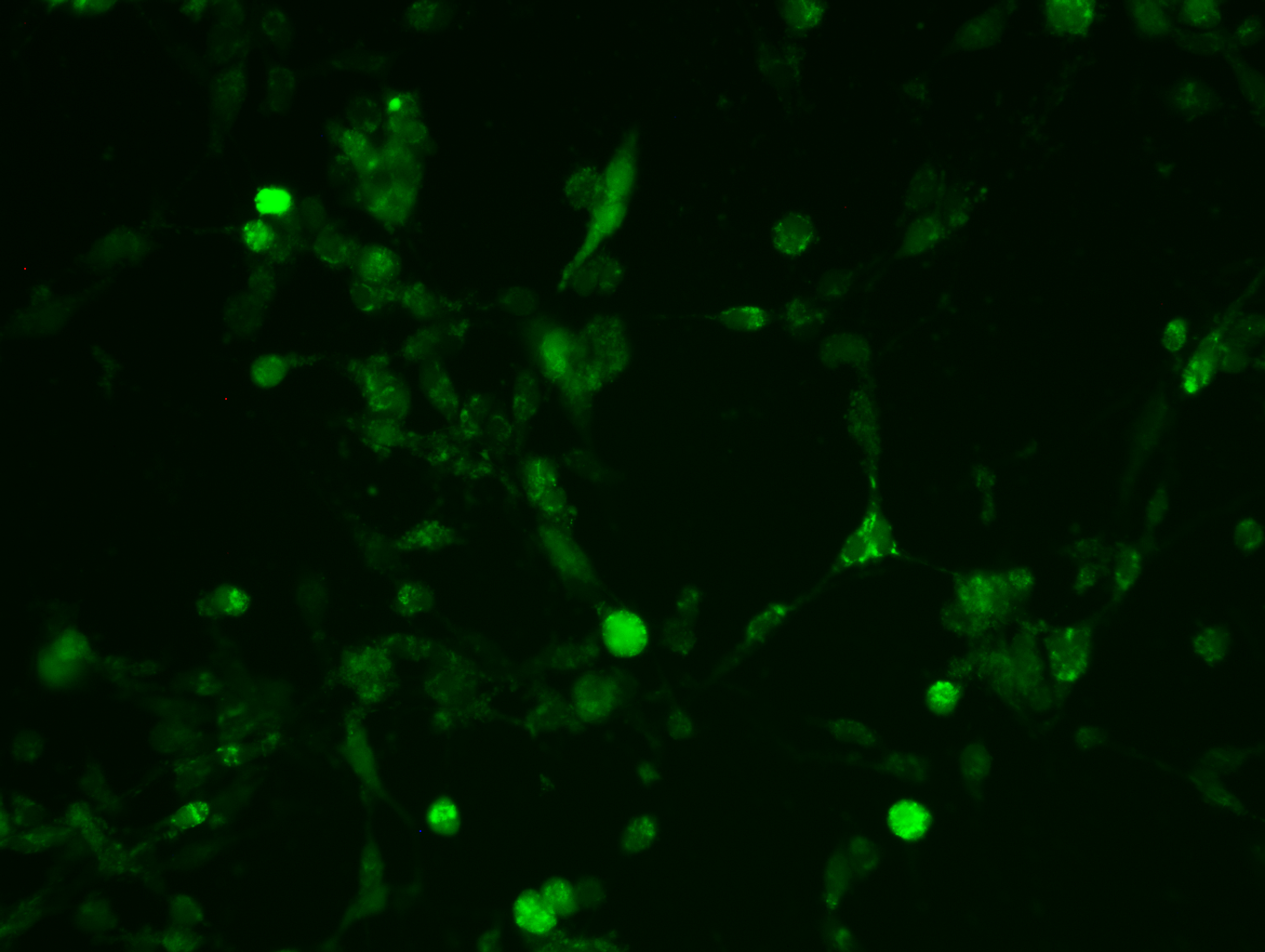

Supplement: S3 File — (ZIP) [file pone.0335890.s003.zip › Supporting Information3/Fig3/Fig3f/Exo-BSA@dbet6/2..png]

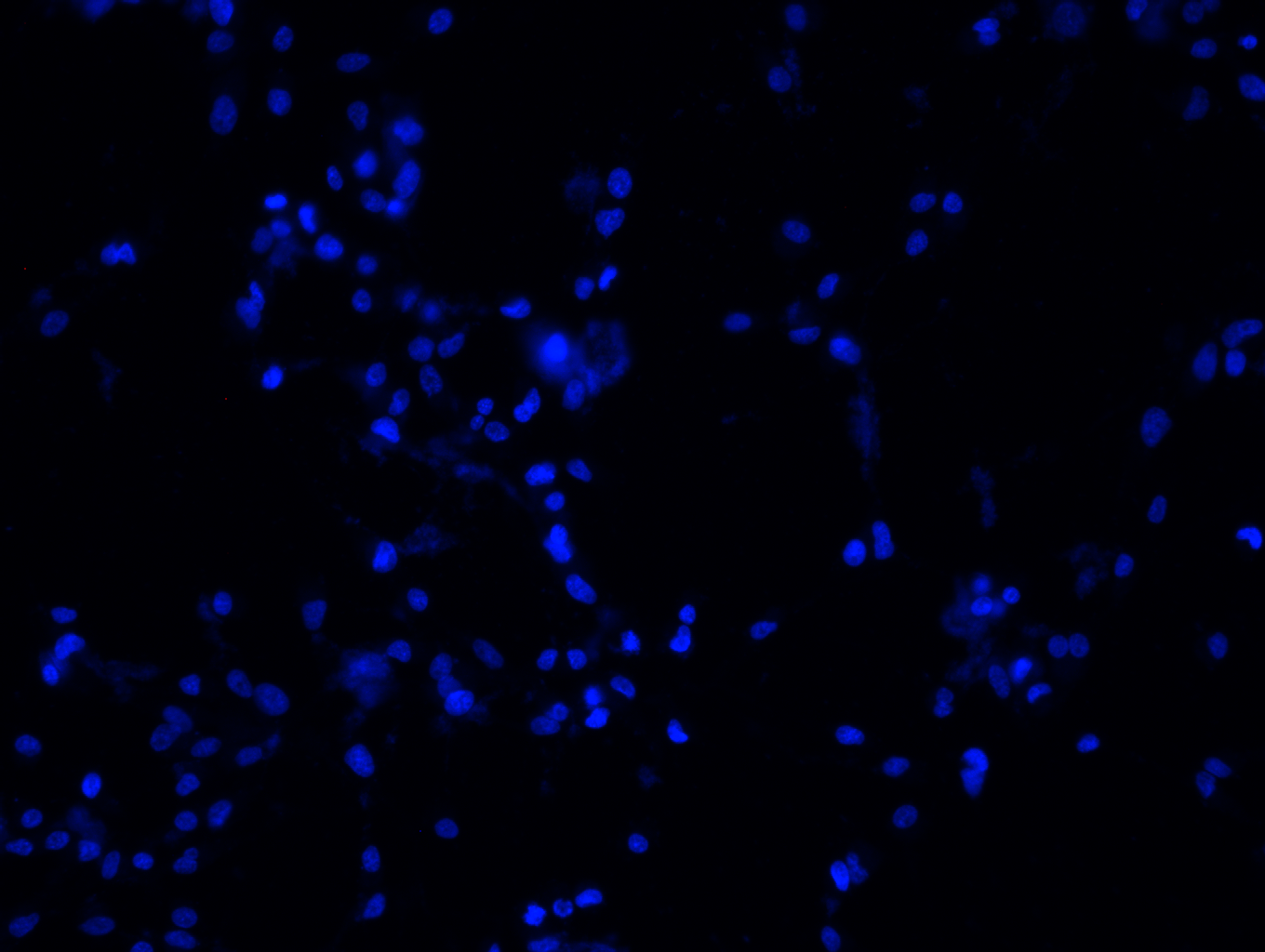

Supplement: S3 File — (ZIP) [file pone.0335890.s003.zip › Supporting Information3/Fig3/Fig3f/Exo-BSA@dbet6/2.png]

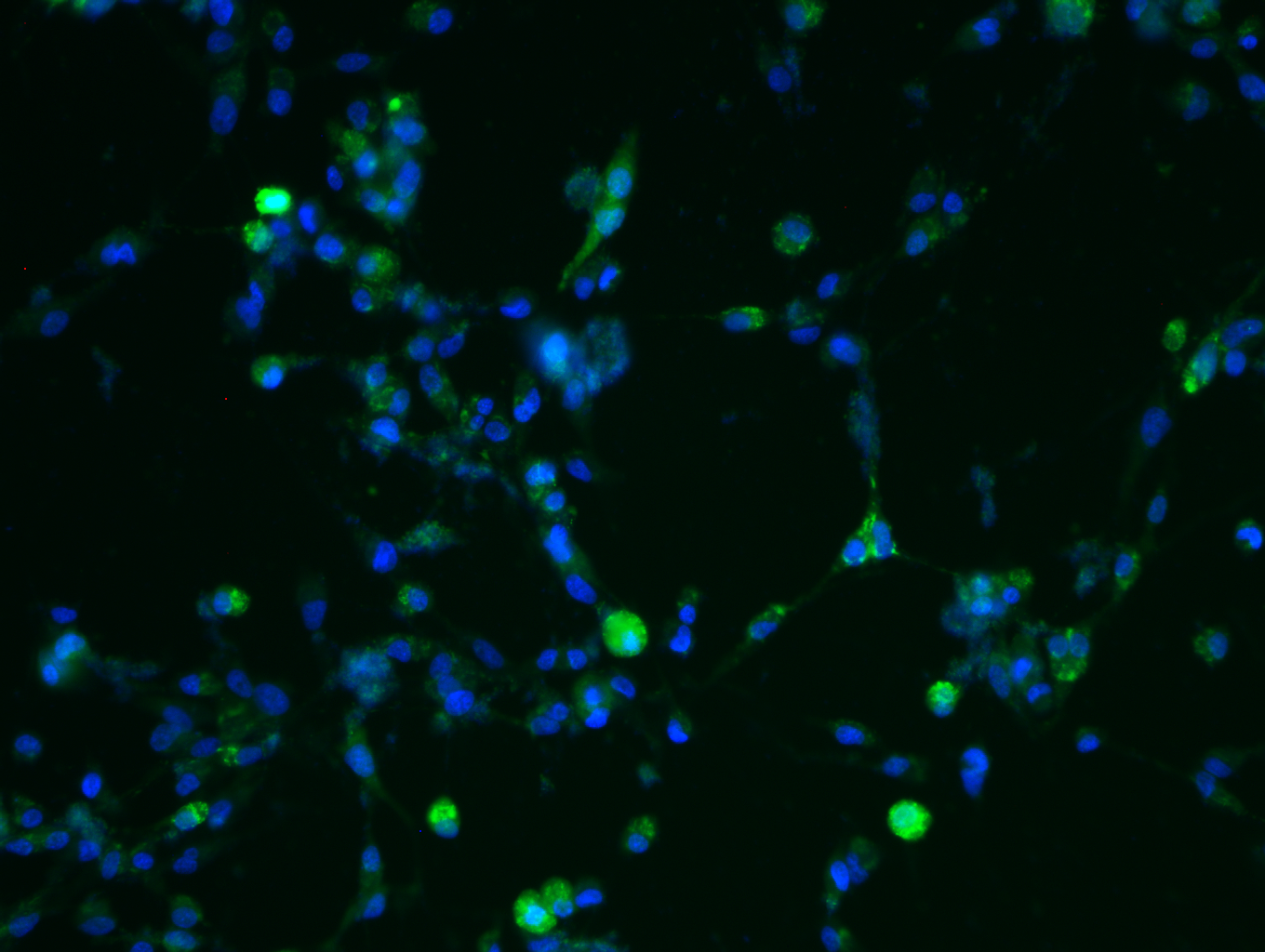

Supplement: S3 File — (ZIP) [file pone.0335890.s003.zip › Supporting Information3/Fig3/Fig3f/Exo-BSA@dbet6/2OK.png]

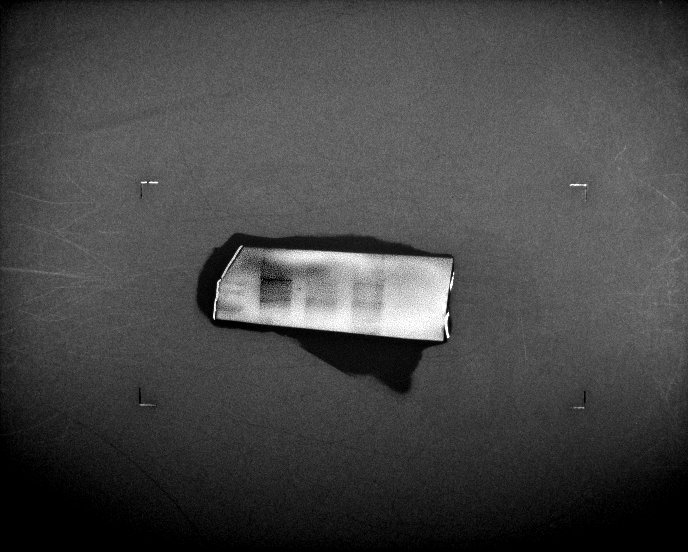

Supplement: S4 File — (ZIP) [file pone.0335890.s004.zip › Supporting Information3/Fig4/Fig4a/CDN BRD4.....png]

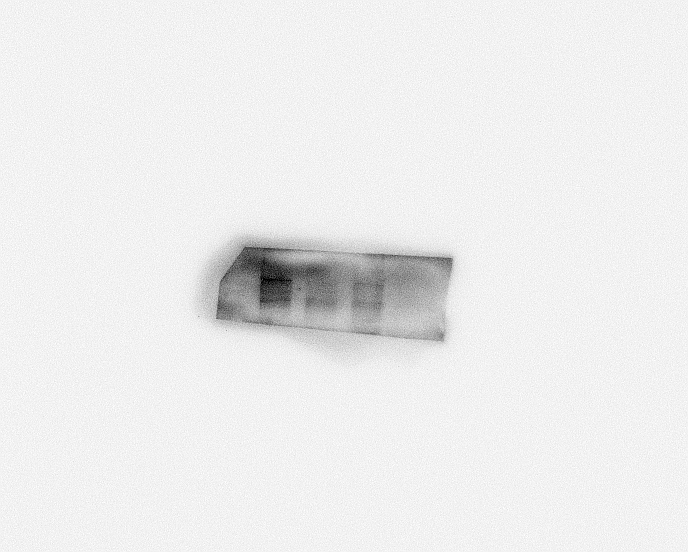

Supplement: S4 File — (ZIP) [file pone.0335890.s004.zip › Supporting Information3/Fig4/Fig4a/CDN BRD4..png]

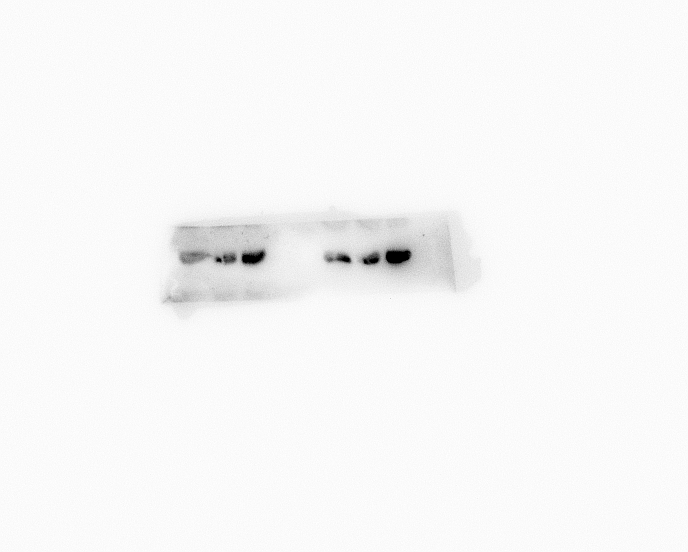

Supplement: S4 File — (ZIP) [file pone.0335890.s004.zip › Supporting Information3/Fig4/Fig4a/bax (2).png]

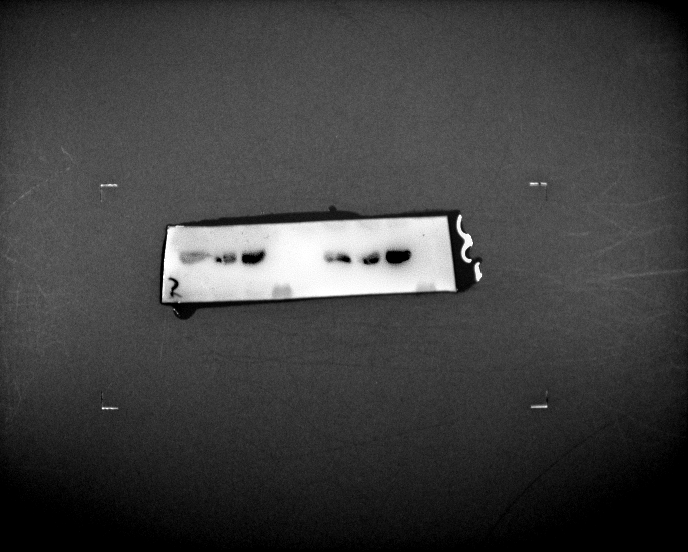

Supplement: S4 File — (ZIP) [file pone.0335890.s004.zip › Supporting Information3/Fig4/Fig4a/bax.. (2).png]

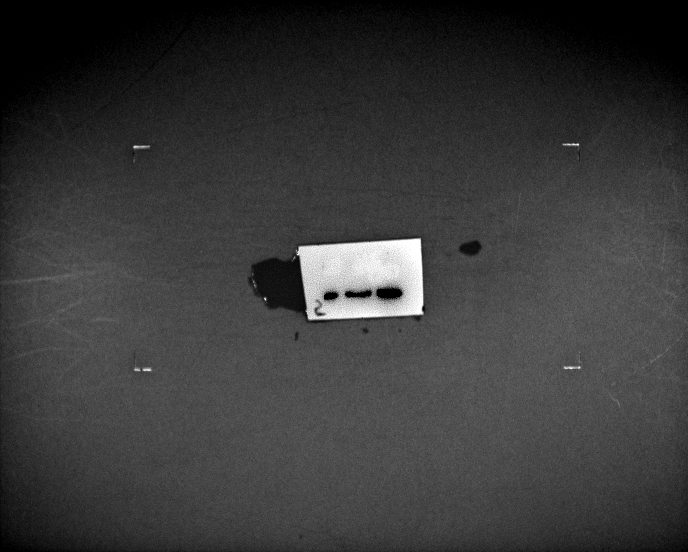

Supplement: S4 File — (ZIP) [file pone.0335890.s004.zip › Supporting Information3/Fig4/Fig4a/bax...png]

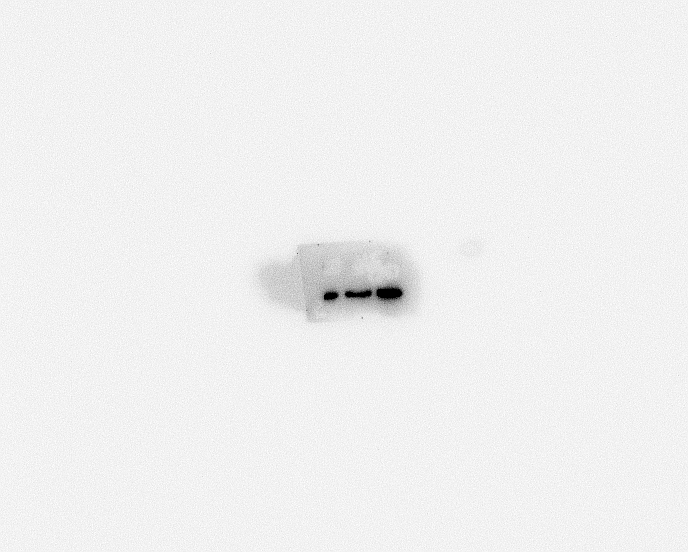

Supplement: S4 File — (ZIP) [file pone.0335890.s004.zip › Supporting Information3/Fig4/Fig4a/bax.png]

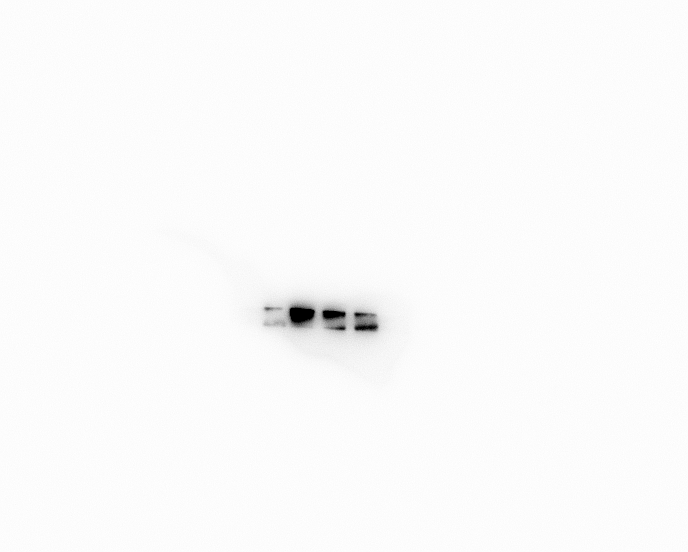

Supplement: S4 File — (ZIP) [file pone.0335890.s004.zip › Supporting Information3/Fig4/Fig4a/cmy cdn..png]

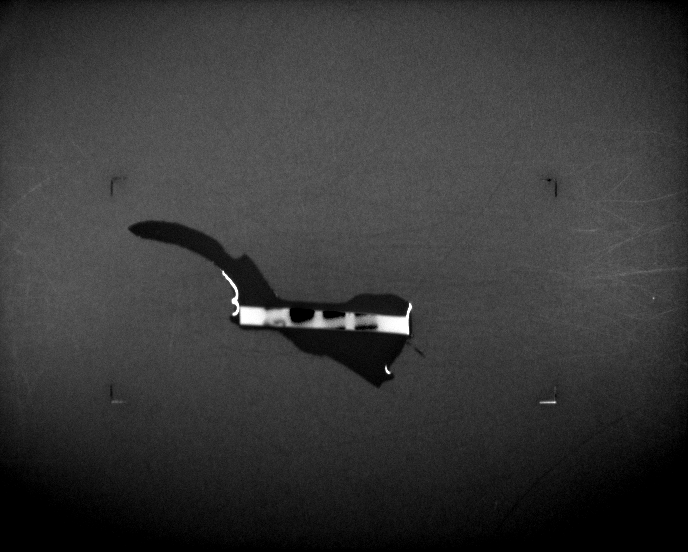

Supplement: S4 File — (ZIP) [file pone.0335890.s004.zip › Supporting Information3/Fig4/Fig4a/cmy cdn.png]

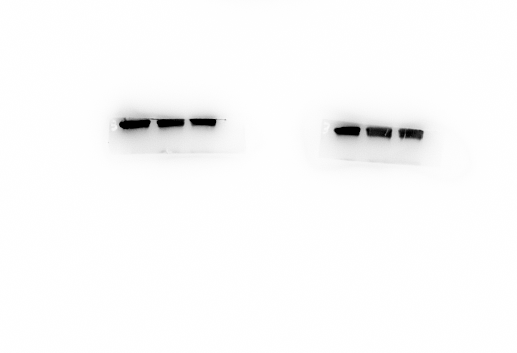

Supplement: S4 File — (ZIP) [file pone.0335890.s004.zip › Supporting Information3/Fig4/Fig4a/gapdh cdn..png]

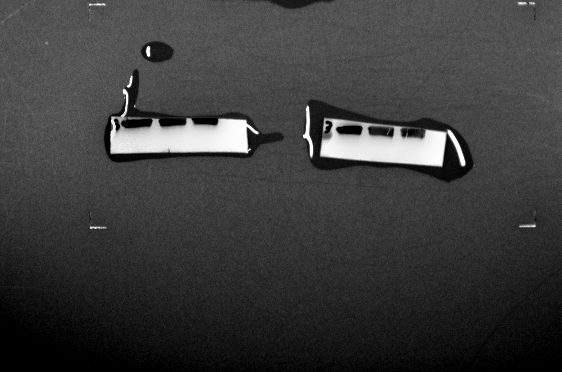

Supplement: S4 File — (ZIP) [file pone.0335890.s004.zip › Supporting Information3/Fig4/Fig4a/gapdh cdn.png]

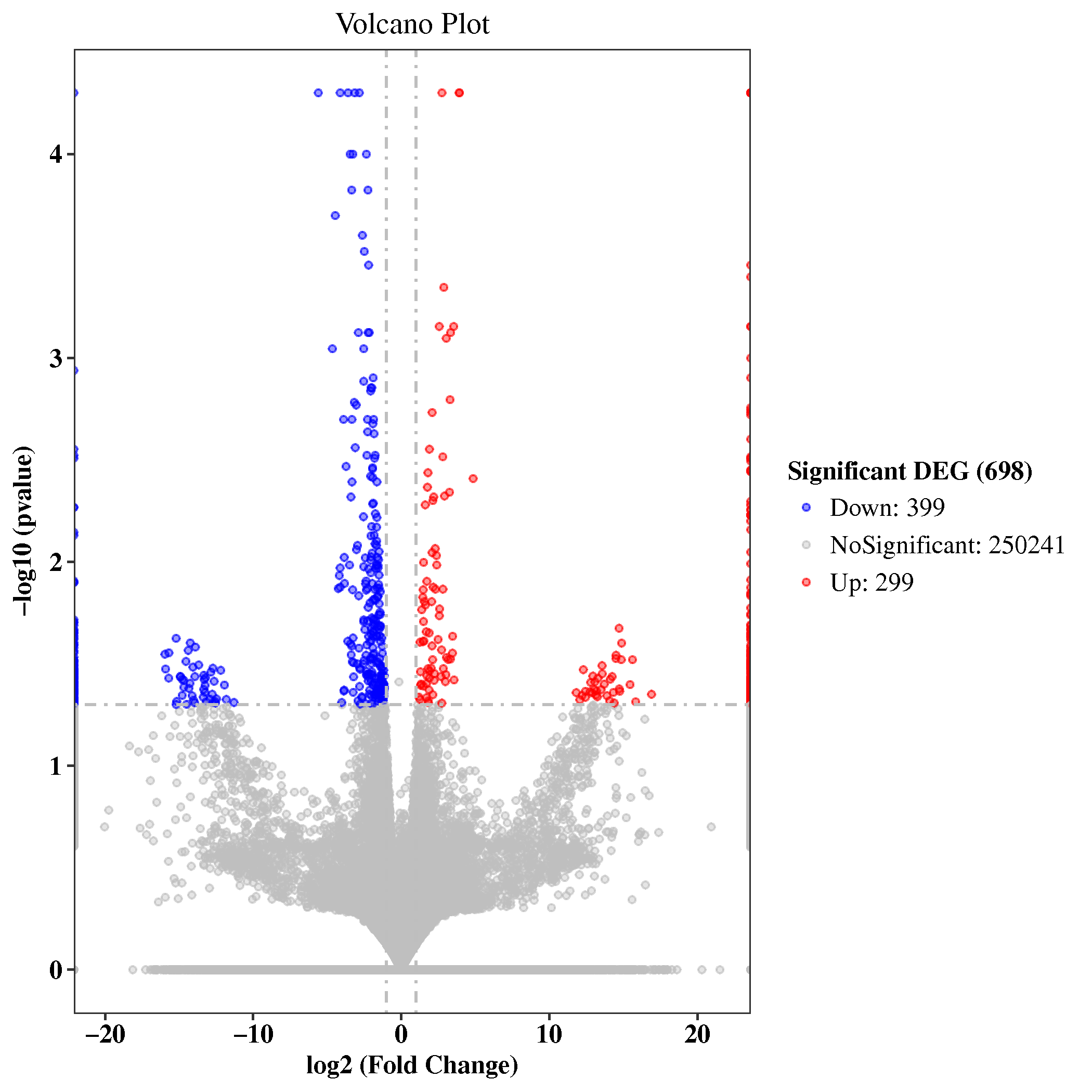

Supplement: S4 File — (ZIP) [file pone.0335890.s004.zip › Supporting Information3/Fig4/Fig4b/Group_CTL-VS-NPS_results.Volcano.png]

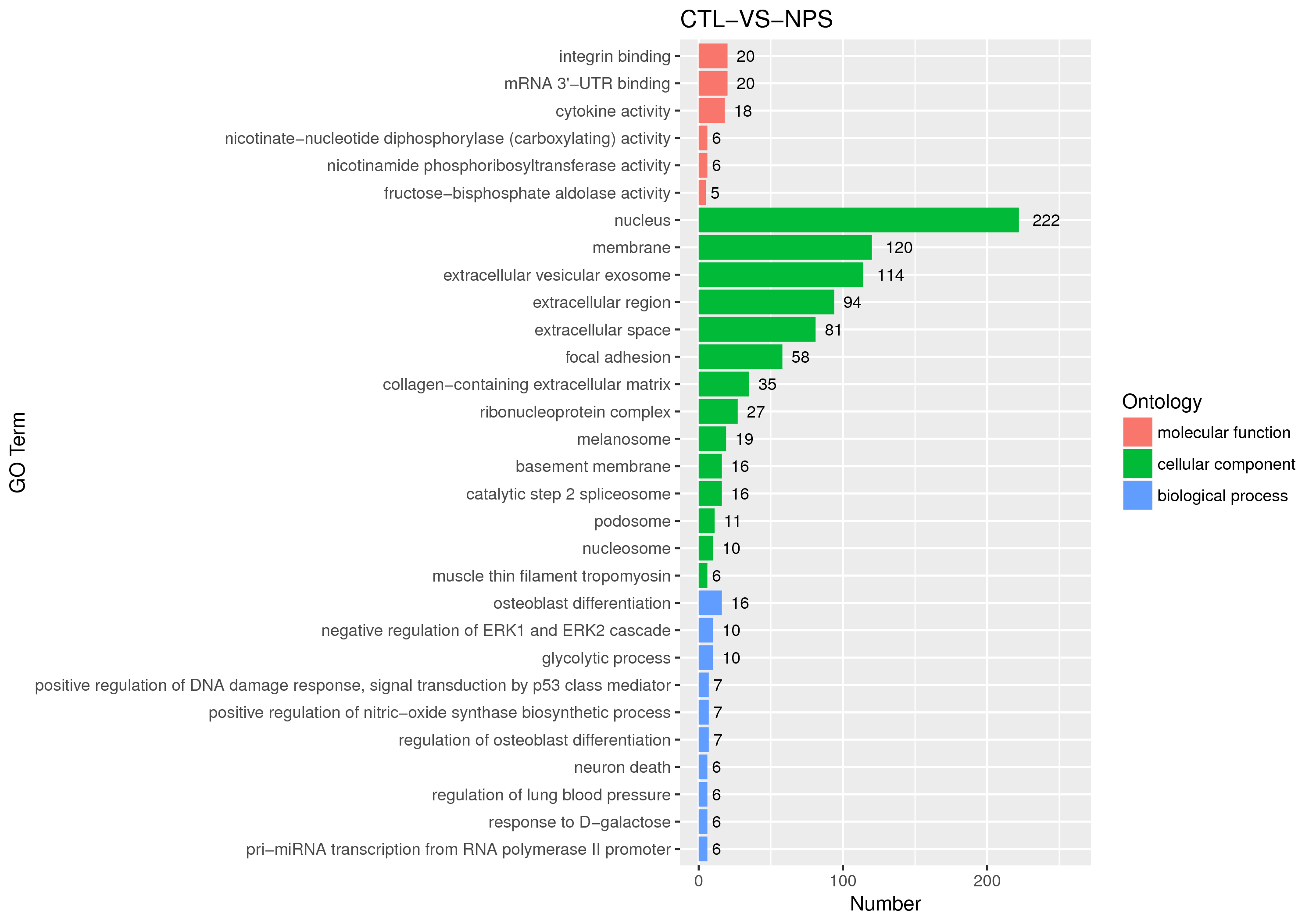

Supplement: S4 File — (ZIP) [file pone.0335890.s004.zip › Supporting Information3/Fig4/Fig4c/CTL-VS-NPS_GO_barplot.png]
