# Supplementary figures and images for: Exosome encapsulated albumin nanoparticles target delivery of DBET6 as a treatment for triple-negative breast cancer (part 2 of 2)
Source: PLoS One. 2026 Jan 12;21(1):e0335890. doi: 10.1371/journal.pone.0335890 (PMC12795375; doi:10.1371/journal.pone.0335890)

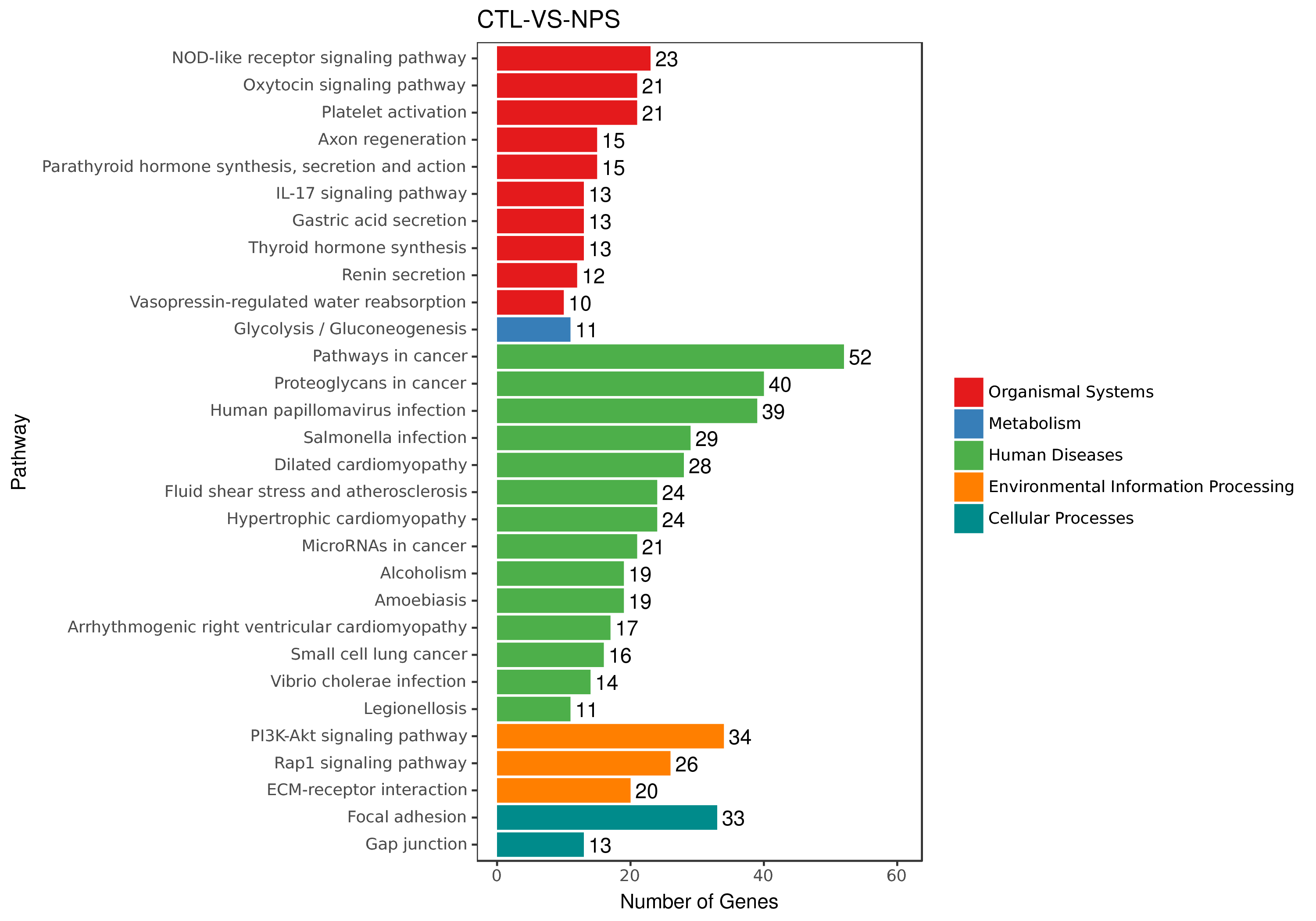

Supplement: S4 File — (ZIP) [file pone.0335890.s004.zip › Supporting Information3/Fig4/Fig4d/CTL-VS-NPS_PathwayBar.png]
