# Supplementary material for: Exosome encapsulated albumin nanoparticles target delivery of DBET6 as a treatment for triple-negative breast cancer
Source: PLoS One. 2026 Jan 12;21(1):e0335890. doi: 10.1371/journal.pone.0335890 (PMC12795375; doi:10.1371/journal.pone.0335890)
Supplement: S1 Raw images — (PDF) [file pone.0335890.s005.pdf]

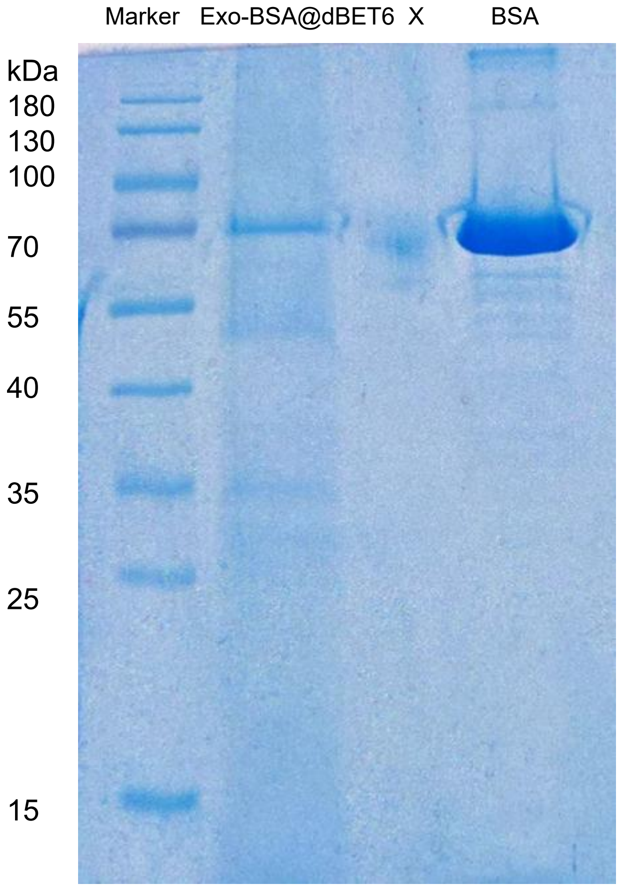

Coomasie Blue-stained gel | Image Source: Bio-Rad ChemiDoc MP | Corresponding Panel: Figure 1c

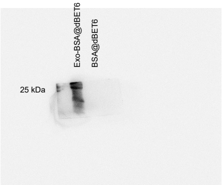

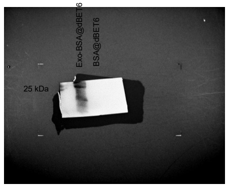

Western Blot: CD9 | Image Source: Bio-Rad ChemiDoc MP | Corresponding Panel: Figure 1e

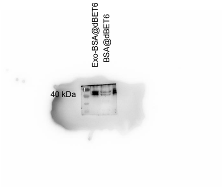

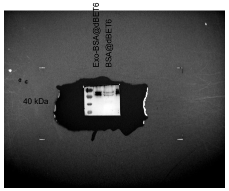

Western Blot: CD63 | Image Source: Bio-Rad ChemiDoc MP | Corresponding Panel: Figure 1e

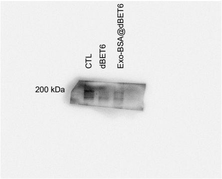

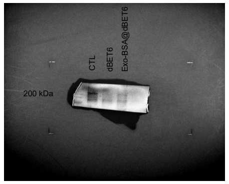

Western Blot: BRD4 | Image Source: Bio-Rad ChemiDoc MP | Corresponding Panel: Figure 4a

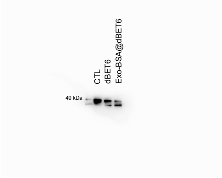

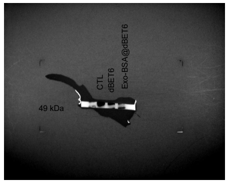

Western Blot: C-Myc | Image Source: Bio-Rad ChemiDoc MP | Corresponding Panel: Figure 4a

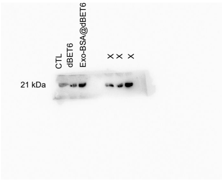

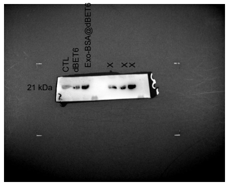

Western Blot: Bax | Image Source: Bio-Rad ChemiDoc MP | Corresponding Panel: Figure 4a

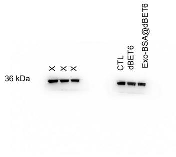

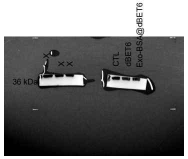

Western Blot: GADPH | Image Source: Bio-Rad ChemiDoc MP | Corresponding Panel: Figure 4a
