# Supplementary material for: Exosome encapsulated albumin nanoparticles target delivery of DBET6 as a treatment for triple-negative breast cancer
Source: PLoS One. 2026 Jan 12;21(1):e0335890. doi: 10.1371/journal.pone.0335890 (PMC12795375; doi:10.1371/journal.pone.0335890)
Supplement: Attachment — Submitted filename: General Assessment_Varamini.pdf [file pone.0335890.s006.pdf]

Reviewer Comments on Manuscript PONE-D-25-29407
Title: *Exosome Encapsulated Albumin Nanoparticles Target Delivery of DBET6 as a Treatment for Triple-negative Breast Cancer*
Dear Editors,
Thank you for the opportunity to review this manuscript. The authors describe the development of exosome-encapsulated albumin nanoparticles loaded with dBET6, a PROTAC targeting BRD4, for potential treatment of triple-negative breast cancer (TNBC). The study aligns with the journal's scope in concept, but suffers from major methodological, analytical, and reporting deficiencies that preclude its publication in its current form.

Major Comments:

1. **Inadequate Nanoparticle Characterisation:**
   - The manuscript includes only basic nanoparticle characterisation (TEM, DLS, SDS-PAGE, and Western blot for exosome markers). However, essential physicochemical parameters — such as **zeta potential**, **polydispersity index (PDI)**, **drug loading**, **encapsulation efficiency**, and **nanoparticle yield** — are not reported.
   - Critically, **no drug release studies** are provided, which is a fundamental requirement for any study proposing a drug delivery system. Without drug release kinetics, the proposed benefit of sustained or targeted delivery remains entirely unverified.
2. **Lack of Structural Confirmation of the Drug–Carrier Complex:**
   - The manuscript lacks analytical evidence confirming the successful incorporation and structural integrity of dBET6 within the nanoparticles. Given that dBET6 is a small molecule PROTAC, its presence and stability in the system should be confirmed using appropriate analytical methods such as **mass spectrometry**, **HPLC**, or **UV absorbance**. This is especially critical when proposing a mechanism reliant on intracellular BRD4 degradation.
3. **No Description of Albumin Nanoparticle Preparation:**
   - It is unclear whether the albumin nanoparticles were prepared by the authors or sourced commercially. If prepared in-house, no methodology or optimisation strategy is described, making the system irreproducible and scientifically unverifiable. This is a major concern for a research article aiming to establish a novel delivery platform.
4. **Limited Mechanistic and Functional Validation:**
   - Although the manuscript reports in vitro uptake and cytotoxicity assays, the data are largely descriptive. There is **no quantitative statistical analysis** (e.g., error bars, p-values, replicate numbers), nor are proper negative or vehicle controls described.
   - The transcriptomic data presented are not validated through downstream experiments such as **qPCR**, **pathway inhibition**, or **gene silencing**, which significantly limits the depth of mechanistic insight.
5. **Absence of Stability and In Vivo Data:**
   - No stability data are provided (e.g., nanoparticle integrity in serum or under physiological pH), which is necessary even in the absence of

animal work. The in vivo limitation is acknowledged, but the complete omission of **in vitro stability studies** further weakens the case for translational relevance.

Additional Comments:

- **Incorrect Referencing in the Introduction:**
  The epidemiological statistics quoted regarding breast cancer incidence do not match the cited source and should be corrected.
- **Poor Paragraph Transitions:**
  Several sections in the introduction lack logical flow, particularly the abrupt shift from disease context to PROTACs and nanoparticle systems.
- **Unclear Exosome Origin:**
  The source of the exosomes is ambiguously reported. Whether they are derived from TNBC cells, camel milk (as mentioned in a cited study), or another origin is not specified. This significantly impacts the interpretation of the targeting efficacy.
- **Language and Formatting Issues:**
  The manuscript requires extensive English language editing. Inconsistent terminology (e.g., "dbet6" vs "dBET6") and multiple grammatical errors detract from clarity.

Recommendation: Rejection
Despite a conceptually interesting approach, the manuscript lacks the fundamental scientific and technical elements required for publication. The absence of core nanoparticle characterisation, drug release data, structural validation of the delivery system, and reproducible formulation methodology are critical shortcomings. Furthermore, poor statistical reporting and limited mechanistic validation further weaken the manuscript.

I therefore recommend **rejection** of this manuscript.
Kind regards,

Pegah Varamini

--
Dr Pegah Varamini | PharmD, PhD, PhD
Senior Lecturer in Pharmacy and Pharmacology
Head of Breast Cancer Targeting & Drug Delivery (BCTDD) Laboratory
Sydney School of Pharmacy, Faculty of Medicine and Health
Co-Chair of NanoPharma within NanoHealth Initiative

The University of Sydney Nano Institute
