# Supplementary material for: Exosome encapsulated albumin nanoparticles target delivery of DBET6 as a treatment for triple-negative breast cancer
Source: PLoS One. 2026 Jan 12;21(1):e0335890. doi: 10.1371/journal.pone.0335890 (PMC12795375; doi:10.1371/journal.pone.0335890)
Supplement: Attachment — Submitted filename: Response to Reviewers.docx [file pone.0335890.s008.docx]

Dear Editor and Reviewers,

First, we would like to thank you for your kind letter and for reviewers' constructive comments concerning our article. These comments are all valuable and helpful for improving our article. All the authors have seriously discussed about all these comments. According to the reviewers' comments, we have modified our manuscript to meet with the requirements of your journal. In this revised version, changes to our manuscript within the document are highlighted by using red-colored text. Point-by-point responses to the reviewers are listed below in this letter.

**Reviewer 1:**

**Comment 1:**

*It is recommended to supplement the encapsulation efficiency and drug loading data of nanoparticles.*

**Response 1** :

We fully agree with your perspective that encapsulation efficiency (EE) and drug loading (DL) are two critical parameters for evaluating the performance of a nano-drug delivery system. As requested, we hereby provide the detailed data for the Exo-BSA@dBET6 nanoparticles: the **Encapsulation Efficiency (EE) is 56%**, and the **Drug Loading (DL) is 2.7%**. We have now incorporated these key data into the Results section of the manuscript.

**The revised objective now reads:(Page 9, Line 3-4)**

As shown in Figure 1a, in the transmission electron microscope (TEM) image, Exo-BSA@dBET6 presents a spherical vesicle structure. As shown in Figure 1d, the average diameter of Exo-BSA@dBET6 is 85.89nm. TEM provides precise measurements of individual particle dimensions, thereby revealing detailed morphological characteristics. In contrast, DLS yields an intensity-weighted hydrodynamic diameter that represents the average size of the entire particle population in solution. The observed discrepancy is further supported by the sample's polydispersity index (PDI = 0.275). The zeta potential is -30 mV, which suggests that the particle can remain stably dispersed in an aqueous solution over an extended period， and the polydispersity index (PDI) is 0.275, suggesting a relatively homogeneous size distribution. The encapsulation efficiency (EE) of Exo-BSA@dBET6 is 56%, and the drug loading (DL) capacity is 2.7%.

**Comment 2 :**

*Hemolysis test and plasma stability testing should be added.*

**Response 2:**

We sincerely thank the reviewer for these valuable suggestions. We have performed the recommended experiments to comprehensively evaluate the biocompatibility and stability of Exo-BSA@dBET6.

**The revised objective now reads:**

1. **Hemolysis Test**

**Methods: (Page 6, Line 6-17)**

Take a certain amount of defibrinated sheep blood (Solabio, Cat：TX0030), centrifuge at 400 g for 10 minutes, and then separate and retain the red blood cells. Then resuspend the red blood cells with an excess of normal saline, centrifuge at 400 g for 15 minutes, and repeat this step until the upper layer of normal saline after centrifugation is clear and transparent. Subsequently, resuspend with an appropriate amount of normal saline to obtain a 4% red blood cell suspension. Add 200 μL of red blood cell suspension and 200 μL of the corresponding concentration of the drug (normal saline as the negative control and 0.1% Triton as the positive control) to 1.5 mL EP tubes, mix well, and incubate at 60 rpm for 30 minutes at 37 ° C. Centrifuge at 400 g for 10 minutes and take photos for record. Then, take 100 μL of the supernatant into a 96-well plate and measure the OD value at a wavelength of 480 nm.

**Results: (Page 9, Line 21-26)**

The nanoparticles were analyzed by SDS-PAGE electrophoresis and Coomassie Blue staining (Figure 1c), indicating that the main component of the nanoparticles has a molecular weight similar to that of BSA. Additionally, the exosome-coated nanoparticles exhibited more bands corresponding to exosome-associated proteins compared to BSA. WB analysis also detected these exosomal markers (CD63 and CD9) (Figure 1e), indicating that we successfully constructed BSA nanoparticles coated with exosomes. To evaluate the hematocompatibility of Exo-BSA@dBET6, a hemolysis assay was performed using sheep red blood cells. The nanoparticles were incubated with erythrocytes at various concentrations, and the hemolytic activity was quantitatively and qualitatively assessed. As shown in Figure 1f, no significant hemolysis was observed at any tested concentration, confirming the excellent blood compatibility of Exo-BSA@dBET6.

Figure 1（F） Hemolysis test of Exo-BSA@dBET6 at various concentrations. Saline and 0.1% Triton X-100 were used as negative and positive controls, respectively. No significant hemolysis was observed in the nanoparticle groups.

2. **Plasma Stability Testing**  **(Page 9, Line 5-14)**

To evaluate the stability of Exo-BSA@dBET6, we selected phosphate buffered saline (PBS) with a pH of 7.4 as the incubation medium to simulate physiological conditions. The reason for not choosing plasma was to avoid interference from abundant plasma proteins, which might interfere with the quality of TEM imaging and affect the recognition of nanoparticles. Under the physiologically relevant conditions, Exo-BSA@dBET6 was incubated at 37°C for 24 hours and 72 hours, and then examined by TEM. As shown in Figure 1b, at both time points, the nanoparticles maintained their structural integrity without obvious morphological changes, indicating their excellent stability. These results suggest that Exo-BSA@dBET6 has strong stability.

Figure 1（B）Stability evaluation of Exo-BSA@dBET6 in PBS. TEM images of Exo-BSA@dBET6 nanoparticles after incubation in pH 7.4 PBS at 37 °C for 24 hours and 72 hours.

**Comment 3:**

*It is suggested to set up a control group of free exosomes + free dBET6 to exclude the effect of simple physical mixing.*

**Response 3:**

Thank you for this valuable suggestion. To exclude the potential effects of simple physical mixing, we have followed your advice and included a control group of free exosomes + free dBET6 (physical mixture), and evaluated its cytotoxic effect using the CCK-8 assay.

**The revised objective now reads: (Page 12, Line 6-14)**

The above experiments have confirmed that exosomes encapsulation can enhance the cellular uptake of albumin nanoparticles. We then performed a series of activity experiments to verify the anti-tumor ability in vitro. As shown in Figure 3a, compared with the control group, the cell viability of dBET6, BSA@dBET6 and Exo-BSA@dBET6 groups was decreased in a dose-dependent manner. At the same concentration, Exo-BSA@dBET6 group had the best effect on cell viability, followed by BSA@dBET6 group, and dBET6 had the worst effect. To exclude the potential influence of simple physical mixing, a control group of free exosomes + free dBET6 (physical mixture) was included in the cytotoxicity assessment. As shown in Figure 3b, no statistically significant difference in cell viability was observed between the free dBET6 group and the free exosomes + free dBET6 physical mixture group (p > 0.05), indicating that simple physical mixing does not enhance the antitumor efficacy of dBET6, and conversely confirms that the superiority of Exo-BSA@dBET6 nanoparticles stems from their unique structural design rather than mere mixing of the components. Furthermore, the enhanced ability of Exo-BSA@dBET6 to promote tumor cell death compared with free dBET6 was confirmed by live and dead cell staining and the quantitative analysis of red and green fluorescence (p< 0.01) (Figure 3c).

Figure 3 (B) CCK8 assay showed that there was no statistically significant difference in cytotoxicity between the free dBET6 group and the free exosomes + free dBET6 physical mixture group (p > 0.05)

**Reviewer 2:**

**Comment 1:**

*The TEM image in Figure 1A is not consistent with Figure 1B. Is the “spherical vesicle structure” referring to the large dark feature in the center or the small aggregated dots in the background? If it is the latter, please provide a higher-magnification image to show the morphology more clearly.*

**Response 1:**

We sincerely thank the reviewer for this insightful comment. We have now replaced Figure 1a with a new, higher-magnification TEM image that more clearly shows the well-dispersed, spherical morphology of the Exo-BSA@dBET6 nanoparticles.

**The revised objective now reads: (Page 8, Line 28-34)**

As shown in Figure 1a, in the transmission electron microscope (TEM) image, Exo-BSA@dBET6 presents a spherical vesicle structure. As shown in Figure 1d, the average diameter of Exo-BSA@dBET6 is 85.89nm. TEM provides precise measurements of individual particle dimensions, thereby revealing detailed morphological characteristics. In contrast, DLS yields an intensity-weighted hydrodynamic diameter that represents the average size of the entire particle population in solution. The observed discrepancy is further supported by the sample's polydispersity index (PDI = 0.275). The zeta potential is -30 mV, which suggests that the particle can remain stably dispersed in an aqueous solution over an extended period， and the polydispersity index (PDI) was 0.275, suggesting a relatively homogeneous size distribution. The encapsulation efficiency (EE) of Exo-BSA@dBET6 was 56%, and the drug loading (DL) capacity was 2.7%.

Figure 1(A) Morphology of Exo-BSA@dBET6 under transmission electron microscopy.  (scale bar: 100 nm)

**Comment 2:**

*In Figure 1C, the authors claim that “the nanoparticles have a molecular weight similar to that of BSA.” However, this is not clearly shown in the SDS-PAGE results, as the BSA lane appears incorrectly loaded.*

**Response 2:**

Thank you very much for your careful review and valuable comments regarding the SDS-PAGE results in Figure 1c. We apologize for the confusion caused by the suboptimal loading of the BSA lane in the previous image and sincerely appreciate your feedback, which has significantly improved the clarity and accuracy of our data presentation. We have updated Figure 1c and its corresponding legend accordingly.

In our revised manuscript, we have repeated the SDS-PAGE experiment with optimized loading conditions. As shown in the modified Figure 1c, the **BSA standard lane** now displays a clear, single band, and its migration position is **highly consistent** with the predominant band in the **Exo-BSA@dBET6 nanoparticle** sample (both located near the ~66 kDa molecular weight marker). This result robustly confirms our conclusion that the main component of the nanoparticles indeed has a molecular weight similar to that of BSA, indicating that BSA successfully forms the core matrix of the nanoparticles.

**The revised figure is presented as follow. (Page 10)**

Figure 1 (C) The nanoparticles were subjected to SDS-PAGE electrophoresis and Coomassie Brilliant Blue staining.

**Comment 3:**

*The manuscript uses both “dbet6” and “dBET6.” Please confirm if they refer to the same compound and ensure consistent formatting throughout the text.*

**Response 3:**

Thank you very much for your meticulous review of our manuscript and for pointing out this important issue of nomenclature consistency.

You are absolutely correct that "DBET6" and "dBET6" refer to the exact same compound, the PROTAC BRD4 degrader used in our study. The inconsistency was an unintentional error in formatting during the writing process, and we apologize for this oversight.

In direct response to your comment, we have now thoroughly checked and standardized the nomenclature throughout the entire text: All instances of the compound's name have been uniformly formatted as **"dBET6"** (with a lowercase 'd' and uppercase 'BET').

All these changes have been **highlighted in red** in the revised manuscript for your easy reference.

**Comment 4:**

*In Figure 2B, please label each panel clearly.*

**Response 4:**

Thank you very much for your valuable suggestion. We sincerely apologize for this oversight. We have followed your advice and have now clearly labeled each panel in Figure 2B.

**The revised figure is presented as follow. (Page 11)**

Figure 2. Cellular uptake and pathway of Exo-BSA@dBET6

**Comment 5:**

*In Figure 3A, what is* *the unit of concentration used for the cytotoxicity assay? For the y-axis labeled “cell viability (%)” please verify that the scale correctly ranges from 0 to 100.*

**Response 5:**

Thank you very much for raising these critical points and for your meticulous review of the figure details. Your comments have significantly enhanced the accuracy and professionalism of our data presentation.

Firstly, regarding the concentration unit, we apologize for the oversight. In the cytotoxicity assay presented in Figure 3a, the unit of concentration for all drug treatment groups (free dBET6, BSA@dBET6, and Exo-BSA@dBET6) is **micromolar (μM)**. We have now clearly labeled the concentration unit as "μM" alongside the x-axis in the revised Figure 3a.

Secondly, regarding the range of the y-axis labeled "Cell viability (%)". We have adjusted the Y-axis scale to span from **0 to 100**.

**The revised figure is presented as follow. (Page 13)**

Figure 3(A) CCK8 assay results showed that Exo-BSA@dBET6 has the most significant inhibitory effect on the growth and proliferation of breast cancer cells.

**Comment 6:**

*The claim of an “enhanced ability of Exo-BSA@dBET6 to promote tumor cell death” in Figure 3B is not convincingly supported. Please include quantitative analysis, such as live/dead cell counts or other relevant measurements, to substantiate this conclusion.*

**Response 6:**

Thank you very much for your valuable comment. The point you raised is highly pertinent, and we agree that quantitative data would provide more robust support for our conclusion.

In direct response to your suggestion, we have now performed rigorous quantitative analysis on the results from Figure 3c (Calcein-AM/PI staining). The numbers of live (green fluorescence) and dead (red fluorescence) cells from multiple randomly selected fields were counted and statistically analyzed using ImageJ software. This precise statistical evidence strongly substantiates our conclusion that Exo-BSA@dBET6 possesses an "enhanced ability to promote tumor cell death."

**The revised objective now reads: (Page 12, Line 14-16)**

The above experiments have confirmed that exosomes encapsulation can enhance the cellular uptake of albumin nanoparticles. We then performed a series of activity experiments to verify the anti-tumor ability in vitro. As shown in Figure 3a, compared with the control group, the cell viability of dBET6, BSA@dBET6 and Exo-BSA@dBET6 groups was decreased in a dose-dependent manner. At the same concentration, Exo-BSA@dBET6 group had the best effect on cell viability, followed by BSA@dBET6 group, and dBET6 had the worst effect. To exclude the potential influence of simple physical mixing, a control group of free exosomes + free dBET6 (physical mixture) was included in the cytotoxicity assessment. As shown in Figure 3b, no statistically significant difference in cell viability was observed between the free dBET6 group and the free exosomes + free dBET6 physical mixture group (p > 0.05), indicating that simple physical mixing does not enhance the antitumor efficacy of dBET6, and conversely confirms that the superiority of Exo-BSA@dBET6 nanoparticles stems from their unique structural design rather than mere mixing of the components. Furthermore, the enhanced ability of Exo-BSA@dBET6 to promote tumor cell death compared with free dBET6 was confirmed by live and dead cell staining and the quantitative analysis of red and green fluorescence (p< 0.01) (Figure 3c).

CTL	dBET6	Exo-BSA@dBET6

**100μm**

Figure 3 (C) Calcein-AM/PI staining demonstrated that Exo-BSA@dBET6 was significantly more effective in promoting tumor cell death compared to dBET6.

**Reviewer 3:**

**Comment 1:** *Inadequate Nanoparticle Characterisation:*

*The manuscript includes only basic nanoparticle characterisation (TEM, DLS, SDS-PAGE, and Western blot for exosome markers). However, essential physicochemical parameters — such as zeta potential, polydispersity index (PDI), drug loading, encapsulation efficiency, and nanoparticle yield — are not reported.*

*Critically, no drug release studies are provided, which is a fundamental requirement for any study proposing a drug delivery system. Without drug release kinetics, the proposed benefit of sustained or targeted delivery remains entirely unverified.*

**Response 1:**

Thank you very much for these critical and essential comments. We fully agree that the physicochemical parameters and drug release studies you pointed out are indispensable for the comprehensive characterization of a nano-drug delivery system. We apologize for the omission of these data and have now conducted additional experiments and analyses as per your recommendations. As requested, we hereby provide the detailed data for the Exo-BSA@dBET6 nanoparticles: **the Encapsulation Efficiency (EE) is 56%, the Drug Loading (DL) is 2.7%, the zeta potential is -30 mV, and the polydispersity index (PDI) is 0.275**. We have now incorporated these key data into the Results section of the manuscript.

Regarding the study of **drug release**, we sincerely appreciate the reviewer for raising this crucial question. We fully agree that the kinetics of drug release is essential for characterizing drug delivery systems and apologize for the omission of this aspect in the current study. Unfortunately, due to unforeseen and persistent technical issues with our equipment and HPLC detection system, we were unable to obtain reliable and reproducible drug release data within the revision period for this manuscript. We recognize that this is a significant limitation.

However, we would like to highlight several lines of indirect evidence from our study that support the functional release and efficacy of dBET6 from our nanoparticles: 1.The significantly enhanced cytotoxicity and pro-apoptotic effects of Exo-BSA@dBET6 compared to free dBET6 (Figure 3) strongly suggest successful intracellular drug release. 2. The demonstrated cellular uptake and lysosomal escape capability (Figure 2)provides a mechanistic basis for drug release. 3. Western blot analysis confirmed superior target protein (BRD4) degradation following treatment with Exo-BSA@dBET6 (Figure 4A), indicating biological activity of the released drug.

We are currently working to resolve the equipment issues and establish a robust release assay. We thank the reviewer for emphasizing this important aspect and hope the current indirect evidence along with our planned future work adequately address this concern.

**The revised objective now reads: (Page 9, Line 1-4)**

As shown in Figure 1a, in the transmission electron microscope (TEM) image, Exo-BSA@dBET6 presents a spherical vesicle structure. As shown in Figure 1d, the average diameter of Exo-BSA@dBET6 is 85.89 nm. TEM provides precise measurements of individual particle dimensions, thereby revealing detailed morphological characteristics. In contrast, DLS yields an intensity-weighted hydrodynamic diameter that represents the average size of the entire particle population in solution. The observed discrepancy is further supported by the sample's polydispersity index (PDI = 0.275). The zeta potential is -30 mV, which suggests that the particle can remain stably dispersed in an aqueous solution over an extended period， and the polydispersity index (PDI) is 0.275, suggesting a relatively homogeneous size distribution. The encapsulation efficiency (EE) of Exo-BSA@dBET6 is 56%, and the drug loading (DL) capacity is 2.7%.

**Comment 2:** *Lack of Structural Confirmation of the Drug–Carrier Complex:*

*The manuscript lacks analytical evidence confirming the successful incorporation and structural integrity of dBET6 within the nanoparticles. Given that dBET6 is a small molecule PROTAC, its presence and stability in the system should be confirmed using appropriate analytical methods such as mass spectrometry, HPLC, or UV absorbance. This is especially critical when proposing a mechanism reliant on intracellular BRD4 degradation.*

**Response 2:**

We sincerely thank the reviewer for this critical comment regarding the structural confirmation of our drug-carrier complex. We fully agree that analytical verification of dBET6 incorporation and stability is highly important for characterizing our nano-formulation. We sincerely apologize for this omission in our current manuscript. Unfortunately, due to unforeseen and persistent technical failures of our core analytical equipment (specifically the HPLC-MS system), we were unable to perform the stability，within the revision timeframe.

However, we would like to provide the following evidence that indirectly but strongly supports the successful incorporation and functional delivery of dBET6: 1. **Structural Integrity Confirmation:** Transmission Electron Microscopy (TEM) images (Figure 1A, 1B) clearly demonstrate the well-defined spherical structure and physical integrity of the Exo-BSA@dBET6 nanoparticles, providing essential morphological evidence of nanoparticle stability. 2. **Drug Loading and Encapsulation Efficiency:** We have provided quantitative data on the encapsulation efficiency (56%) and drug loading capacity (2.7%) of Exo-BSA@dBET6, which confirms the successful incorporation of dBET6 into the nanoparticles.

We completely agree with the reviewer that direct analytical confirmation is essential. Therefore, addressing this point is a top priority for our ongoing work. We are actively working to resolve the equipment issues and will perform detailed characterization using HPLC-MS/MS in our follow-up study.

**Comment 3:** *No Description of Albumin Nanoparticle Preparation:*

*It is unclear whether the albumin nanoparticles were prepared by the authors or sourced commercially. If prepared in-house, no methodology or optimisation strategy is described, making the system irreproducible and scientifically unverifiable. This is a major concern for a research article aiming to establish a novel delivery platform.*

**Response 3:**

Thank you very much for raising this critical point. The methodological details you highlighted are indeed essential for ensuring the reproducibility and scientific rigor of the study.

The **milk exosomes, BSA@dBET6 nanoparticles, and the final Exo-BSA@dBET6 hybrid nanoparticles** used in this study were **all prepared in-house by our research group** in the laboratory, not sourced commercially. We apologize for not making this clear in the initial manuscript and for omitting the key optimization strategies and details.

We have now provided a comprehensive description of the entire preparation process and critical parameters in the **Methods section** of the revised manuscript, ensuring that any laboratory can rigorously replicate this procedure.

**The revised objective now reads: (Page 4, Line 14-36; Page 5, Line 1-11)**

Preparation of milk exosome：

Centrifuge bovine milk samples at 3000 g for 30 minutes at 4 °C to remove fat globules, casein aggregates, and other debris. Carefully collect the supernatant and subject it to centrifugation at 10,000 g for 60 minutes at 4 °C to eliminate larger cellular debris and vesicles. Following this, collect the supernatant and filter it through a 0.22 μm membrane filter. Subsequently, perform ultracentrifugation at 100,000 g for 120 minutes at 4 °C to pellet the exosomes. Discard the supernatant, and promptly wash the exosome pellet with phosphate-buffered saline (PBS) to remove residual debris and microvesicles. Finally, collect the purified exosome pellet, resuspend it in a small volume of PBS, and store it at −80 °C for subsequent analysis.

Preparation of BSA@dBET6:

A defined quantity of BSA was dissolved in 2 mL of water, and the pH was adjusted to 8.0–9.0 using a 3 mol/L sodium hydroxide solution, dBET6 was dissolved in anhydrous ethanol and then added to the BSA solution at a controlled rate of 0.2 mL/min（drug-to-BSA mass ratio is 1:15）. Subsequently, anhydrous ethanol was added dropwise such that the final volume ratio of water to anhydrous ethanol reached 1:4, while the solution was continuously stirred at 600–800 rpm. A solution of BAC in anhydrous ethanol was then introduced, with a BSA-to-BAC mass ratio of 10:1. The resulting mixture was stirred continuously at 50 °C for 24 hours. Afterward, the ethanol was removed via evaporation, and the product was purified by centrifugation to yield BSA@dBET6.

Preparation of Exo-BSA@dBET6:

Dissolve 10 mg of BSA@dBET6 in 2 mL of PBS solution at pH 7.4, and disperse the mixture uniformly via ultrasonication. Subsequently, add 1 mL of milk-derived exosomes and thoroughly mix the solution. Place the resulting mixture in an ice bath and subject it to ultrasonic cell disruption under controlled conditions (2 kHz, 10% amplitude, 3 cycles of 3 minutes each with 2-minute intervals between cycles). Following ultrasonication, incubate the sample at 37 °C for 1 hour. Centrifuge the sample at 10,000 rpm for 5 minutes at 4 °C, discard the supernatant, and wash the pellet with PBS buffer three times. The final product, designated as Exo-BSA@dBET6, is obtained and subsequently freeze-dried for long-term storage.

**Comment 4:** *Limited Mechanistic and Functional Validation:*

*Although the manuscript reports in vitro uptake and cytotoxicity assays, the data are largely descriptive. There is no quantitative statistical analysis (e.g., error bars, p-values, replicate numbers), nor are proper negative or vehicle controls described.*

*The transcriptomic data presented are not validated through downstream experiments such as qPCR, pathway inhibition, or gene silencing, which significantly limits the depth of mechanistic insight.*

**Response 4:**

1.**Regarding quantitative statistics and negative and carrier controls**

We thank the reviewer for this important comment regarding the statistical analysis and experimental controls in our study. We sincerely apologize for the oversight in clearly presenting these essential details in our initial submission. We have now performed comprehensive statistical analysis and included all necessary controls to strengthen our conclusions.

Firstly, we have now added error bars (representing mean ± SD) and included p-values in Figure 3A (CCK-8 assay) and Figure 3C (quantified Calcein-AM/PI staining) to demonstrate statistical significance. All experiments were performed with at least three independent replicates (n ≥ 3).

Secondly, we have included proper control groups: 1. Negative control: Untreated cells (medium only). 2. Physical mixture control: To exclude the potential influence of simple physical mixing, cells were exposed to a combination of free exosomes and free dBET6 (Figure 3B).

These additions significantly strengthen our conclusions and provide proper statistical validation of our findings. We have revised the figure legends and Methods section to explicitly describe the number of replicates, statistical methods used, and nature of controls included. For your convenience in reviewing the document, we have marked the newly added content in red.

**The revised objective now reads: (Page 12 , Line 6-16)**

The above experiments have confirmed that exosomes encapsulation can enhance the cellular uptake of albumin nanoparticles. We then performed a series of activity experiments to verify the anti-tumor ability in vitro. As shown in Figure 3a, compared with the control group, the cell viability of dBET6, BSA@dBET6 and Exo-BSA@dBET6 groups was decreased in a dose-dependent manner. At the same concentration, Exo-BSA@dBET6 group had the best effect on cell viability, followed by BSA@dBET6 group, and dBET6 had the worst effect. To exclude the potential influence of simple physical mixing, a control group of free exosomes + free dBET6 (physical mixture) was included in the cytotoxicity assessment. As shown in Figure 3b, no statistically significant difference in cell viability was observed between the free dBET6 group and the free exosomes + free dBET6 physical mixture group (p > 0.05), indicating that simple physical mixing does not enhance the antitumor efficacy of dBET6, and conversely confirms that the superiority of Exo-BSA@dBET6 nanoparticles stems from their unique structural design rather than mere mixing of the components. Furthermore, the enhanced ability of Exo-BSA@dBET6 to promote tumor cell death compared with free dBET6 was confirmed by live and dead cell staining and the quantitative analysis of red and green fluorescence (p< 0.01) (Figure 3c).

Figure 3. Antitumor activities of Exo-BSA@dBET6. (A) CCK8 assay results showed that Exo-BSA@dBET6 has the most significant inhibitory effect on the growth and proliferation of TNBC cells. Data are presented as mean ± SD (n = 3). *p < 0.05, **p < 0.01, ***p < 0.001 vs. control group. (B) CCK8 assay showed that there was no statistically significant difference in cytotoxicity between the free dBET6 group and the free exosomes + free dBET6 physical mixture group (p > 0.05). Data are presented as mean ± SD (n = 3). NS: not significant. (C) Calcein-AM/PI staining demonstrated that Exo-BSA@dBET6 was significantly more effective in promoting tumor cell death compared to dBET6. (D、E) Caspase-3 staining and JC-1 staining results showed that Exo-BSA@dBET6 has the capability to significantly enhance apoptosis of MDA-MB-231 cells. (F) The ROS staining results showed that the ROS concentration in Exo-BSA@dBET6 group was significantly increased compared with dBET6 group.

**2.Regarding *an in-depth investigation into the underlying mechanisms***

We sincerely thank the reviewer for this insightful comment regarding the validation of our transcriptomic data. We agree that additional validation through qPCR, pathway inhibition, or gene silencing experiments would provide deeper mechanistic insights into the anti-tumor mechanisms of Exo-BSA@dBET6. We fully recognize the importance of these orthogonal validation methods. At the same time, it is worth noting that high-throughput transcriptomic sequencing has become a well-established and widely accepted methodology in its own right for genome-wide expression profiling. While PCR validation remains a common practice, modern RNA-Seq workflows—when performed with adequate replicates, rigorous quality control, and appropriate bioinformatic analysis—are generally considered sufficient to draw reliable biological conclusions. Our extensive bioinformatic examination—encompassing differential gene expression, pathway enrichment, and molecular network analysis—has yielded coherent and thorough mechanistic understanding that is internally consistent and scientifically informative.

We acknowledge that the lack of such validation in the current study is a limitation. Unfortunately, due to time and technical constraints during the revision period, we were unable to complete these additional experiments. However, we have now explicitly addressed this limitation in the Discussion section of our revised manuscript.

We agree with the reviewer that these validation experiments are important, and we are currently designing follow-up studies that will include: 1. qPCR validation of key differentially expressed genes. 2. Pathway inhibition studies using specific inhibitors of the PI3K-Akt and Rap1 signaling pathways. 3. Gene silencing experiments to confirm the functional relevance of identified targets.

**The revised objective now reads: (Page 17 , Line 12-16)**

The major limitation of the present study is the lack of animal experiments, future research should be undertaken to explore the anti-tumor effect of Exo-BSA@dBET6 in vivo. Additionally, while our transcriptomic analysis revealed significant alterations in key signaling pathways such as PI3K-Akt and Rap1, the lack of validation through qPCR or functional studies represents a limitation of this study. Future work will include experimental validation of these findings through pathway inhibition assays and gene expression analysis to provide deeper mechanistic insights.

**Comment 5:** *Absence of Stability and In Vivo Data:*

*No stability data are provided (e.g., nanoparticle integrity in serum or under physiological pH), which is necessary even in the absence of animal work. The in vivo limitation is acknowledged, but the complete omission of in vitro stability studies further weakens the case for translational relevance.*

**Response 5:**

We sincerely thank the reviewer for raising this important point regarding the stability assessment of our nanoparticles. We agree that stability data are essential for evaluating the translational potential of any nanomedicine formulation, even in the absence of in vivo studies. In response to this comment, we have now conducted additional stability studies to address this concern.

**The revised objective now reads: (Page 9 , Line 5-14)**

To evaluate the stability of Exo-BSA@dBET6, we selected phosphate buffered saline (PBS) with a pH of 7.4 as the incubation medium to simulate physiological conditions. The reason for not choosing plasma was to avoid interference from abundant plasma proteins, which might interfere with the quality of TEM imaging and affect the recognition of nanoparticles. Under the physiologically relevant conditions, Exo-BSA@dBET6 was incubated at 37°C for 24 hours and 72 hours, and then examined by TEM. As shown in Figure 1b, at both time points, the nanoparticles maintained their structural integrity without obvious morphological changes, indicating their excellent stability. These results suggest that Exo-BSA@dBET6 has strong stability.

Figure 1（B）Stability evaluation of Exo-BSA@dBET6 in PBS. TEM images of Exo-BSA@dBET6 nanoparticles after incubation in pH 7.4 PBS at 37 °C for 24 hours and 72 hours.

**Comment 6:** *Incorrect Referencing in the Introduction:*

*The epidemiological statistics quoted regarding breast cancer incidence do not match the cited source and should be corrected.*

**Response 6:**

Thank you very much for your meticulous review of our manuscript and for identifying this important citation error. We sincerely apologize for this oversight.

Following your comment, we have carefully verified and corrected the corresponding statement and its reference in the Introduction section.

**The revised objective now reads: (Page 2 , Line 28-30)**

Breast cancer is the most common malignancy among women worldwide, accounting for 32% of cancers diagnosed in women and the leading cause of cancer death among women aged 20 to 49 years[1]. Among these, triple-negative breast cancer (TNBC) exhibits a particularly poor prognosis. More than 50% of patients relapse within the first 3 to 5 years after diagnosis[2]. Due to the absence of relevant receptor markers, TNBC patients do not benefit from established endocrine therapies or HER2-targeted agents. Thus, the predominant treatment for nonsurgical TNBC remains non-specific chemotherapy. However, it is associated with significant limitations, including drug resistance, severe adverse effects, and heterogeneous treatment responses. Consequently, there is an urgent need to develop targeted therapies for the effective management of TNBC. Bromodomain and extra-terminal (BET) family proteins play a crucial role as epigenetic modifiers. As the most extensively expressed protein within the BET family, BRD4 plays a central role in regulating numerous molecular and cellular processes[3]. In recent years, several studies have demonstrated that BRD4 plays a key role in cell proliferation, survival and metastasis of breast cancer, and can be used as an emerging therapeutic target for down-regulating TNBC[4-6]. Small-molecule BET inhibitors have shown preclinical efficacy, however, their clinical application has been limited by the development of drug resistance and the occurrence of off-target effects.

**Updated Reference: (Page 17 , Line 27-28)**

1. Siegel, R.L., Giaquinto, A.N. & Jemal, A. Cancer statistics, 2024. *CA: A Cancer Journal for Clinicians* 74, 12–49 (2024). <http://doi.org/10.3322/caac.21820>

This reference explicitly provides the precise statistical data we have cited (please see the ' Expected number of new cancer cases' section of this article), thereby ensuring the accuracy and authority of our argument.

**Comment 7：***Poor Paragraph Transitions:*

*Several sections in the introduction lack logical flow, particularly the abrupt shift from disease context to PROTACs and nanoparticle systems.*

**Response 7:**

Thank you very much for your valuable feedback. The issue you raised regarding the logical flow between paragraphs in the Introduction, particularly the abrupt transition from the disease context to PROTAC technology and nanoparticle systems, is crucial for improving the quality of our manuscript.

In response to your suggestion, we have revised the Introduction to specifically strengthen the logical connection between "the challenges in TNBC therapy and the limitations of BET inhibitors" and "the introduction of PROTAC technology as a solution." These modifications are intended to clearly establish the cause-and-effect relationship: "existing therapies have drawbacks (BET inhibitors) → thus, a new technology (PROTAC) is required to overcome them." This allows the narrative to transition naturally from the broad disease background to the specific technological solution.

**The revised objective now reads: (Page 3 , Line 6-11)**

Breast cancer is the most common malignancy among women worldwide, accounting for 32% of cancers diagnosed in women and the leading cause of cancer death among women aged 20 to 49 years[1]. Among these, triple-negative breast cancer (TNBC) exhibits a particularly poor prognosis. More than 50% of patients relapse within the first 3 to 5 years after diagnosis[2]. Due to the absence of relevant receptor markers, TNBC patients do not benefit from established endocrine therapies or HER2-targeted agents. Thus, the predominant treatment for nonsurgical TNBC remains non-specific chemotherapy. However, it is associated with significant limitations, including drug resistance, severe adverse effects, and heterogeneous treatment responses. Consequently, there is an urgent need to develop targeted therapies for the effective management of TNBC. Bromodomain and extra-terminal (BET) family proteins play a crucial role as epigenetic modifiers. As the most extensively expressed protein within the BET family, BRD4 plays a central role in regulating numerous molecular and cellular processes.^3^ In recent years, several studies have demonstrated that BRD4 plays a key role in cell proliferation, survival and metastasis of breast cancer, and can be used as an emerging therapeutic target for down-regulating TNBC[4-6]. Small-molecule BET inhibitors have shown preclinical efficacy, however, their clinical application has been limited by the development of drug resistance and the occurrence of off-target effects.

To overcome these limitations, proteolysis-targeting chimera (PROTAC) technology has gained traction as a novel approach to degrade target proteins selectively. PROTAC technology is a kind of targeted protein degradation technology using ubiquititation-proteasome system (UPS), leading to the proteasomal degradation of the ubiquitinated target protein. In recent years, this technology has garnered significant attention due to its capability to target undruggable proteins[7-8]. DBET6, a PROTAC-based BRD4 degrader, effectively and continuously degrades BRD4 protein, thereby exerting potent antitumor effects， such as breast cancer, lung cancer, etc[9-10]. However, the limited tissue selectivity and low bioavailability of dBET6 significantly restrict its clinical application. Therefore, it is a critical issue to optimize the dBET6 drug delivery system.

**Comment 8：***Unclear Exosome Origin:*

*The source of the exosomes is ambiguously reported. Whether they are derived from TNBC cells, camel milk (as mentioned in a cited study), or another origin is not specified. This significantly impacts the interpretation of the targeting efficacy.*

**Response 8:**

We sincerely thank the reviewer for this important comment regarding the source of exosomes used in our study. We apologize for any ambiguity in our original description.

The exosomes used in our study were indeed derived from bovine milk, as explicitly described in the Methods section under "Preparation of milk exosome": "Centrifuge bovine milk samples at 3000 g for 30 minutes at 4 °C to remove fat globules, casein aggregates, and other debris...".

We selected milk-derived exosomes for several important reasons: 1. Biocompatibility and widespread availability: Milk exosomes are natural, biocompatible nanovesicles that can be obtained in large quantities. 2. Targeting potential: Importantly, milk is secreted by bovine mammary gland cells and consequently, milk-derived exosomes carry surface proteins that resemble those of mammary tissue cells. This biological characteristic suggests their potential natural targeting affinity for breast cancer cells, including TNBC cells.

We have now clarified this point in the revised manuscript (Introduction and Methods sections) to ensure readers understand both the source of our exosomes and the rationale for this selection.

**The revised objective now reads: (Page 3 , Line 28-35)**

Exosomes are cell-derived nanovesicles that play a crucial role in the intercellular transport of substances and are currently being investigated as promising endogenous nanocarriers[11-12]. Therapeutic drugs, such as small molecule or nucleic acid drugs, can be integrated into exosomes and then delivered to specific types of cells or tissues due to homing effects for targeted drug delivery[12-14]. In addition, exosomes can also be used to deliver PROTAC molecules[15-16]. Notably, A. Nathani utilized camel milk-derived exosomes as a delivery platform for ARV-825, thereby enhancing its efficacy in cancer therapy[17]. In the present study, we selected bovine milk-derived exosomes as the coating material for our nanoplatform. Milk is secreted by mammary gland cells, and consequently, milk-derived exosomes naturally carry surface proteins that resemble those of mammary tissue cells. This biological characteristic suggests their potential intrinsic targeting affinity for breast cancer cells, including TNBC. Additionally, milk exosomes offer significant advantages of biocompatibility, widespread availability, ease of acquisition, and scalability compared to cell culture-derived exosomes.

**Preparation of milk exosome：(Page 4 , Line 14-26)**

The exosomes used in this study were derived from bovine milk, which were selected due to their mammary gland origin, potential targeting affinity for breast cancer cells, and practical advantages including biocompatibility and scalability. Centrifuge bovine milk samples at 3000 g for 30 minutes at 4 °C to remove fat globules, casein aggregates, and other debris. Carefully collect the supernatant and subject it to centrifugation at 10,000 g for 60 minutes at 4 °C to eliminate larger cellular debris and vesicles. Following this, collect the supernatant and filter it through a 0.22 μm membrane filter. Subsequently, perform ultracentrifugation at 100,000 g for 120 minutes at 4 °C to pellet the exosomes. Discard the supernatant, and promptly wash the exosome pellet with phosphate-buffered saline (PBS) to remove residual debris and microvesicles. Finally, collect the purified exosome pellet, resuspend it in a small volume of PBS, and store it at −80 °C for subsequent analysis.

**Comment 9:** *Language and Formatting Issues:*

*The manuscript requires extensive English language editing. Inconsistent terminology (e.g., “dbet6” vs “dBET6”) and multiple grammatical errors detract from clarity.*

**Response 9:**

Thank you very much for your meticulous review of our manuscript and for pointing out this important issue of nomenclature consistency. The inconsistency was an unintentional error in formatting during the writing process, and we apologize for this oversight.

In direct response to your comments, we have conducted a thorough language check and revision of the entire manuscript. All instances of the compound's name have been uniformly formatted as **"dBET6"** (with a lowercase 'd' and uppercase 'BET') throughout the text. All these changes have been **highlighted in red** in the revised manuscript for your easy reference.
